# Synthesis and Catalytic Activity for 2, 3, and 4-Nitrophenol Reduction of Green Catalysts Based on Cu, Ag and Au Nanoparticles Deposited on Polydopamine-Magnetite Porous Supports

**DOI:** 10.3390/nano13152162

**Published:** 2023-07-25

**Authors:** Helen K. Brown, Jamal El Haskouri, María D. Marcos, José Vicente Ros-Lis, Pedro Amorós, M. Ángeles Úbeda Picot, Francisco Pérez-Pla

**Affiliations:** 1 Institut de Ciència dels Materials (ICMUV), c/Catedrático José Beltrán 2, Paterna, 46980 Valencia, Spain; h.k.brown1@newcastle.ac.uk (H.K.B.); haskouri@uv.es (J.E.H.); pedro.amoros@uv.es (P.A.); 2 Centro de Reconocimiento Molecular y Desarrollo Tecnológico (IDM), Unidad Mixta Universitat Politècnica de València-Universitat de València, Departamento de Química, Universitat Politècnica de València, Camino de Vera s/n, 46022 Valencia, Spain; mmarcos@qim.upv.es; 3 Centro de Reconocimiento Molecular y Desarrollo Tecnológico (IDM), Unidad Mixta Universitat Politècnica de València-Universitat de València, Universitat de València, Dr. Moliner 50, 46100 Burjassot, Spain; j.vicente.ros@uv.es; 4 Departamento de Química Inorgànica, Universitat de València, Dr. Moliner 50, Burjassot, 46100 València, Spain

**Keywords:** Cu, Ag, Au nanoparticles, polydopamine, magnetite, nitrophenol reduction

## Abstract

This work reports on the synthesis of nine materials containing Cu, Ag, Au, and Ag/Cu nanoparticles (NPs) deposited on magnetite particles coated with polydopamine (PDA). Ag NPs were deposited on two PDA@Fe_3_O_4_ supports differing in the thickness of the PDA film. The film thickness was adjusted to impart a textural porosity to the material. During synthesis, Ag(I) was reduced with ascorbic acid (HA), photochemically, or with NaBH_4_, whereas Au(III), with HA, with the PDA cathecol groups, or NaBH_4_. For the material characterization, TGA, XRD, SEM, EDX, TEM, STEM-HAADF, and DLS were used. The catalytic activity towards reduction of 4-, 3- and 2-nitrophenol was tested and correlated with the synthesis method, film thickness, metal particle size and NO_2_ group position. An evaluation of the recyclability of the materials was carried out. In general, the catalysts prepared by using soft reducing agents and/or thin PDA films were the most active, while the materials reduced with NaBH_4_ remained unchanged longer in the reactor. The activity varied in the direction Au > Ag > Cu. However, the Ag-based materials showed a higher recyclability than those based on gold. It is worth noting that the Cu-containing catalyst, the most environmentally friendly, was as active as the best Ag-based catalyst.

## 1. Introduction

Due to the fast growth of the world population and the rapid development of modern industry, the supply of clean and safe water is now a serious worldwide concern. Water pollution with organic chemicals, toxic inorganic elements and microorganisms has a negative impact on human life and the health of aquatic habitats and plant species [1,2,3,4,5,6,7,8].

Among the organic pollutants phenol and its nitro derivatives are considered the most environmentally hazardous [9]. Their use in the chemical industry for the manufacturing of pharmaceuticals, paper, pesticides, dyes, pigments, explosives, plasticizers, and fungicides generates each year a large quantity of these polluting wastes, which are difficult to degrade due their biological and chemical stability [10,11]. 4-Nitrophenol (4-NP) is reported as a mutagenic/carcinogenic, anthropogenic, xenobiotic and teratogenic compound, even at low concentrations (20–100 μg/L). Its solubility in water systems and its stability facilitates its accumulation, causing damage to the blood, kidneys, liver and nervous system of humans and animals [11,12,13]. The United States Environmental Protection Agency (US-EPA) has listed 4-NP as one of the 114 priority organic pollutants and The China National Environmental Monitoring Center considers it a priority control, with 60 ppb being the maximum content of 4-NP in direct drinking water [1,14].

It is necessary to solve this ecological problem by using physical, thermal, biological, or chemical treatments [8]. The catalytic reduction of nitroarenes is one of the most efficient chemical processes and the aniline and its derivatives obtained account for a large share of the organic chemistry market. 4-NP is an intermediate compound in the synthesis of benorilate and paracetamol, two potential antipyretic and analgesic replacements of aspirin and phenacetin, which are components of non-steroidal anti-inflammatory medications around the world [2,11].

Catalysts based on nanoparticles of precious metals (Pd, Pt, Au, Ag) have been shown to be very efficient in the reduction of nitrophenols by a reducing agent, although in the last decade, cheaper alternatives with metal nanoparticle catalysts of inexpensive and more abundant 3d metals (Co, Ni and Cu) have been used successfully [15,16,17,18,19,20,21,22,23,24,25,26].

Metal nanoparticles (M NPs) with a high area-to-volume ratio provide many active sites to interact with the substrate during the catalytic process, but, because of their high surface energy, tend to aggregate during the reaction which entails a decrease in its catalytic capacity. In addition, their nanoscale size makes them difficult to separate from the reaction system for their reusability [1,2,27,28]. Different supporting materials have been used to stabilize M NPs, preventing the undesirable agglomeration: mesoporous silica, graphene, graphene oxide, polymers, porous carbon, covalent-organic frameworks (COFs), or Fe_3_O_4_ that allows the easy recyclability of the catalyst.

An interesting approach to the synthesis of efficient M NP-supported catalyst involves the modification of the support, aimed at generating a suitable environment that controls the charge and dispersion of the M NPs, avoiding their aggregation and leaching. The polydopamine (PDA) synthesized by Lee and coworkers exhibits strong adhesion on virtually any surface, has the ability to coordinate metal ions by their functional groups, and at the same time, can reduce them via their catechol groups [29,30,31,32]. The PDA coating on the support can drive the interfacial assembly of M NPs, stabilizing them [11]. Different catalysts containing Cu NPs [33,34,35,36], Ag NPs [37,38,39,40,41,42,43] and Au NPs [44,45,46,47,48,49,50,51,52,53,54,55,56,57,58]/PDA/support (or not) have been described in the literature. In our laboratory, Pd NPs have been anchored on a variety of different supports. In particular, Pd anchored on PDA@SiO_2_ or PDA@Fe_3_O_4_ have proven to be very efficient in reducing nitroarenes [11,59].

In this work, different porous catalysts containing group 11 metal NPs (Cu, Ag and Au) have been synthesized using PDA as a linker on the Fe_3_O_4_ magnetic support. A thorough characterization has been carried out using a variety of techniques to understand the micro- and nanoscale morphology of our catalysts. Their catalytic activity in the reduction of 4-, 3- or 2-nitrophenol and the recyclability of 4-nitrophenol have been studied. In addition, an important effort has been made to extract information from the bibliography and determine TOF values that allow us to compare and objectively evaluate our materials.

## 2. Materials and Methods

### 2.1. Materials

Commercially available methanol (Labkem, Barcelona, Spain), acetonitrile (Labkem), ammonia (Scharlau), (NH_4_)_2_Fe(SO_4_)_2_·6H_2_O (Labkem), (NH_4_)Fe(SO_4_)_2_·12H_2_O (Labkem), dopamine hydrochloride (Sigma-Aldrich, Madrid, Spain), Trizma chlorhidrate (Sigma-Aldrich), 4, 3, and 2-nitrophenol (Sigma-Aldrich), ascorbic acid (Sigma-Aldrich), AgNO_3_ (Panreac, Barcelona, Spain), HAuCl_4_·3H_2_O (Sigma-Aldrich), Cu(II) acetate monohydrate (Fluka, Madrid, Spain), Cu(II) nitrate trihydrate (Fluka), and NaBH_4_ (Sigma-Aldrich) were used without further purification.

### 2.2. Characterization Techniques

- SEM (Scanning Electron Microscopy) microstructural characterizations were carried out using a JEOL JEM-1010 instrument with a CCD camera operating at 100 kV. This instrument was also used to determine the metal contents by energy dispersive X-ray spectroscopy (EDX) analysis.
- The TEM, STEM-HAADF, and mapping of different elements (by using an EDS X-ray detector) was carried out using a JEOL-2100F microscope operated at 200 kV. For electron microscopy analyses, the samples were dispersed in ethanol and placed onto a carbon-coated nickel microgrid and left to dry before observation.
- TGA (Thermal Gravimetric Analysis) curves were recorded using a Setaram Setsys 16/18 thermobalance with a heating rate of 5 ${}^{\circ }$C/min under an air flowing atmosphere of 25 mL/min.
- XRD (Powder X-ray Diffraction) was carried out using a Bruker D8 Advance diffractometer with monochromatic Kα source operated at 40 kV and 40 mA. Patterns were collected in steps of 0.02${}^{\circ }$(2θ) over the angular range 1${}^{\circ }$–10${}^{\circ }$(2θ) with an acquisition time of 25 s per step.
- Nitrogen adsorption–desorption isotherms were recorded in an automated Micromeritics ASAP2020 instrument. Prior to the adsorption measurements, the samples were outgassed in situ under vacuum ($10^{-6}$ Torr) at 120 ${}^{\circ }$C for 15 h to remove adsorbed gases.
- DLS (Dynamic Light Scattering) photonic correlation diagrams used to determine hydrodynamic radii were measured using a Zetasizer nano series instrument (from Malvern Instruments).
- Absorbance was monitored to track reaction progress using a diode-array UV/Vis Agilent 8453 spectrometer.
- HPLC mixture resolutions were carried out using a JASCO HPLC modular chromatograph equipped with a C18 polar column (Kinetex, 100 mm × 4.6 mm, 2.6 μm particle size from Phenomenex) fitted to a column guard, a quaternary pump (PU-2089+), column oven (CO-2065+), and a diode-array detector (MD-2018+).

### 2.3. Preparation of M NPs-PDA@Fe_3_O_4_ Catalysts (M = Cu, Ag, Au, Ag/Cu)

#### 2.3.1. Synthesis of Fe_3_O_4_

In a 50 mL round bottomed flask, (NH_4_)_2_Fe(SO_4_)_2_·6H_2_O (8.38 g, 21.3 mmol) and (NH_4_)Fe(SO_4_)_2_·12H_2_O (8.41 g, 17.4 mmol) were dissolved in water (25 mL). The solution was heated to 60 ${}^{\circ }$C and refluxed under nitrogen for 30 min until fully dissolved. A solution of H_2_O:NH_3_ (15 mL NH_3_ 32% *w*/*w* in 10 mL H_2_O) was added using a perfusion pump (0.5 mL/min). This suspension was kept at 100 ${}^{\circ }$C and left to stir under nitrogen overnight. When the suspension had cooled to room temperature, it was transferred to a 100 mL beaker and the solid Fe_3_O_4_ was separated using a magnet. The black solid was washed with water until a colorless washing was obtained, then washed with methanol, and dried at 70 ${}^{\circ }$C for 48 h (1.62 g).

#### 2.3.2. Coating Fe_3_O_4_ with Polydopamine (PDA@Fe_3_O_4_, Thin and Thick Films)

Powdered magnetite (1.50 g, 6.49 mmol) was dispersed in tris(hydroxymethyl)amino methane hydrochloride buffer (Tris-HCl, 10 mM, pH = 8.5, 150 mL). The solution was sonicated for 3 min and the pH (7.8) was rectified by the addition of concentrated NH_3_ (32% *w*/*w*) dropwise to keep the pH above 8. A solution of dopamine (DA, 2.10 g, 13.7 mmol, in 10 mL of water) was added to the suspension of Fe_3_O_4_, and stirred for 24, or 48 h, depending on whether thin or thick polydopamine (PDA) coating films were obtained. The dark brown solid, PDA@Fe_3_O_4_, was isolated by magnetic separation and washed with water. The suspension was sonicated again for 3 min, and the final washing was completed with methanol. The solid was dried for 24 h at 70 ${}^{\circ }$C (thick film 1.77 g, thin film 1.69 g).

#### 2.3.3. Synthesis of Ag NPs-PDA@Fe_3_O_4_ Catalysts (Thick Film)

PDA@Fe_3_O_4_ (400 mg for C_1_, 150 mg for C_2_ and C_3_) was dispersed in H_2_O (40 mL) and sonicated for 2 min. AgNO_3_ (150 mg, 0.88 mmol) was dissolved in the minimal amount of water and added dropwise to the Fe_3_O_4_@PDA suspension. This was then left to stir in the dark for 48 h. The dark brown solid, Ag(I)-PDA@Fe_3_O_4_, was magnetically isolated and washed once with water. The Ag+ cation in Ag(I)-PDA@Fe_3_O_4_ was reduced using the methods listed below.

- Catalyst C_1_ was prepared by adding dropwise a solution of ascorbic acid (112 mg, 0.63 mmol) in water (20 mL) to a suspension of Ag(I)-PDA@Fe_3_O_4_ (200 mg) in water (10 mL), and left to stir in the dark for 24 h.
- Catalyst C_2_ was obtained by stirring a suspension of Ag(I) NPs-PDA@Fe_3_O_4_ (200 mg) in water (20 mL) in sunlight for 24 h.
- Catalyst C_3_ was prepared by adding dropwise a solution of NaBH_4_ (80 mg, 2.11 mmol) in water (5 mL) to an aqueous suspension of Ag(I)-PDA@Fe_3_O_4_ (200 mg, 20 mL of water), and left to stir in the dark for 24 h.

#### 2.3.4. Synthesis of Au NPs-PDA@Fe_3_O_4_ Catalysts (Thick Film)

HAuCl_4_ (19.1 mg, 0.05 mmol) dissolved in H_2_O (20 mL) was added dropwise to an aqueous suspension of PDA@Fe_3_O_4_ (154 mg, 20 mL of water). This was then stirred in the dark for 48 h. The dark brown solid Au(III)-PDA@Fe_3_O_4_ was magnetically isolated and washed once with water. The Au^3+^ cation in Au(III)-PDA@Fe_3_O_4_ material was reduced through the various methods listed below.

- Catalyst C_4_ was obtained by slowly adding a solution of ascorbic acid (17.1 mg, 0.10 mmol) in water (20 mL) via a perfusion pump (0.5 mL/min) to the Au(III) NPs-PDA@Fe_3_O_4_ dispersion. This mixture was heated under stirring at 65 ${}^{\circ }$C for 60 min.
- Catalyst C_5_ was obtained only by stirring the Au(III) NPs-PDA@Fe_3_O_4_ in water (20 mL) dispersion whilst heating at 75 ${}^{\circ }$C for 1 h.
- Catalyst C_6_ was prepared by slowly adding a solution of NaBH_4_ (10.9 mg, 0.29 mmol) in water (5 mL) via a perfusion pump to the Au(III) NPs-PDA@Fe_3_O_4_ dispersion, and left to stir at 65 ${}^{\circ }$C for 60 min.

All catalysts C_1_–C_6_ were magnetically separated and washed until clear washings were observed, then lyophilized to afford dark brown solid materials (C_1_ 398 mg, C_2_ 98 mg, C_3_ 140 mg, C_4_ 157 mg, C_5_ 125 mg, C_6_ 151 mg).

#### 2.3.5. Synthesis of Cu NPs-PDA@Fe_3_O_4_ Catalyst (C_7_, Thin Film)

Solid Cu(II) acetate monohydrate (248.5 mg, 1.25 mmol) was added to a dispersion of thin film Fe_3_O_4_@PDA (300 mg) in water (50 mL) and kept under mechanical stirring for 96 h. The solid was separated with a magnet and the liquid phase was decanted. The product (Cu(II)-PDA@Fe_3_O_4_) was washed twice, once with water and once with methanol, and redispersed in methanol:water 1:1 *v*/*v* (15 mL). A solution of Bu4NBH4 (104 mg, 0.4 mmol) in water (5 mL) was added dropwise to this suspension, and this was mechanically stirred in a sonic bath for 35 min. The solid was washed with water and it was further reduced with an aqueous solution of NaBH_4_ (90 mg, 2.38 mmol, 5 mL) following the same procedure. The dark brown solid (Cu NPs-PDA@Fe_3_O_4_) was separated magnetically, washed with water and lyophilized to give a dark brown powder (264 mg).

#### 2.3.6. Synthesis of Ag NPs-PDA@Fe_3_O_4_ Catalyst (C_8_, Thin Film)

An aqueous solution of AgNO_3_ (165 mg, 0.97 mmol, 10 mL) was added to a dispersion of PDA@Fe_3_O_4_ (202 mg) in water (25 mL) and kept under mechanical agitation for 48 h. The solid was separated with a magnet and the liquid phase decanted. The product (Ag(I)-PDA@Fe_3_O_4_) was washed twice with methanol:water (1:1 *v*/*v*) and redispersed in 15 mL of the same solvent. A solution of NaBH_4_ (216 mg, 5.7 mmol) in water (10 mL) was added dropwise to this suspension, and this was mechanically stirred for 30 min. The dark brown solid (Ag NPs-PDA@Fe_3_O_4_) was separated magnetically, washed with water and lyophilized to give a dark brown powder (212.6 mg).

#### 2.3.7. Synthesis of Ag/Cu NPs-PDA@Fe_3_O_4_ Catalyst (C_9_, Thin Film)

An aqueous solution of Cu(NO_3_)_2_·3H_2_O (0.1225 g, 0.68 mmol, 5 mL) was added to a dispersion of PDA@Fe_3_O_4_ (250.4 mg) in 25 mL of H_2_O. The suspension was stirred mechanically for 96 h. The solid was separated using a magnet, the liquid phase decanted and the product (Cu(II)-PDA@Fe_3_O_4_) washed three times with water. The solid was redispersed in water (20 mL), poured into an aqueous solution of NaBH_4_ (95 mg, 2.5 mmol, 5 mL) and stirred for 40 min. The resulting product (Cu NPs-Fe_3_O_4_@PDA) was magnetically separated, rinsed with water and dried. This copper material was dispersed in water (15 mL) and solid AgNO_3_ (51.6 mg, 0.3 mmol) was added. The suspension was mechanically stirred for 10 h. The solid was magnetically separated, washed with water and lyophilized. A dark brown powder (Ag/Cu NPs-Fe_3_O_4_@PDA) was obtained (233.4 mg).

### 2.4. Procedure for the Study of Nitrophenol Reduction with UV/Vis Spectroscopy

Stock solutions of metal NPs-PDA@Fe_3_O_4_ catalysts were prepared by adding the material (10 mg) to H_2_O (10 mL) and dispersing by sonification. Dilute catalyst solutions were prepared by mixing H_2_O (1 mL), catalyst stock solution (100 μL), and NaBH_4_ (35 mg, 0.9 mmol), and leaving the solution to activate for 10 min. The solution changed color from brown to gray during activation. In a 1 cm path length UV/Vis cuvette, the nitroarene solution (2 mL, 0.7–1.4 × 10^−4^ M) was degassed with nitrogen. Then the diluted catalyst/NaBH_4_ (200 μL) was added to the nitrofenol solution, and the absorbance spectrum was recorded at room temperature between 225 to 600 nm periodically. The catalysts absorbance was measured from solutions prepared by filling a cuvette with the corresponding diluted catalyst/NaBH_4_ solution (200 μL) and water (2 mL). The corrected response, calculated by subtraction of the catalyst absorbance contribution from the reaction mixture absorbance, was analyzed as described in Section S9.2 of the Supplementary Material.

### 2.5. Catalyst Recyclability

Catalyst (8 mg) and 4-nitrophenol (10 mL, $3.9\times 10^{-2}$ M) were added to a glass tube and shaken. A 100 μL sample of the 4-nitrophenol solution (zero time sample) was extracted and transferred to a glass vials containing HCl (1 mL, 0.4 M) and diluted with H_2_O (9 mL) to stop the reaction. NaBH_4_ (400 mg, 10.57 mmol) was added to the reaction mixture and stirred at 500 rpm and 25 ${}^{\circ }$C with a thermoshaker (Hettich lab Technology). After 90 min, the mixture was magnetically separated and 100 μL of the liquid phase was extracted and treated as described above. Both samples were analyzed by HPLC running with a 50:50 H_2_O:CH_3_CN (0.1% in acetic acid) eluent at 30 ${}^{\circ }$C. The progress of the reaction was calculated by comparing the values of the chromatographic peak areas corresponding to 4-nitrophenol (retention time $t_{r}=1.58$ min) of the two samples. Finally, the catalyst was separated with a magnet and washed three times with water. The whole procedure was repeated until 4-nitrophenol conversion was below 30%.

## 3. Results and Discussion

### 3.1. Synthesis of MNPs-PDA@Fe_3_O_4_ Catalysts (M = Ag, Au)

**Figure 1:**
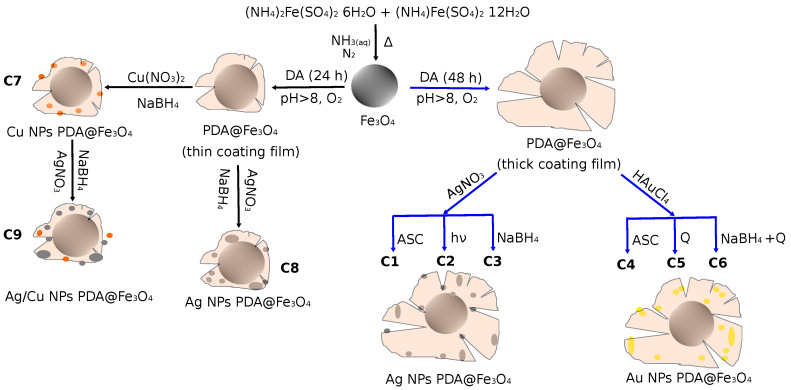
Synthesis scheme for catalysts C_1_–C_9_.

Using a two-pot method (Figure 1), nine catalysts for the hydrogenation of nitrophenol to aminophenol were developed. Firstly, a magnetic Fe_3_O_4_ core was synthesized. We chose magnetite over other iron oxides due to its higher magnetic activity. The addition of a PDA shell functionalized the inorganic support, giving a thinner or thicker layer depending on the polymerization time of the dopamine. This polymeric shell could be responsible for the chemical stability of the magnetite particles (as seen below, we did not detect changes in the XRD patterns or in the color of the sample that would suggest any change in the nature of the magnetite particles). Ag and Au nanoparticles were immobilized onto the support using different methods of reduction in the nanoparticle synthesis. For thick PDA coatings, the Ag NP catalysts were obtained by reduction with ascorbic acid, visible light or NaBH_4_, whilst the Au NP catalysts were obtained by reduction with ascorbic acid and heat, heat only or NaBH_4_, affording six different catalysts. For thin PDA coating films, the Ag and Cu NP catalysts were obtained by reduction with NaBH_4_. Finally, the mixed Ag/Cu NP catalyst was synthesized in two steps by reduction with NaBH_4_ of Ag(I) cations impregnated in a previously prepared Cu NPs PDA@Fe_3_O_4_ material.

### 3.2. Characterization of MNPs-PDA@Fe_3_O_4_ Catalysts

#### 3.2.1. Thermogravimetric Analysis

Table 1 summarizes the mass loss of materials and Figures S1 and S2 show their corresponding thermogravimetric curves. As a first observation, the amount of PDA bound to the magnetite core increases by approximately 40% when the polymerization time is doubled. The thermogravimetric curves display between 25 and 200 ${}^{\circ }$C a mass decrease by 2–4% associated to the loss of adsorbed water in the PDA pore system. Subsequently, the major mass loss associated with PDA combustion is observed (18–20% thick PDA films, 14–15% thin PDA films). The small changes in weight above 600 ${}^{\circ }$C (not shown in the figure) are due to the oxidation of the magnetite. Empirical catalyst formulae were determined from TGA (vide infra) and SEM data according to the expression ${\left({\left[{\mathrm{Fe}}_{3}O_{4}\right]}_{\frac{r}{3}}\;M\right)}_{1-w}{\left(\mathrm{PDA}\;H_{2}O\right)}_{w}$, where *w* is the percent weight change of the material and *r* is the real molar ratio Fe/M (M = Cu, Ag, Au) collected in Table 2.

#### 3.2.2. X-ray Diffraction Results

**Figure 2:**
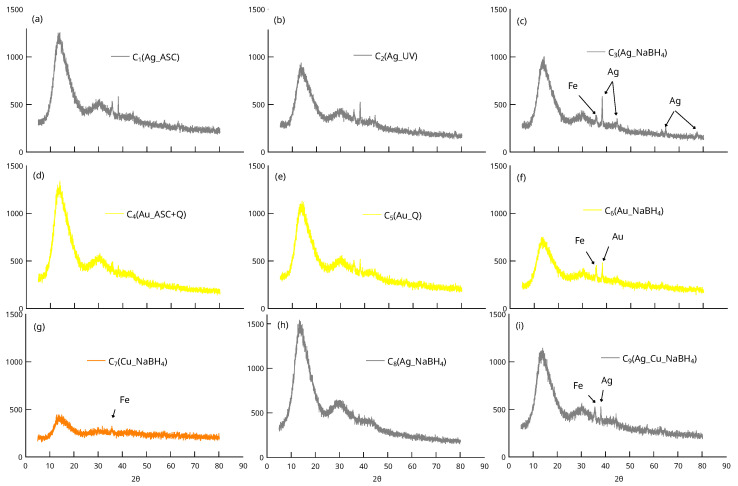
X-ray diffraction patterns of materials C_1_–C_9_ showing peaks attributable to Au and Ag crystallites. (**a**–**f**) Thick and (**g**–**i**) thin coating PDA film synthesis (Fe = Fe_3_O_4_). For simplicity, the peaks assigned to magnetite are shown only in the last column of the figure although they appear in all XRD patterns.

Figure 2 shows the X-ray diffractograms of the C_1_–C_9_ materials. As expected, in all cases a low intensity signal appears at ca. 36° (2θ) that corresponds to the more intense peak of the magnetite associated with the (311) reflection. The relatively low intensity of this signal as well as its width suggest that the magnetite cores are nanometers in size (see below). This highlights the presence of diffraction peaks that can be assigned to both Ag (2θ${}^{\circ }$ = 38.1, 44.3, 64.4 and 77.5), and Au (2θ${}^{\circ }$ = 38.3) crystallites. Their presence indicates the existence of metal aggregates with sizes greater than 5 nm in agreement with the observations made by SEM (see below). The peaks are particularly intense for the materials C_3_, C_6_ and C_9_, for which the reduction of metallic cations was carried out chemically with NaBH_4_. The figure also shows that there is only one weak diffraction peak attributed to Fe_3_O_4_ domains in the case of the Cu catalyst (C_7_). This observation suggests that even though the reduction was carried out with borohydride, the Cu atoms do not form sub-micron-sized aggregates.

#### 3.2.3. Scanning Electron Microscopy

**Figure 3:**
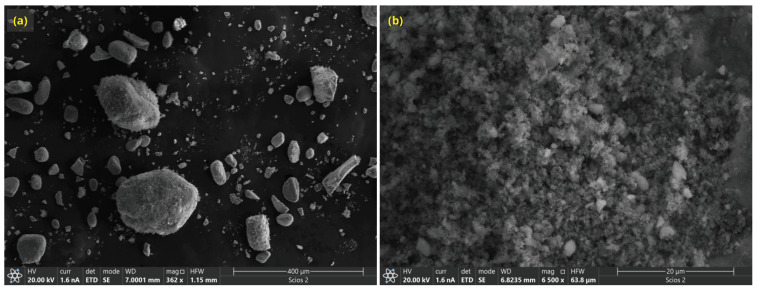
SEM micrographs of the catalyst support (Fe_3_O_4_@PDA, thin layer film) at different magnifications: (**a**) 400 μm; (**b**) 20 μm.

The texture of the catalyst support (PDA@Fe_3_O_4_) is shown in Figure 3. The synthesis resulted in micron-sized particles which are aggregates of the primary particles formed in solution during the dopamine polymerization. In this context, Figure S3 shows a TEM image of the C_8_ Ag NPs PDA@Fe_3_O_4_ catalyst, where primary nanoparticles with sizes below 20 nm are observed. In the image, PDA-coated magnetite nanoparticles (1) seem to be adhered by PDA nanoparticles (2).

**Figure 4:**
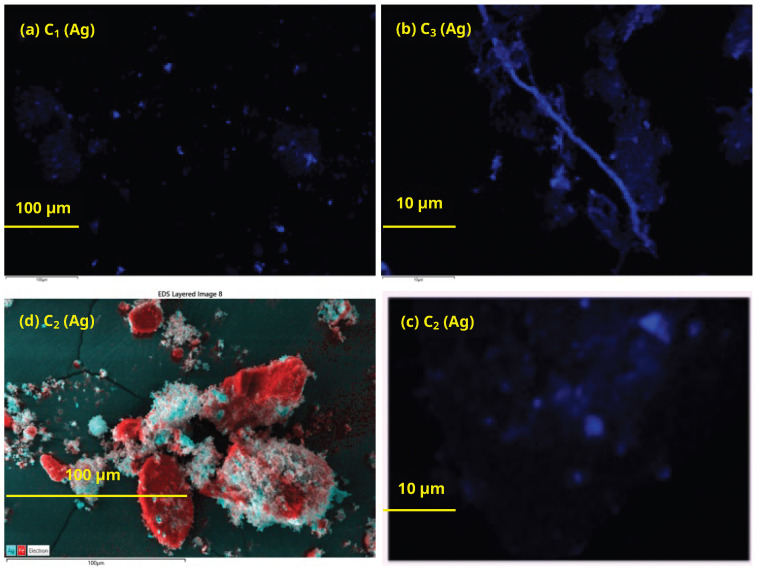
(**a**–**d**) SEM Ag mappings for materials C_1_–C_3_; (**d**) Red areas = Fe, blue areas = Ag.

Figure 4 shows Ag SEM mapping micrographs for C_1_–C_3_ materials. Material C_1_ (part (a), reduction with ascorbic acid) shows a good dispersion of Ag nanoparticles, but micron-sized crystallites are also observed (appearing as bright spots in the micrograph). This phenomenon is accentuated in material C_3_ (part (b), reduction with NaBH_4_). Here, even microwires of Ag are detected. In material C_2_ (part (c), phochemical reduction), Ag microcrystallites are also observed, but unlike material C_1_, the Ag dispersion is not homogeneous as shown in part (d), where extensive areas of the catalyst coated with Fe are observed, but there is no evidence of Ag coating.

SEM maps of the Au-containing catalysts are shown in Figure S4. In each column, the maps of Au, Fe and N are shown for each material (in the rows). In general, the dispersion of Au metal centers is better than that observed for Ag materials. The micrograph of material C_4_ (reduced with ascorbic acid) shows that the Au aggregates are relatively small and less abundant than those observed for material C_6_ (reduced with NaBH_4_), although in both cases the larger aggregates are uniformly distributed on the material. Catalyst C_5_ (reduction by PDA action by heating) shows a distribution similar to that of C_4_, although the presence of micron-sized crystallites is observed in this particular micrograph.

Figure S5 shows a SEM micrograph of Cu NPs-PDA@Fe_3_O_4_ material (a) together with Cu, Fe and N mappings. It is observed that the Cu domains must be smaller than 10 nm and uniformly distributed when compared with the Fe and N images. This observation is in agreement with the X-ray diffraction spectra, see Figure 2, where no diffraction peaks attributable to Cu are observed.

Figure S6 shows the SEM emission maps for Ag, Cu, Fe, and O of Ag/Cu NPs PDA@Fe_3_O_4_ material (C_9_). Based on the uniform dispersion of Fe (e) and O (f), the Cu domains (d) are small and uniformly distributed over the material. This is not the case for the Ag centers (c). In this case, a large size dispersion of the metal domains is observed, and also the existence of micron-sized domains irregularly distributed on the PDA surface (b). The latter observation is in agreement with the appearance of diffraction peaks in the X-ray spectra.

The Fe/M ratios calculated from the SEM-EDX peak intensities are summarized in Table 2. For the materials synthesized with a thick PDA film, the Ag-containing materials show lower Fe/M ratios than the Au-containing materials. The different amounts of Au (lower) and Ag (higher) used in the synthesis during the impregnation stage may be responsible for this phenomenon. In this respect, the low value of the Fe/Ag ratio for the C_3_ catalyst is noteworthy. As expected, the materials based on thick films of PDA show a higher silver-binding capacity than those based on thin films of PDA. Finally, the SEM-EDX microanalysis shows that the mixed Cu/Ag material (C_9_) is basically an Ag catalyst doped with a very small amount of Cu.

#### 3.2.4. Transmission Electron Microscopy and STEM

**Figure 5:**
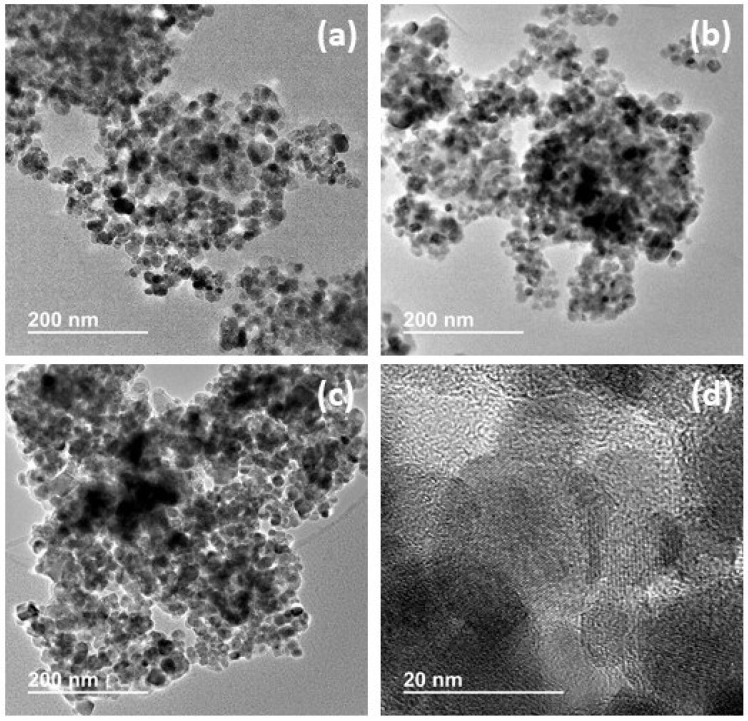
HRTEM images of catalysts (**a**) C_1_, (**b**) C_6_, (**c**) C_9_, and (**d**) C_4_.

The TEM study provides a clearer picture of the morphology of the catalysts. Representative TEM and HRTEM images are shown in Figure 5.

Regardless of the thickness of the PDA layer or the metals involved (Cu, Ag, Au), under the preparation conditions used, all catalysts have a similar morphology based on partially cohesive magnetite particles, thanks to the PDA acting as a binder. The amounts of PDA have been adjusted to ensure that not all the magnetite particles are completely embedded in a mass of PDA. In this way, we have been able to create a certain degree of textural porosity between the polymer-coated magnetite particles. This creates voids in the range of large mesopores and macropores (see below). HRTEM images show the presence of crystalline particles (magnetite according to XRD patterns) trapped in an amorphous matrix (PDA) (Figure 5d). The average size of these ordered domains is relatively small (in the 10–20 nm range). This size is consistent with the detection of XRD peaks of low intensity and high *fwhm*.

**Figure 6:**
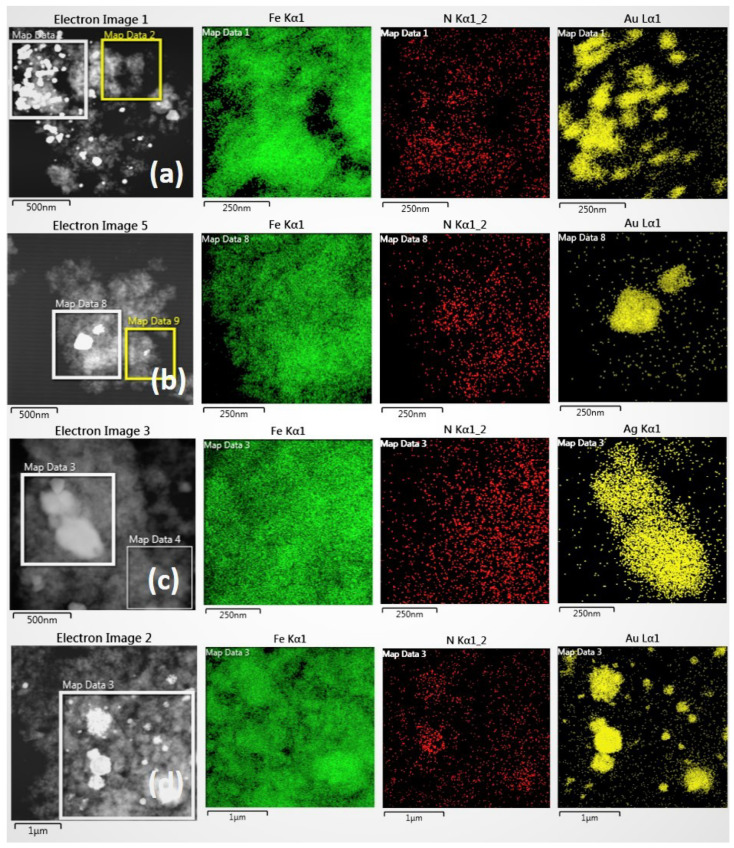
STEM-HAADF and EDX mapping of catalysts (**a**) C_4_, (**b**) C_5_, (**c**) C_3_, and (**d**) C_6_.

STEM-HAADF images combined with EDX mapping provide a detailed assessment of the distribution of noble metal nanoparticles and copper (representative images are included in Figure 6).

**Figure 7:**
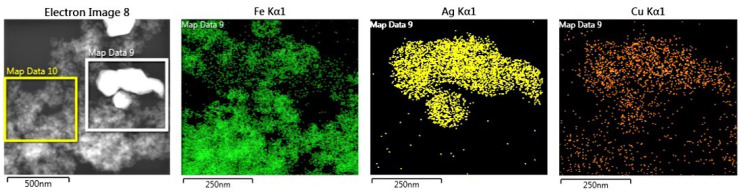
STEM-HAADF and EDX mapping of catalyst C_8_.

The trends are consistent with those detected by SEM. As more energetic noble metal reduction processes are used, the particles produced are larger and tend to form aggregates. Thus, catalysts C_1_ and C_4_ (Figure 6a) present smaller and more dispersed NPs (although with some heterogeneity in size). However, areas with different concentrations of particles are observed. Large Ag or Au particles (200–300 nm) are observed in STEM-HAADF images of catalysts C_2_ and C_5_, and especially C_3_ and C_6_, where NaBH_4_ is used as a reducing agent (Figure 6c,d). This is also observed in the case of catalysts C_8_ and C_9_ (Figure 7).

On the other hand, it should be noted that copper does not appear to be able to form nanoparticles. There is a homogeneous and similar concentration of Cu throughout the surface of the material. Possibly Cu as Cu^2+^ coordinates with OH^−^ and NH groups on the PDA surface before reduction. In the case of catalysts C_8_ and C_9_, which both contain Cu and Ag, the concentration of Cu seems to increase slightly in the vicinity of the Ag nanoparticles, but the data are inconclusive as to the existence of alloy.

#### 3.2.5. N_2_ Absorption/Desorption Isotherms

The porous nature of the catalysts is confirmed by the N_2_ adsorption–desorption isotherms. Figure S7 shows representative isotherms and Table 3 shows the values of the BET area and the pore size and volume as estimated by the BJH model. All curves are typical of unimodal pore systems with pores at the boundary between large mesopores and macropores. The pore size shows a certain heterogeneity, which is consistent with a textural type of porosity, due to the voids in the PDA matrix that imbibe the magnetite particles. Similar surface, volume, and pore size values were found for all catalysts. However, it should be noted that by using a smaller amount of PDA (thin coating), both the sizes and the pore volumes are somewhat higher than the rest of the catalysts (thick coating).

#### 3.2.6. Dynamic Light Scattering

Table 4 summarizes the results of DLS measurements on aqueous dispersions of materials prepared under the experimental conditions used in kinetic runs. The table shows the mean size (*z*), the polydispersity index (pdi), the hydrodynamic Stokes diameter (*d*), and the width of the hydrodynamic diameter distribution (σ) calculated from the correlation diagrams of the scattered photons.

Polydispersity indices were less than 0.7, suggesting that the technique was suitable for hydrodynamic diameter estimation. The pdi values indicated that all of the materials led eventually to polydisperse aqueous dispersions.

The two types of size distributions observed are shown in Figure S8. The curves were unimodal for materials C_1_, C_2_, and C_4_ and bimodal for the catalyst support and materials C_3_, C_5_, and C_6_, showing a small fraction of particles with sizes below the mean of the main distribution. For Ag-based materials, a significant increase in the hydrodynamic diameter was observed when moving from C_1_ to C_3_. The latter material presents a remarkable particle diameter (>600 nm). These observations suggested that PDA@Fe_3_O_4_ nanoparticles aggregate during the Ag+ cation reduction process. This effect was not as pronounced for Au-containing materials for which the gold load was lower. In this case, the hydrodynamic diameters were not correlated with the synthesis method and they were found to be similar to that of PDA@Fe_3_O_4_. In general, the hydrodynamic diameters of Au-based materials were smaller than those associated with Ag-containing materials.

### 3.3. Study of Catalyst Activity

In the present paper, the activity of materials in the reduction of 2-, 3- and 4-nitrophenol was investigated. The reduction of 4-nitrophenol to 4-aminophenol, using NaBH_4_ as the hydride donor, was used to compare the catalytic activity of the synthesized materials. This is a well-known reaction, extensively documented in the literature, which is widely used in catalytic studies as a reference system [25].

**Figure 8:**
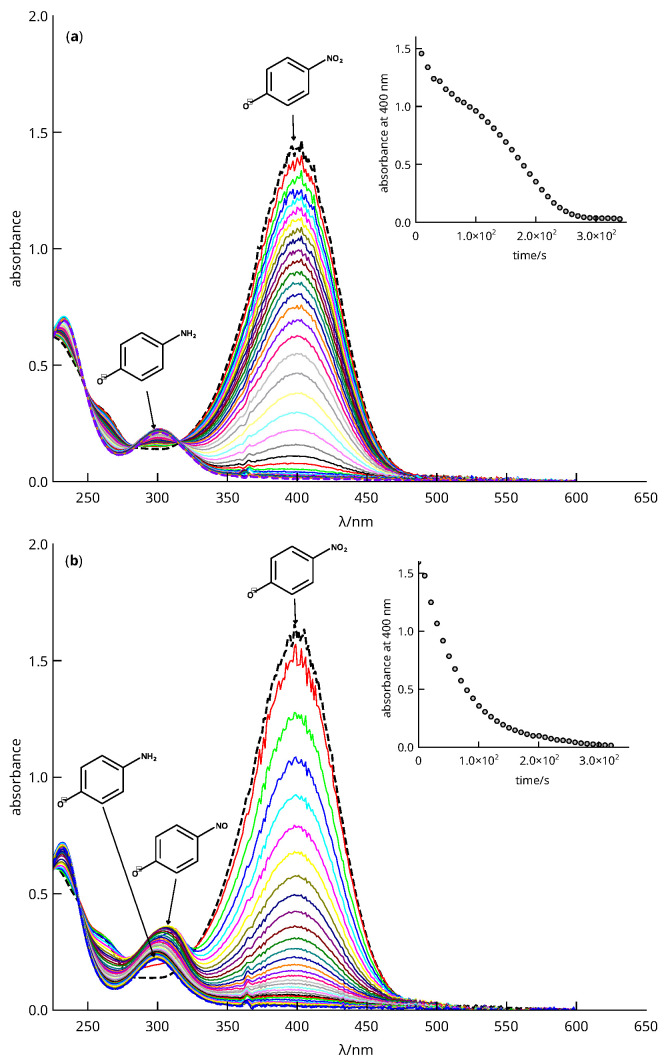
Plot of absorbance versus wavelength and time for the reduction of 4-nitrophenol ($9.2\times 10^{-5}$ M) with NaBH_4_ (0.1 M) in water at room temperature under anaerobic conditions. (**a**) Reaction catalyzed by material C_6_ (Au NPs @PDA@Fe_3_O_4_, and (**b**) by material C_4_ (Au NPs @PDA@Fe_3_O_4_. Insets show absorbance changes at 400 nm as a function of time. The absorbance of 4-nitrophenolate and 4-aminophenolate are shown as dashed spectra.

Figure 8 shows the typical absorbance variation monitored for the anaerobic reduction of 4-nitrophenol with NaBH_4_.

**Figure 9:**
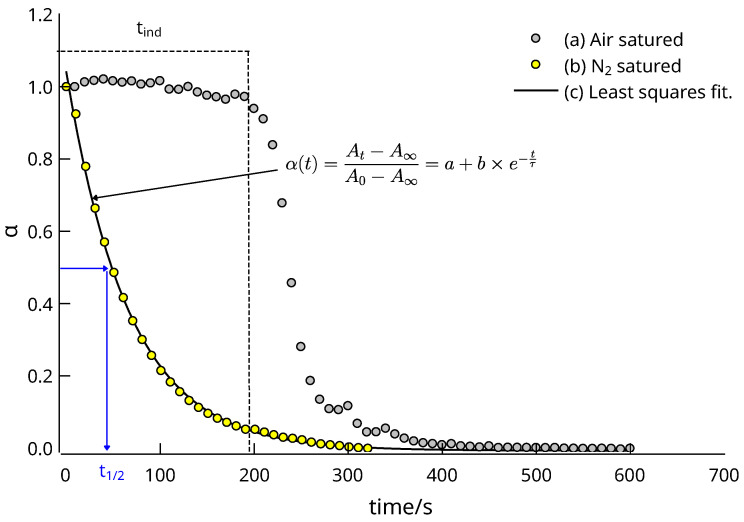
Change of α(t) calculated from the absorbance at 400 nm (water, r.t.) of a reactive mixture [4NP]_0_ = 9.2 × 10^−5^, and [NaBH_4_]_0_ = 0.1 M catalyzed by material C_4_ (Au NPs@PDA@Fe_3_O_4_). (a) Gray circles: Observed α(t) change in an air-saturated solution (induction period $t_{ind}\approx 200$ s); (b) Yellow circles: change in a N_2_-saturated solution. (c) Solid line: least-squares fit to a single exponential decay function of the data set (b). The graph shows the meaning of reaction half-time ($t_{1/2}$).

The maximum light absorption of the 4-nitrophenolate is around 400 nm, whereas the 4-aminophenolate shows a small band at 300 nm in the basic media provided by the BH4^−^ reduction. Part (a) of the figure shows a typical situation in which the nitro compound is reduced directly to the aromatic amine. The part (b), however, shows a variation in absorbance that can only be explained by the assumption of the existence of a reaction intermediate, as can be seen from the maximum absorbance at 300 nm shown in the inset. Normally, direct conversion to the aromatic amine occurs in an anaerobic environment, but in aerobic or partially oxygenated environments, autoxidation of the nitroso intermediate can occur, and this process is responsible for the appearance of an induction period [60], see Figure 9.

#### Evaluation of Activity

The catalyst activities are listed in Table 5. They are expressed through the index TOF_1/2_ calculated using Equation (1) (Section S8.1 in the Supplementary Material).

(1) $${\mathrm{TOF}}_{1/2}=\frac{1}{\left[C\right]}\frac{{\left[N\right]}_{0}\times 0.5}{t_{1/2}}$$

The TOF_1/2_ values relative to the initial concentration of sodium borohydride,

$${\mathrm{TOF}}_{1/2}^{c}={\mathrm{TOF}}_{1/2}/{\left[{\mathrm{NaBH}}_{4}\right]}_{0}$$

are also shown for the purpose of comparison with the bibliographical data. The literature suggests that the hydrogen source for nitroarene reduction can be either the hydride anion or the dihydrogen generated by the reaction of the borohydride with water. In the first case, the rate law would depend on the concentration of NaBH_4_ and comparisons should be made on ${\mathrm{TOF}}_{1/2}^{c}$ value basis. Conversely, if the reducing agent is dihydrogen, the activity will depend on the concentration of the dissolved gas, which is governed by Henry’s Law, and the index TOF_1/2_ should be used for comparison purposes. The second option is the most realistic, as previous experiments showed no noticeable variations in activity as borohydride concentration varied from 0.03 to 0.15 M (Figure S9).

**Figure 10:**
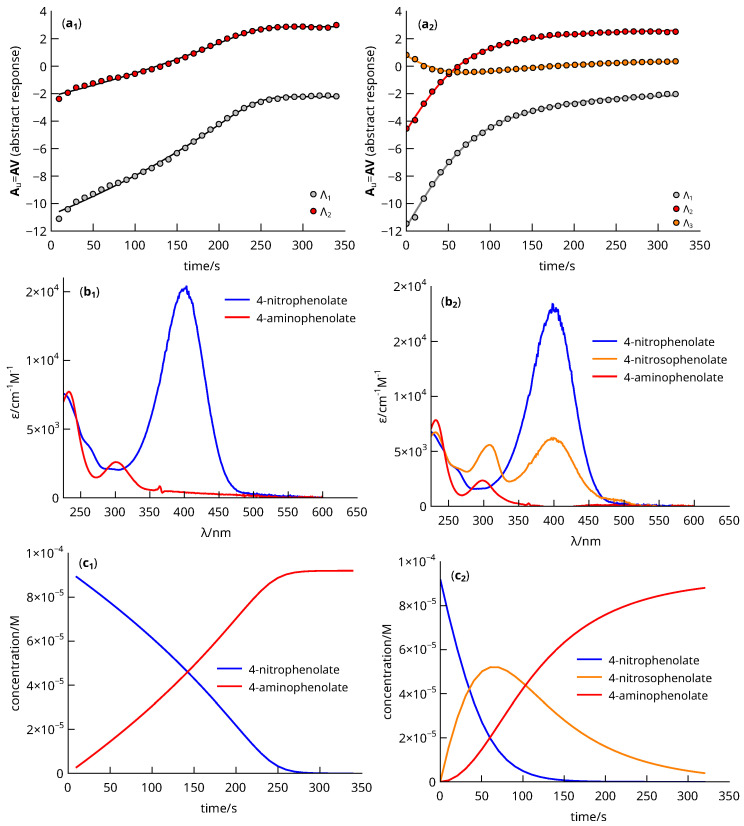
(**a1**,**a2**) Experimental (circles) and fitted (solid lines) abstract responses calculated from the factorization of the absorbance data shown in Figure 8; (**b1**,**b2**) optical density spectra and (**c1**,**c2**) concentration vs. time profiles of species.

Reaction half-times collected in Table 5 were calculated following the hard modeling analysis described in the Supplementary Material (Section S8.2) based on the rate law (mathematical model) presented therein (Section S8.1). This procedure was necessary for the comparison of all systems, as the reduction of 4-nitrophenol was via an intermediate, i.e., it was not a simple reaction, whereas the reduction of 2- and 3-nitrophenol was generally direct. Figure 10 shows the analysis results obtained when applying the procedure to the data shown in Figure 8. Part (a) shows the abstract responses ($A_{u}=\mathrm{AV}$) resulting from the factorization and removal of the array **S** (i.e., the UV–vis optical density spectra). In particular, part (a1) and (a2) display the abstract responses corresponding to the absorbance data shown in parts (a) and (b) of Figure 8. The factor analysis suggests that the absorbance is reconstructible from $n_{f}=2$ o $n_{f}=3$ absorbent species, respectively. The $n_{f}$ values are listed in Table 5.

Least-squares fitting of the abstract responses allowed calculation of the kinetic coefficients $\kappa_{1}$, $\kappa_{2}$, and *K*, as described in the Supplementary Material (Section S8.1, Equations (S11)–(S13)) and listed in Table 5. From these values, the optical density spectra (b_1_–b_2_) and species concentration (c_1_–c_2_) were calculated. The (b_1_) part of Figure 10 shows the spectra attributed to 4-nitrophenolate and 4-aminophenolate, since the conversion was direct for this system. In addition, part (b_2_) also shows a third spectrum attributed to 4-nitrosophenolate as it matches that described in the literature [61]. Finally, parts (c_1_–c_2_) show how the concentration changes with time. The half-reaction time was calculated from these curves, which allowed estimating the TOF_1/2_ values.

**Figure 11:**
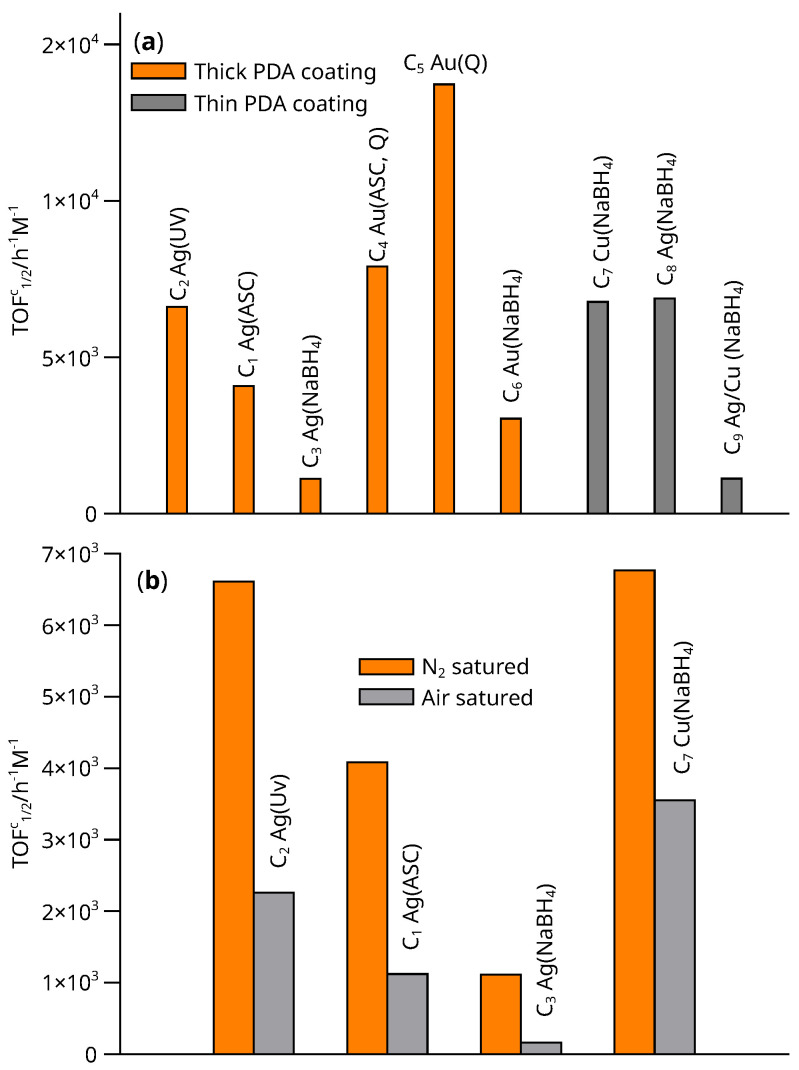
(**a**) Comparison of activity of C_1_–C_9_ catalysts in N_2_ saturated medium. (**b**) Comparison of C_1_–C_3_, C_7_ catalyst activity in N_2_ and air saturated medium. The method of synthesis is indicated in parentheses.

Figure 11a compares the activity of the materials with respect to the reduction of 4-nitrophenol according to the type of metal, the thickness of the PDA coating and the synthesis method. In general, the catalysts based on Au showed the higher activity. The maximum activity per Au atom corresponds to the material C_5_, which was prepared by reduction of Au(III) by the catechol groups after heating. The synthesis method also had a strong influence on the activity for Ag-based catalysts. Thus, the nanoparticles formed by photochemical reduction were the most active, followed by those synthesized by reduction with ascorbic acid after heating (C_5_), and finally the least active material was C_6_ prepared by chemical reduction with NaBH_4_. As a general rule, the stronger the reducing agent, the less active the synthesized catalyst will be for all metals. The figure also shows the influence of the thickness of the PDA coating on the activity. This trend suggests that, as expected, the number of active sites capable of interacting with the substrate increases significantly as the size of the noble metal NPs is reduced. Comparing Ag materials C_3_ and C_8_, with the same synthesis method but different thickness of PDA coating, a remarkable increase in activity is observed. Examination of Table 2 shows that an increase in thickness is associated with an increase in Ag fixation, as expected.

Perhaps the most important observation in terms of activity relates to the Cu material (C_7_). This is higher than that of all the thick-coated PDA catalysts, with the exception of the C_5_ material, and almost identical to its Ag counterpart (C_8_). Since Cu is a cheap biometal, the support contains only iron oxides and easily degradable organic matter, their advantages for the removal of nitroarenes in the environment are undoubted.

The literature suggests that mixed Ag/Cu catalysts are more active than those containing only one metal even with high Ag/Cu ratios. The C_9_ material was synthesized to test the hypothesis by impregnation og Cu NPs PDA@Fe_3_O_4_ with Ag^+^, in equimolar ratio with Cu, followed by reduction with NaBH_4_. However, the synthesis resulted in a material that contained a large amount of Ag (of the same order of magnitude as the C_3_ material) compared to the amount of Cu. The activity of the C_9_ material was found to be similar to that of the C_3_ catalyst. No activity improvement could be observed with the introduction of Cu.

Finally, it is worth noting that there were large activity differences in N_2_^−^ or air-saturated media, as shown in Figure 11b. For all tested catalysts, the presence of O_2_ dramatically decreased the activity due to the occurrence of an induction period (Figure 9), caused by the reaction,

$$4\text{-}\mathrm{NO}-C_{6}H_{4}\mathrm{OH}+\frac{1}{2}O_{2}⟶4\text{-}{\mathrm{NO}}_{2}-C_{6}H_{4}\mathrm{OH}$$

in which the 4-nitrosophenolate autoxidizes to 4-nitrophenolate. Over time, all the O_2_ is consumed by both the autoxidation and hydride reduction. When the oxidant is exhausted, a rapid conversion to the products occurs. The presence of O_2_ therefore slows down the removal of nitroarenes in, for example, waste water, but does not prevent it.

**Figure 12:**
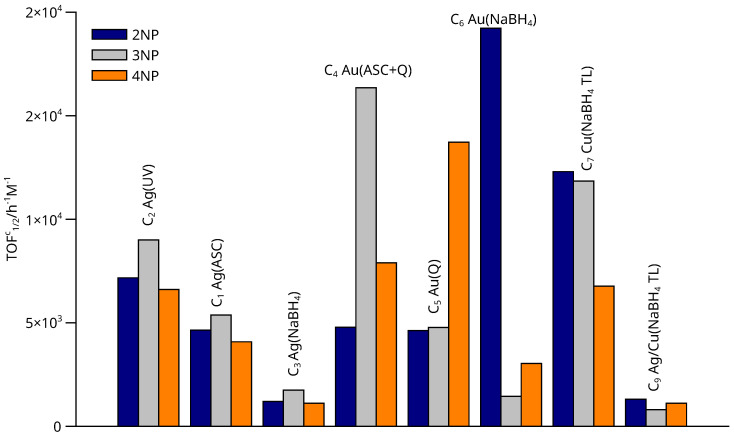
${\mathrm{TOF}}_{1/2}^{c}$ values calculated from the concentration vs. time curves calculated from the SVD analysis of absorbance data for C_1_–C_9_ catalysts in N_2_–saturated solution. The method of synthesis is indicated in parentheses.

The activity of the catalyst against 2- and 3-nitrophenol is compared in Figure 12. Materials containing Ag nanoparticles show certain regularities. These are due to the synthesis method. Reactivity was observed to decrease in the order C_2_ > C_1_ > C_3_, i.e., UV reduction produced more active catalysts and borohydride chemical reduction was less active. The activity was correlated with the hydrodinamic diameter and with the X-ray difractograms, which in turn were correlated with the size of the Ag nanoparticles anchored to the support. Thus, the larger the size of the Ag nanodomains, the lower the activity of the material. The order of activity observed was related to the substitution position of the NO_2_ group: 3-nitrophenol > 2-nitrophenol > 4-nitrophenol.

Materials based on Au nanoparticles presented a more difficult reactivity classification according to the position of the nitro group, see Figure 11a, which shows that the maximum reactivity was observed for material C_6_ (for 2-nitrophenol), followed by catalyst C_5_ (for 3-nitrophenol).

### 3.4. Catalyst Recyclability

Recycling experiments were performed in concentrated NaBH_4_ (1.06 M). This is a strongly basic medium (pH 4 > 12), in which PDA degrades slowly, leading to a progressive collapse of the material structure [62]. As a result, the metallic particles aggregate along the last cycles, and they are even released into the medium. Toward the end of the catalyst life, a decrease in mass was observed due to the combined effect of particle release and PDA dissolution causing the conversion to drop abruptly. Nevertheless, most of the prepared catalysts remained active in the reactor for more than 15 cycles in this aggressive medium. Consistently, a leaching test performed during the first recycling cycles indicated very little or no release of metal NPs. In the test, the catalyst was magnetically separated after 45 min of reaction and the liquid phase was analyzed by HPLC. The liquid was allowed to evolve for an additional 45 min and was analyzed again using the same technique. The observation of a null progression of the reaction was consistent with the absence, or very low concentration, of metallic NPs in the aqueous phase, suggesting that the catalytic process was mainly heterogeneous in nature.

**Figure 13:**
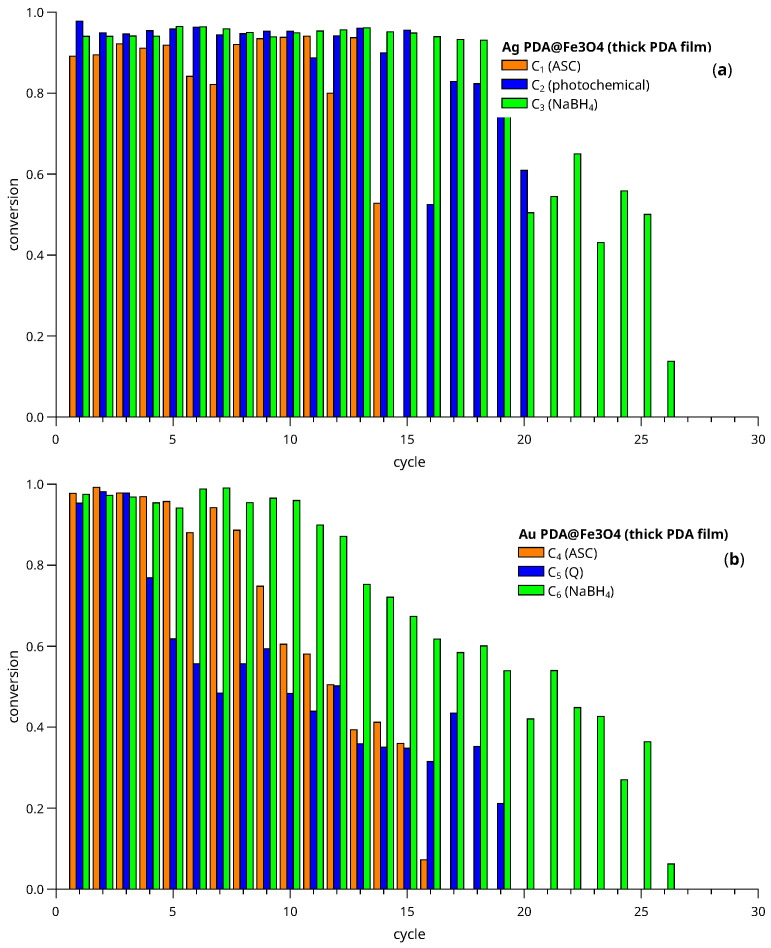
Conversion versus cycle number obtained from catalyst recycling experiments. (**a**) Catalysts C_1_–C_6_ (Ag, thick film). (**b**) Catalysts C_4_–C_6_ (Au, thick film).

**Figure 14:**
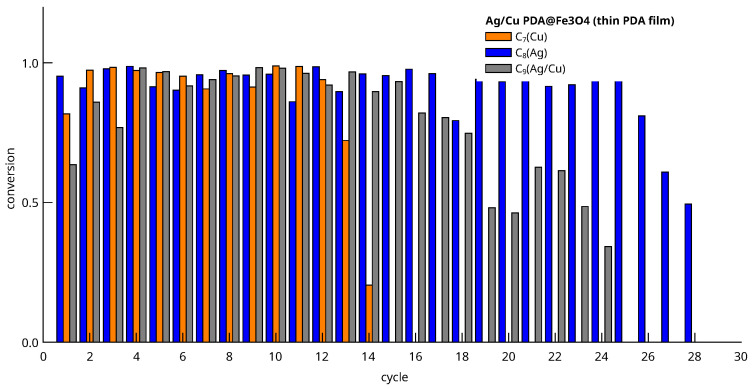
Conversion versus cycle number obtained from C_7_–C_9_ (Cu, Ag, Ag/Cu, thin film) catalyst recycling experiments.

Figure 13 and Figure 14 show the conversion of 4-nitrophenol to aniline after a given reaction time interval. The graphs display the conversion at each cycle calculated by the formula,

(2) $$x=1-\frac{A_{t}}{A_{0}}$$

where $A_{t}$ is the chromatographic peak area of 4-nitrophenol, measured from an aliquot extracted at reaction time t= (90, 120 min for C_7_), and $A_{0}$ is the area obtained after analyzing a sample just taken before adding the NaBH_4_ to the reactor.

Conversions for the catalysts obtained by depositing Ag (part a) and Au (part b) on thick PDA films are shown in Figure 13. In general, the Ag materials showed a better performance than the Au based ones. For example, C_3_ exhibited excellent conversions close to unity for 19 cycles, while the more robust Au catalyst, C_6_, presented conversion decay from cycle 12. Another interesting observation is that the most robust catalysts are those prepared using strong reductor agents as NaBH_4_ (C_3_ and C_6_), in which micrometer-sized metallic crystallites were observed. For these materials, the activity was inversely related to the robustness of the material. This loss of catalytic activity seems to be due to a significant aggregation of the gold NPs (according to STEM-HAADF data) with a subsequent decrease in the number of active sites able to interact with the substrate (Figure S11).

Figure 14 displays the results of recycling experiments for materials prepared with thin PDA films. The figure indicates that the most robust catalyst is C_8_ (Ag) in which conversion decay is observed starting at cycle 26. This is beyond the number of cycles of the best prepared thick film PDA catalyst (C_3_). For the C_8_ material, the characteristics sought after in a good catalyst, namely, durability in the reactor and high activity, are simultaneously present (Figure 11).

Figure 14 also displays the conversions of catalysts based on Cu particles (C_7_ and C_9_). In both cases, the first cycles have conversions well below unity, but conversion tends to 1 after a couple of cycles. Most probably, this phenomenon is due to a previous activation produced by the reduction of Cu particles in oxidation states I and II. The Cu-based C_7_ material has moderate robustness, as the conversion decay was observed beyond cycle 12. Therefore, we can consider this catalyst as very active but moderately robust. The C_9_ material shows a few activation cycles at the beginning, possibly due to the presence of Cu, but is more robust. There is a smooth but sustained decrease in activity over time, starting at cycle 16. The behavior is similar to a Ag thick PDA film catalyst, as the material was not very active, but being more robust than the Cu-only catalyst.

## 4. Conclusions

In this work we describe a simple, reproducible, scalable, and versatile method for the preparation of nitro-derived degradation-efficient green catalysts. These catalysts can be considered as composites made up of magnetite, PDA and metals. The benefit of a magnetic core is due to the easy separation of the catalyst after use by means of a magnetic field. The active centers are the dispersed metals (in many cases as particles). The role of the PDA (interface between the nucleus and the active centers) is crucial in the design of our materials and the advantages it provides are focused on three aspects: (1) it allows the generation of a modulable textural porosity by adjusting the amount of magnetite and PDA, (2) it acts as a binder favoring the anchoring of both metallic species in its -OH and -NH groups as well as noble metal particles, and (3) due to its chemical nature it favors the dispersibility of the catalyst in aqueous medium.

An exhaustive bibliographical work has been carried out to be able to compare the activity of our catalysts with those previously described (see Supplementary Material). From the data published in the bibliography we have determined the values of the TOF of near 300 catalysts for comparative purposes. In some isolated cases, the lack of information has made it impossible to determine the TOFs. In all cases, the catalysts prepared (C_1_, C_4_, and C_7_) are among the 25% of the most active (Figures S12–S14). This is especially notable in the case of catalyst C_7_ (Figure S12), which only contains Cu. The absence of noble metals in the C_7_ catalyst, together with the biocompatible nature of its components (Fe, Cu, and a biopolymer such as PDA) make it especially attractive due to its lower cost and eco-friendly character. Finally, it is worth noting the high reusability of the catalysts. Those containing noble metal particles support between 20 and 26 cycles and those containing exclusively Cu, at least 10 cycles without significantly losing activity. In principle, thinner PDA layers do not mean less recyclability. The higher catalytic activity of the C_8_ material, when compared to the C_3_, could, among other parameters, be related to the higher porosity of the former. In addition, we want to indicate that in the reusability studies, we used NaBH_4_ as a reducing agent, which generates a basic pH in the medium. This pH does not favor the integrity of the PDA which frays and partially degrades under these conditions. Therefore, in the case of using less energetic reducing agents, it is expected that the number of cycles, without losing activity, will increase significantly. 

## Figures and Tables

**Table 1:** Empirical catalyst formulae from TGA and EDX data.

Material	w=Δw(%)	Formula
PDA@Fe_3_O_4_ (thick film)	27.4	
C_1_	23.5	([Fe_3_O_4_]_3.2_ Ag)_0.76_(PDA H_2_O)_0.24_
C_2_	20.6	([Fe_3_O_4_]_6.3_ Ag)_0.79_(PDA H_2_O)_0.21_
C_3_	18.1	([Fe_3_O_4_]_0.55_ Ag)_0.82_(PDA H_2_O)_0.18_
C_4_	24.1	([Fe_3_O_4_]_3.3_ Au)_0.76_(PDA H_2_O)_0.24_
C_5_	18.7	([Fe_3_O_4_]_8.8_ Au)_0.81_(PDA H_2_O)_0.19_
C_6_	20.9	([Fe_3_O_4_]_12.5_ Au)_0.79_(PDA H_2_O)_0.21_
PDA@Fe_3_O_4_ (thin film)	17.7	
C_7_	13.4	([Fe_3_O_4_]_6.02_ Cu)_0.87_(PDA H_2_O)_0.13_
C_8_	14.6	([Fe_3_O_4_]_4.7_ Ag)_0.85_(PDA H_2_O)_0.15_
C_9_	17.6	([Fe_3_O_4_]_1.6_Ag Cu_0.026_)_0.82_(PDA H_2_O)_0.18_

**Table 2:** Fe/M ratios calculated from EDX microanalysis.

Catalyst	C_1_	C_2_	C_3_	C_4_	C_5_	C_6_	C_7_	C_8_	C_9_
r= Fe/M ${}^{1}$	9.6	18.9	1.6	10.0	26.5	37.4	18.1	14.0	(188.3 (Cu), 4.9 (Ag))

^1^ M = Ag (C_1_–C_3_, C_8_), M = Au (C_4_–C_6_), M = Cu (C_8_), M = Cu, Ag (C_9_).

**Table 3:** Selected physical and textural characteristics of the catalysts.

Catalyst	$C_{1}$	$C_{2}$	$C_{3}$	$C_{4}$	$C_{5}$	$C_{6}$	$C_{7}$	$C_{8}$	$C_{9}$
Surface/$m^{2}g^{-1}$	37.4	40.9	26.9	38.5	40.2	39.1	27.8	51.2	46.9
Pore Vol./${\mathrm{cm}}^{3}g^{-1}$	0.14	0.15	0.09	0.14	0.14	0.13	0.18	0.21	0.16
Pore Size/nm	26.7	25.4	29.8	21.1	20.2	25.0	35.9	31.2	35.3

**Table 4:** Hydrodynamic diameter calculated from DLS data at 25 ${}^{\circ }$ C in water.

	z/nm	pdi	$d_{1}/\mathrm{nm}$	$\sigma_{1}/\mathrm{nm}$	$d_{2}/\mathrm{nm}$	$\sigma_{2}/\mathrm{nm}$
PDA@${\mathrm{Fe}}_{3}O_{4}$ (thick film)	431	0.19	411	103.8	–	–
$C_{1}$	200	0.31	252	147.00	–	–
$C_{2}$	333	0.34	442	129.00	–	–
$C_{3}$	323	0.44	657	305.70	135.80	39.85
$C_{4}$	249	0.33	299	154.20	–	–
$C_{5}$	233	0.41	319	174.50	60.47	12.66
$C_{6}$	232	0.40	389	141.60	98.56	28.39
PDA@${\mathrm{Fe}}_{3}O_{4}$ (thin film)	210	0.20	242	90.02	–	–
$C_{7}$	470	0.41	432	158.2	–	
$C_{8}$	198	0.19	240	97.08	–	–

**Table 5:** Catalyst activity parameters evaluated using Equation (1) from the kinetic model for the reduction of 4, 3, and 2-nitrophenol with NaBH_4_ in water at r.t ${}^{†}$.

	$n_{f}$	$t_{1/2}/$s	TOF${}_{1/2}$/h^−1^	TOF ${}_{1/2}^{c}$/h^−1^M^−1^	$\kappa_{1}\times 10^{2}$	$\kappa_{2}\times 10^{2}$	$\kappa_{3}\times 10^{2}$
4-nitrophenol							
C1	3	29 ± 2	765 ± 50	7400 ± 500	3.47	1.23	2.71
C2	3	34 ± 8	680 ± 180	6600 ± 1700	4.02	0.98	5.32
C3	3	28 ± 1	115 ± 1	1100 ± 200	3.57	1.24	2.68
C4	3	22 ± 3	1400 ± 180	13,700 ± 1600	5.73	1.47	4.86
C5	3	18 ± 2	820 ± 110	7900 ± 1100	5.07	1.83	1.78
C6	2	144 ± 25	310 ± 50	3000 ± 500	7.80	–	2.68
C7	3	26 ± 3	700 ± 70	6800 ± 700	5.51	0.98	43.6
C8	3	22 ± 3	700 ± 100	6900 ± 900	6.61	1.63	6.64
C9	2	55 ± 7	116 ± 15	1120 ± 140	4.82	–	14.0
3-nitrophenol							
C1	2	23 ± 4	550 ± 110	5400 ± 1000	3.09	–	0.028
C2	2	25 ± 4	930 ± 200	9000 ± 1800	3.26	–	1.09
C3	2	18 ± 2	180 ± 25	1750.6 ± 220	4.11	–	0.63
C4	3	9 ± 1	1700 ± 250	1600 ± 2300	12.4	1.96	1.94
C5	2	132 ± 8	425 ± 15	4100 ± 150	1.05	–	0.90
C6	2	300 ± 30	150 ± 15	1450 ± 130	0.23	–	0.00
C7	2	15.2 ± 0.5	1225 ± 40	11,800 ± 400	4.73	–	0.32
C9	2	80 ± 15	140 ± 5	1300 ± 50	1.89	–	2.33
2-nitrophenol							
C1	2	27 ± 4	480 ± 70	4650 ± 750	2.86	–	0.002
C2	2	31 ± 1	740 ± 20	7170 ± 140	2.92	–	2.11
C3	2	28 ± 6	125 ± 25	1200 ± 250	3.02	–	1.28
C4	2	40 ± 9	500 ± 125	4800 ± 1200	1.94	–	0.56
C5	2	69 ± 15	480 ± 100	4600 ± 900	1.35	–	2.16
C6	2	25 ± 8	2000 ± 600	19,000 ± 6000	2.61	–	0.00
C7	2	15 ± 1	1270 ± 100	12,300 ± 900	5.05	–	0.58
C9	2	47 ± 2	140 ± 5	1300 ± 50	1.89	–	2.33

^†^ The meaning of $\kappa_{1}$, $\kappa_{1}$ and *K* is given by Equations (11)–(13) in the Supplementary Material (Section S8.2).

## Supplementary Materials

The following supporting information can be downloaded at: https://www.mdpi.com/article/10.3390/nano13152162/s1. Figure S1: TGA of catalysts C_7_–C_9_; Figure S2: TGA of catalysts C_1_–C_6_; Figure S3: TEM micrographs of Ag NPs-PDA@Fe_3_O_4_ (C_8_); Figure S4: SEM mappings of Au NPs-PDA@Fe_3_O_4_ (C_4_-C_5_); Figure S5: SEM mappings of Cu NPs-PDA@Fe_3_O_4_ (C_7_); Figure S6: SEM mappings of Ag/Cu NPs-PDA@Fe_3_O_4_ (C_9_); Figure S7: N_2_ adsorption-desorption isotherms; Figure S8: DLS intensity distribution curves of catalysts C_1_ and C_3_; Figure S9: Dependence of TOF_1/2_ on NaBH_4_ initial concentration; Figure S10: The Haber mechanism; Figure S11: STEM-HAADF and EDX mapping of recycled C_6_ catalyst; Figure S12: bibliographic analysis of Cu-containing catalysts; Figure S13: bibliographic analysis of Ag-containing catalysts; Figure S14: bibliographic analysis of Au-containing catalysts; Table S1: TOF bibliographic values for Cu-containing catalysts; Table S2: TOF bibliographic values for Ag-containing catalysts; Table S3: TOF bibliographic values for Au-containing catalysts; Section S1: TGA additional results; Section S2: TEM additional results; Section S3: SEM additional results; Section S4: N_2_ absortion/desortion isotherms; Section S5: DLS additional results; Section S6: Dependence of TOF_1/2_ on [NaBH_4_]0; Section S7: rate law; Section S8: Data analysis; Section S9: Recycling experiments; Section S10: TOF_1/2_ values for selected catalysts calculated from bibliographic data.
