# Supplementary material for: Synthesis and Catalytic Activity for 2, 3, and 4-Nitrophenol Reduction of Green Catalysts Based on Cu, Ag and Au Nanoparticles Deposited on Polydopamine-Magnetite Porous Supports
Source: Nanomaterials (Basel). 2023 Jul 25;13(15):2162. doi: 10.3390/nano13152162 (PMC10421209; doi:10.3390/nano13152162)
Supplement: Supplementary file 1 [file nanomaterials-13-02162-s001.zip › nanomaterials-2521388-supplementary.pdf]

Supplementary Materials: Synthesis and Catalytic Activity for 2, 3, and 4-Nitrophenol reduction of Green Catalysts Based on Cu, Ag and Au Nanoparticles Deposited on Polydopamine-Magnetite Porous Supports

Helen K. Brown[1], Jamal El Haskouri [1], María D. Marcos [2], José Vicente Ros-Lis [3], Pedro Amorós [1], M. Ángeles Úbeda Picot [4,*], Francisco Pérez-Pla [1,*]

1. TGA additional results

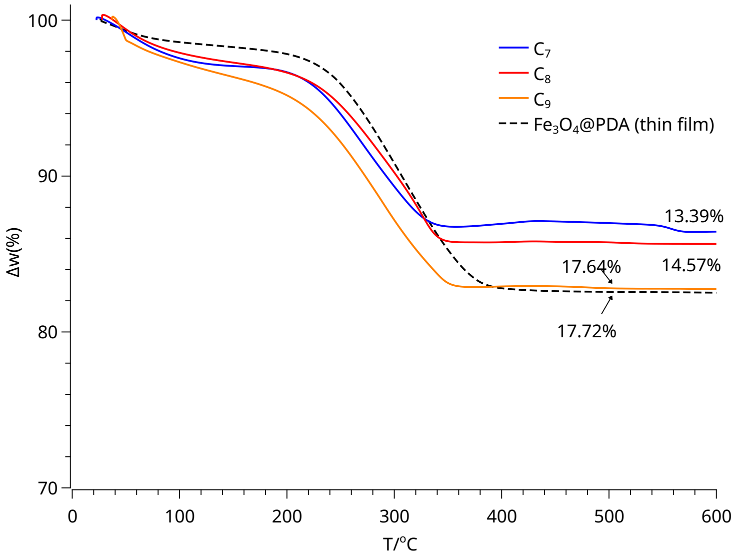

Figure S1. Mass loss of the materials C_7-C_9 (thin film) as a function of temperature; The mass loss of the support ($PDAFe_3O_4$) is shown (dashed line).

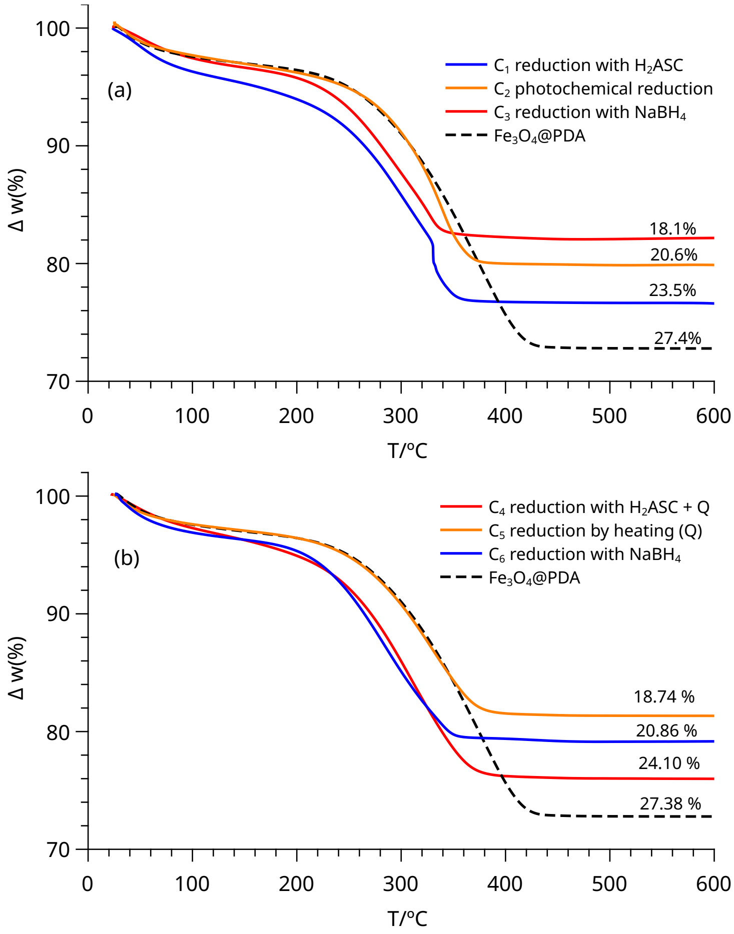

Figure S2. Mass loss of the materials C_1-C_6 (thick film) as a function of temperature; (a) Materials with Ag nanoparticles (C_1-C_3); (b) Materials with Au nanoparticles (C_4-C_6); The mass loss of the support is shown (dashed line).

2. TEM additional results

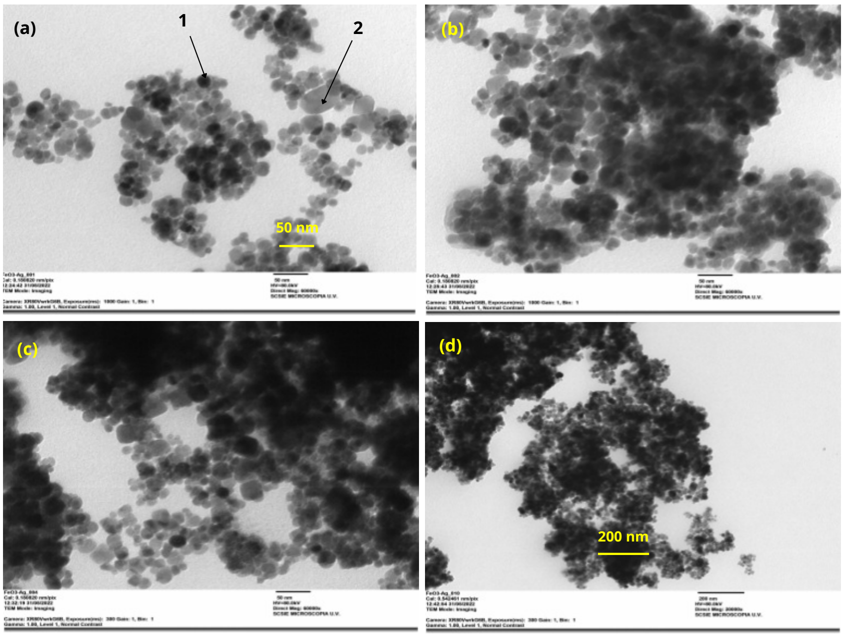

Figure S3. TEM micrograph of Ag NPs-PDA@Fe_3O_4 (C_8). (a) The arrows point at particles of magnetite coated with PDA (1), and pure PDA nanoparticles (2); (b) and (c) different catalyst areas at 50 nm scale; (d) Micrograph at 200 nm scale.

3. SEM additional results

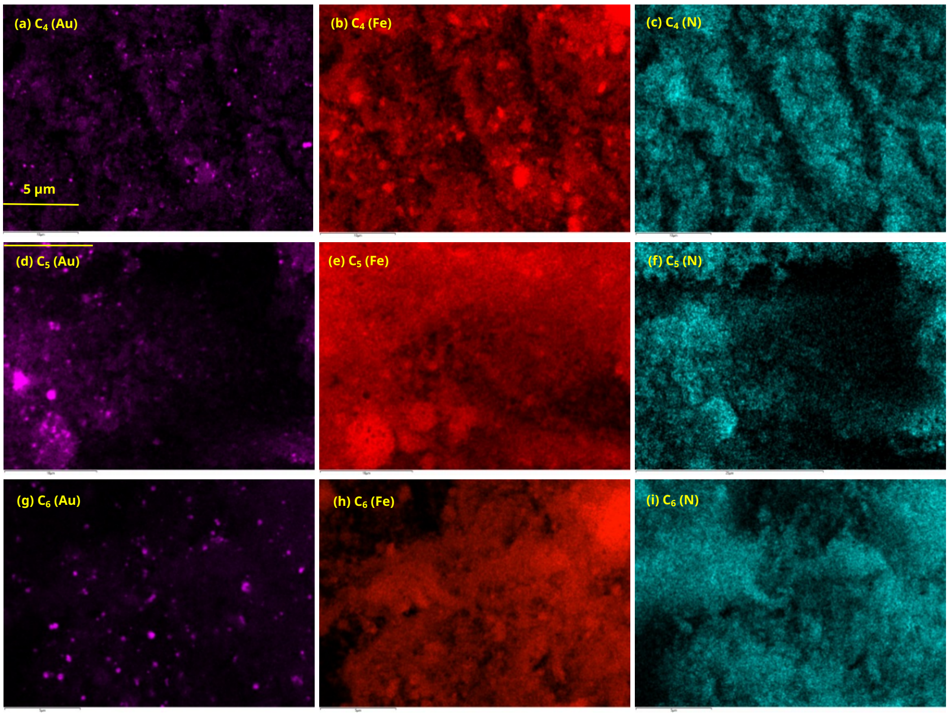

Figure S4. SEM Au, Fe, and N mappings for C_4–C_6 materials.

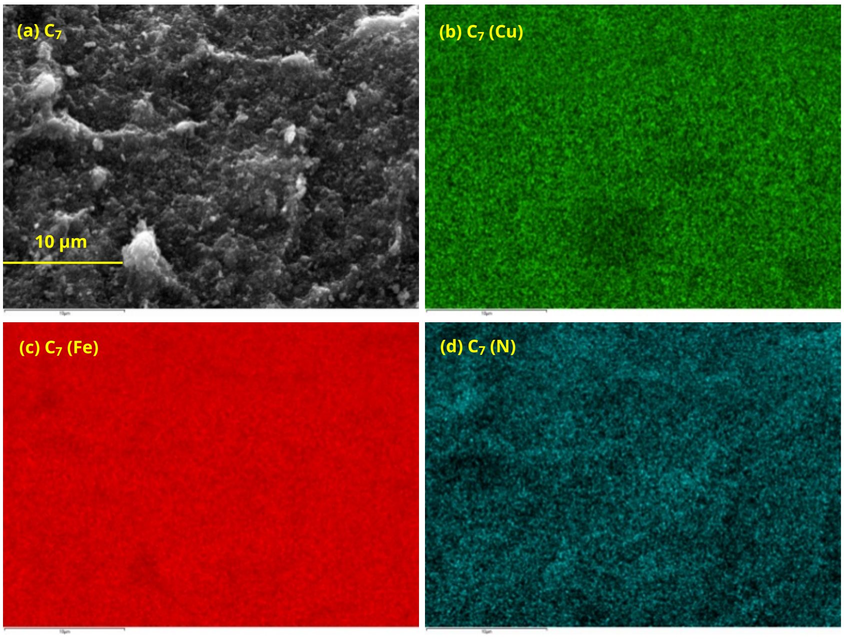

Figure S5. (a) SEM micrograph of material Cu NPs-PDA@Fe_3O_4 (C_7); (b-d) Mappings of cu, Fe, Cu, and N respectively.

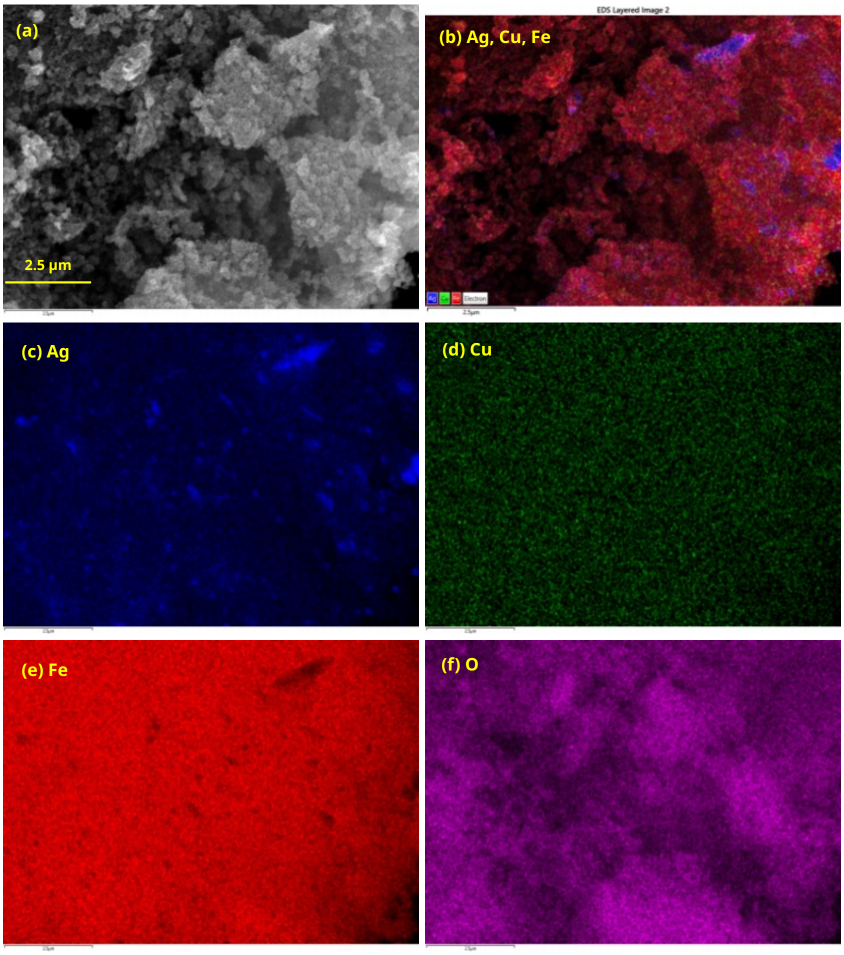

Figure S6. (a) SEM micrograph of material Ag/Cu NPs-PDA@Fe_3O_4 (C_9); (b) Combined mappings of Ag and Cu; (c-f) Mappings of Ag, Cu, Fe and O respectively.

4. N_2 absortion/desortion isotherms

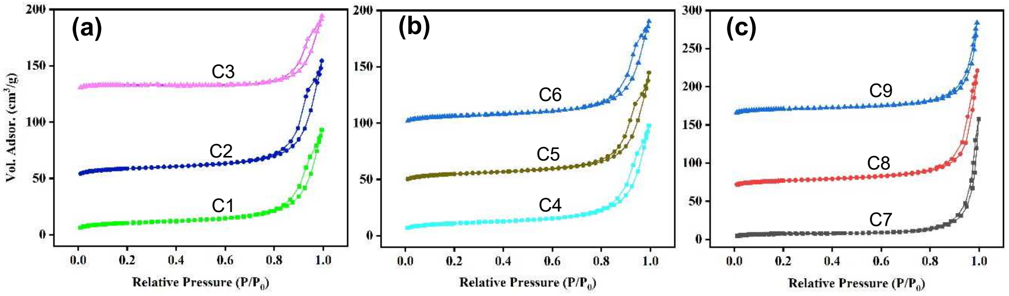

Figure S7. N_2 adsorption-desorption isotherms of catalysts.

5. DLS additional results

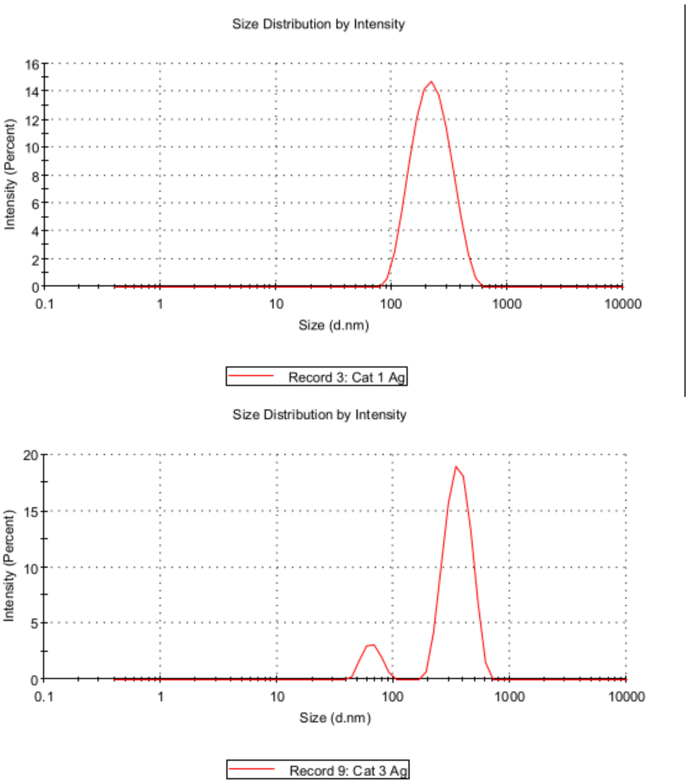

Figure S8. DLS intensity distribution. Upper part: unimodal distribution observed for material C_1; Lower part: bimodal distribution observed for material C_3.

6. Dependence of $TOF_{1/2}$ on $[NaBH_4]_0$

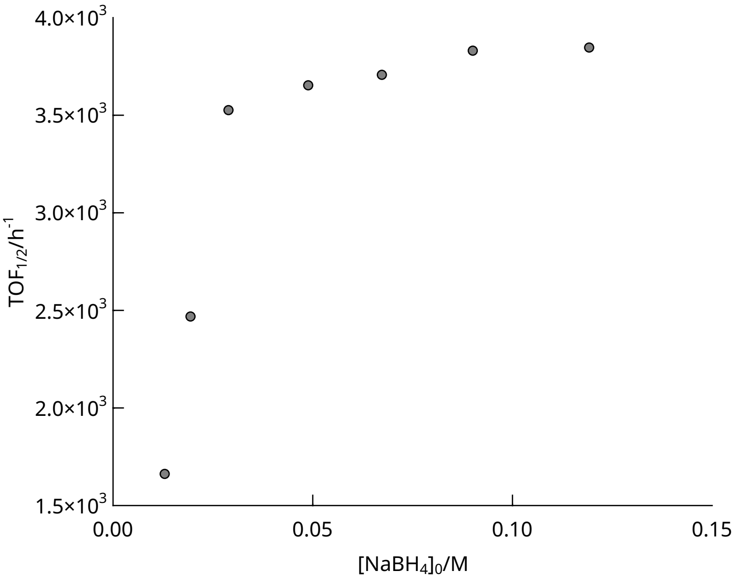

Figure S9. Variation of $TOF_{1/2}$ for 4-nitrophenol reduction in aerobic medium with initial $NaBH_4$ concentration catalyzed by Cu NPs $PDA@Fe_3O_4$ (C_7).

7. Rate law

7.1. The Haber mechanism

The reduction of 4-nitrophenol follows the general Haber [1–3] mechanism, which consists of two pathways, namely the direct and the condensation pathways, shown as (**a**) and (**b**) in Figure S10. In the direct pathway, the nitro group (NO_2) reduces to amino by

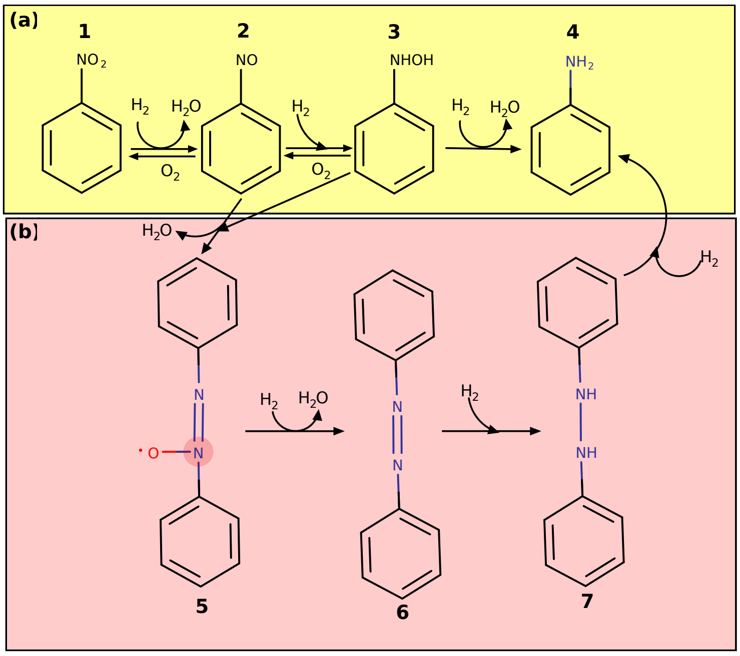

Figure S10. (**a**) The direct, and (**b**) condensation pathways of Haber mechanism for nitroarene reduction particularized for nitrobenzene; Compounds involved: **1** nitrobenzene, **2** nitrosobenzene, **3** phenylhidoxylamine, **4** aminobenzene, **5** azoxy derivative: ((1,2-diphenyl-1λ_4-diazen-1-yl)oxidanyl), **6** azobenzene, and **7** hydrazobenzene.

two consecutive steps through the nitroso (NO), and hydroxylamino (NHOH) intermediate products,

$$PhNO_2 + H_2 \xrightarrow{r_1} PhNO + H_2O$$
$$PhNO + H_2 \xrightarrow{r_2} PhNHOH$$
$$PhNHOH + H_2 \xrightarrow{r_3} PhNH_2 + H_2O$$

Normally, the reaction proceeds by the direct pathway at low nitroarene concentrations in anaerobic media. Condensation occurs more extensively in oxygenated reactors at high nitroarene concentrations. Both the nitrous and hydroxylamino species revert to their parent compounds by autoxidation, as shown in Figure S10. This is the reason why the condensation pathway is activated by the presence of oxygen. Under aerobic conditions, autoxidation produces a sufficient amount of nitro derivative, which condenses with the hydroxylamino derivative to form an azoxy compound. The latter is further reduced in several steps to the hydrazo species which are finally reduced to the aromatic amine. Direct reduction was the predominant pathway under the experimental conditions in which activity was measured. In the following, A_1, A_2, A_3, and A_4 stand for the nitro, nitroso, hydroxylamino, and amino derivatives respectively. θ denotes the total number of active centers, θ_L the number of unoccupied centers, θ_A those occupied by nitroarene molecules, and θ_H those occupied by H atoms. Reaction begins with the generation of H_2 due to the reduction of water by the $BH_4{}^-$ anion in the presence of the catalyst [4,5],

$$BH_4{}^- + 4\,H_2O \xrightarrow{cat.} B(OH)_4{}^- + 4\,H_2$$

Hydrogen, a poorly water-soluble gas, saturates the solvent so its concentration is considered constant. Subsequently, the nitroarene and hydrogen adsorb onto the metal sites. Hydrogen adsorbs dissociatively to the metal nanoparticles, forming H radicals (θ_H),

$$H_2 + 2\,\theta_L \xrightleftharpoons{K_H} 2\,\theta_H \tag{1}$$
$$A_1 + \theta_L \xrightleftharpoons{K_A} 2\,\theta_A \tag{2}$$

The nitroso compound (A_2) is then formed by the reaction of the adsorbed species,

$$\theta_A + 2\,\theta_H \xrightarrow{k_1} A_2 + 3\,\theta_L + H_2O \tag{3}$$

The nitrosoderivative now reacts with the adsorbed H-atoms to form the hydroxylamino compound (A_3). This in turn reacts with the H-atoms to form the aromatic amine (A_4),

$$A_2 + 2\,\theta_H \xrightarrow{k_2} A_3 + 2\,\theta_L \tag{4}$$
$$A_3 + 2\,\theta_L \xrightarrow{k_3} A_4 + 2\,\theta_L + 2\,H_2O \tag{5}$$

Firstly, a mathematical expression is derived for the rates of the reactions 3-5. For this task, we assume that the reaction 3 follows the Langmuir-Hinshelwood mechanism [6], i.e. the reactants (H_2, A_1) adsorb onto the catalyst through an equilibrium process, and the

adsorbed species react with each other to form the product. In considering equilibria 1 and 2, the following relations must be satisfied,

$$K_A = \frac{\theta_A}{\theta_L[\mathrm{A}_2]} \rightarrow \theta_A = K_A[\mathrm{A}_1]\theta_L \tag{6}$$

$$K_H = \left(\frac{\theta_H}{\theta_L}\right)\frac{1}{[\mathrm{H}_2]} \rightarrow \theta_H = \sqrt{K_H[\mathrm{H}_2]}\theta_L \tag{7}$$

$$\theta = \theta_A + \theta_H + \theta_L \tag{8}$$

The solution of the system of linear equations 6-8 in θ_A and θ_H yields the following solutions,

$$\theta_A = \theta\frac{K_A[\mathrm{A}_1]}{1+\sqrt{K_H[\mathrm{H}_2]}+K_A[\mathrm{A}_1]} \tag{9}$$

$$\theta_H = \theta\frac{\sqrt{K_H[\mathrm{H}_2]}}{1+\sqrt{K_H[\mathrm{H}_2]}+K_A[\mathrm{A}_1]} \tag{10}$$

Once this information has been obtained, the rate of the reactions 3-5 is calculated,

$$r_1 = k_1\theta_A\theta_H = k_1\theta^2\frac{\dfrac{K_A\sqrt{K_H[\mathrm{H}_2]}}{\left[1+\sqrt{K_H[\mathrm{H}_2]}\right]^2}[\mathrm{A}_1]}{\left[1+\dfrac{K_A[\mathrm{A}_1]}{1+\sqrt{K_H[\mathrm{H}_2]}}\right]^2} = \frac{\kappa_1[\mathrm{A}_1]}{(1+K[\mathrm{A}_1])^2} \tag{11}$$

$$r_2 = k_2\theta_H[\mathrm{A}_2] = k_2\theta\frac{\dfrac{\sqrt{K_H[\mathrm{H}_2]}}{1+\sqrt{K_H[\mathrm{H}_2]}}[\mathrm{A}_2]}{1+\dfrac{K_A[\mathrm{A}_1]}{1+\sqrt{K_H[\mathrm{H}_2]}}} = \frac{\kappa_1 2[\mathrm{A}_2]}{1+K[\mathrm{A}_1]} \tag{12}$$

$$r_3 = k_3\theta_H[\mathrm{A}_3] = k_2\theta\frac{\dfrac{\sqrt{K_H[\mathrm{H}_2]}}{1+\sqrt{K_H[\mathrm{H}_2]}}[\mathrm{A}_3]}{1+\dfrac{K_A[\mathrm{A}_1]}{1+\sqrt{K_H[\mathrm{H}_2]}}} = \frac{\kappa_3[\mathrm{A}_3]}{1+K[\mathrm{A}_1]} \tag{13}$$

where the meaning of κ_i and K is inferred from the Eqs. 11-13.It is worth noting that in deriving the rate equations it was assumed that the nitroarene reduction follows a rate of $r_1 = k_1\theta_A\theta_H$ instead of the expected $r_1 = k_1\theta_A\theta_H^2$. This simplification takes into account the fact that the reduction of the nitro group produced by two adsorbed H radicals takes place in succession at different rates, one of which is rate-limiting. On the other hand, it was also considered that the compounds A_2 and A_3 are easily desorbed and interact with the adsorbed H radicals directly from the liquid phase,that is, the reduction of A_2 and A_3 species follow the Eley-Rideal mechanism [7].

The rate law can be written once the equations governing the rate of the reactions are known,

$$\frac{d\mathbf{c}}{dt} = \mathbf{Nr} \rightarrow \frac{d}{dt}\begin{pmatrix}[\mathrm{A}_1]\\ [\mathrm{A}_2]\\ [\mathrm{A}_3]\\ [\mathrm{A}_4]\end{pmatrix} = \begin{pmatrix}-1 & 0 & 0\\ 1 & -1 & 0\\ 0 & 1 & -1\\ 0 & 0 & 1\end{pmatrix}\begin{pmatrix}r_1\\ r_2\\ r_3\end{pmatrix} \tag{14}$$

In Eq. 14, **N** stands for the stoichiometric coefficients array (stoichiometry of reactions 3-5 arranged by columns), and **r** is an array collecting the reaction rates in Eqs. 11-13.

8. Data analysis

8.1. Soft-modeling method to calculate the catalytic activity

Figure **??** shows a typical exponential decay of the remaining limiting reactant fraction ($\alpha(t)$. This variable is defined by Eq. 15,

$$\alpha(t) = \frac{[\mathrm{N}]}{[\mathrm{N}]_0} = \frac{A(t) - A_\infty}{A_0 - A_\infty} \tag{15}$$

where [N] stands for the nitroarene concentration (i.e. the limiting reactant). The α values allow easy determination of the material's catalytic activity, but only for simple reactions (i.e., those in which the reactants convert to products without observable intermediates). First, we define the instantaneous turn over frequency (TOF(t)) as

$$\mathrm{TOF}(t) = -\frac{1}{[\mathrm{C}]}\frac{d[\mathrm{N}]}{dt} \tag{16}$$

where [C] represents the catalyst concentration. This quantity is a continuous function of time. Therefore, the value of this variable must be specified in order to compare the activities of different materials. Activity is usually more conveniently expressed as a time average,

$$\mathrm{TOF} = -\frac{1}{[\mathrm{C}]}\frac{\Delta[\mathrm{N}]}{\Delta t} \tag{17}$$

where Δ[N] is the consumption of the limiting reagent over the time interval, Δt. Again, either Δ[N] or Δt must be specified in order for the activities to be compared correctly. There are some reference times for the calculation of the TOF value.The first is at the very beginning of the reaction (that is, at $t = 0$). In the case of ordinary reactions, this is the maximum value of the TOF at which the reaction will proceed. It has the disadvantage of not being applicable to reactions that have an induction period.

Another reference widely used in the bibliography is the determination of the time (t_r) at which a relative consumption (r) of the limiting reagent occurs. The TOF_r is then defined by eq. 18,

$$\mathrm{TOF}_r = \frac{1}{[\mathrm{C}]}\frac{[\mathrm{N}]_0 \times r}{t_r} \tag{18}$$

However, rarely TOF values with the same consumption are specified, making it difficult to compare the reported activities. In fact, TOF_r is often reported, but not r or t_r.

In order to avoid these drawbacks, the half-reaction time ($t_{1/2}$) is used as a reference in this paper. The reduction of 4-nitrophenol often follows a first order kinetics, for which the rate constant (k) is usually reported. In this case, Eq. 18 becomes Eq. 19 when r is set to the value $r = 1/2$,

$$\mathrm{TOF}_{1/2} = \frac{1}{[\mathrm{C}]}\frac{[\mathrm{N}]_0 \times 0.5}{t_{1/2}} \tag{19}$$

Substituting $t_{1/2}$ into Eq. 19 leads to Eq. 20, where k is the first-order rate constant, since for a first-order reaction it holds that $t_{1/2} = \ln(2)/k$,

$$\mathrm{TOF}_{1/2} = \frac{1}{[\mathrm{C}]}\frac{[\mathrm{N}]_0 \times 0.5 \times k}{\ln(2)} \tag{20}$$

The applicability of Eq. 19 depends on estimating the value of $t_{1/2}$. For simple kinetics, the α values are calculated from the experimental absorbance data using Eq. 15 and the value of $t_{1/2}$ is linearly interpolated using $\alpha = 0.5$. It is worth noting that it is not necessary to know the rate law to calculate the time for these systems, and for this reason, the methodology is known as *soft-modeling*.

8.2. Hard-modeling method to calculate the catalytic activity

For complex reactions, half-lives are calculated by linear interpolation of the [N]/2 value from the 4-nitrophenol concentration versus time curve calculated by the hard modeling method described in this section. The basis of this data analysis is the Lambert-Beer law (Eq. 21),

$$\mathbf{A}(n_t, n_r) = \mathbf{C}(n_t, n_s)\mathbf{S}(n_s, n_r) \tag{21}$$

In Eq. 21, **A** is the array of absorbances, and **C** and **S** are arrays that store the molar concentration and the optical density coefficients of the species. The elements of matrix **C** are calculated by numerical integration of the ordinary differential equation system given by Eq. 14. Dimensions n_t, n_r, and n_s stand for the number of measurements (i.e. number of times at which the absorbance has been measured), the number of observation channels (i.e. the number of wavelengths at which absorbance has been measured simultaneously by the diode-array detector), and the number of UV-vis absorbing species respectively. As a guideline, the absorbance was measured in 375 channels, and from 30 to 100 times, so each array consists of 11250 to 37500 elements.

The purpose of the hard-modeling analysis is the determination of the kinetic coefficients κ_i and K by means of the least-squares method. Regression analysis consists in minimizing the values of the residuals (**R**) related to the above mentioned variables. The residuals are defined by Eq. 22,

$$\mathbf{R}(\kappa, K, \mathbf{S}) = \mathbf{A} - \mathbf{C}(\kappa, K)\mathbf{S} \tag{22}$$

where **A** is the experimental absorbance array, and the product **CS** is the calculated absorbance estimated from the rate law. The array **C** is calculated by numerical integration of the differential Eq. 14. Note that residuals are a nonlinear function of kinetic coefficients and linearly dependent on optical density coefficients. Therefore, the elements of array **S** are often called *linear parameters*.

The number of variables for which the residuals need to be minimized can be overwhelming. For example, if we realize the system consists of 3 absorbing species (e.g. the nitroarene, nitrosoarene, and aromatic amine), and the absorbance of the reaction mixture is measured at 375 wavelengths, the residuals will depend on 1125 optical density coefficients, to which 2 values of κ, and a value of K have to be added, making 1128 variables on the whole. Obviously, the number of optical density coefficients is too large to perform the minimization successfully. However, the catalyst activity, the ultimate goal of the calculation, only depends on 3 variables, namely κ_1, κ_2, and K.

In order to solve this issue, the array **A** is factored [8] using the Singular Value Decomposition (SVD) technique [9], which allows for reduction of the n_r value, and after factorization, array **S** is removed from Eq. 22. The algorithms that perform both tasks are called Multivariate Curve Resolution (MCR) methods. It is important to highlight the term "resolution", since the result of array **A** analysis makes possible to find out the change with time of concentration of species involved in the mechanism, together with their optical density spectra. Below, the two steps of the procedure, factorization and linear parameter removal, are briefly explained.

As indicated above, the factorization is carried out using the SVD algorithm. In principle, any two-dimensional array can be written as the matrix product shown in Eq. 23, where (^{T}) denotes the transpose matrix operator,

$$\mathbf{A}(n_t, n_r) = \mathbf{U}(n_t, n_r)\mathbf{\Lambda}(n_r, n_r)\mathbf{V}^T(n_r, n_r) \tag{23}$$

The **U** and **V** arrays have the property of being orthonormal (i.e. $\mathbf{UU}^T = \mathbf{1}$, $\mathbf{VV}^T = \mathbf{1}$), and they are known as the *abstract concentration* and *spectra* arrays respectively. The diagonal $\mathbf{\Lambda}$ array contains the singular values of **A**.

The analysis of array $\mathbf{\Lambda}$ reveals that the first n_f elements of the main diagonal have very high values, the rest of them being very small. In fact, they are not null due to the

experimental uncertainty of absorbance measurements. This means that Eq. 23 can be rewritten as,

$$\mathbf{A}(n_t, n_f) = \mathbf{U}(n_t, n_f)\mathbf{\Lambda}(n_f, n_f)\mathbf{V}^T(n_r, n_r) \tag{24}$$

From a mathematical point of view, the value of n_f is equal to the rank of array **A**, that is, it is equal to the smallest number of linearly independent vectors that allow to reconstruct this matrix. The value of n_f is always less than or equal to n_s, and therefore much less than n_r (i.e. $n_f \leq n_s << n_r$). For the reaction under study, the value of $n_f = n_s$ coincides with the number of UV-vis absorbing species [10].

We now apply the SVD technique to reduce the size of array **A**, and thus the size of matrix **R**. It suffices to multiply by the right Eq. 22 by **V**,

$$\mathbf{R}_u = \mathbf{RV} = \mathbf{AV} - \mathbf{C}(\mathbf{SV}) = \mathbf{A}_u - \mathbf{CS}_u \tag{25}$$

Note that the dimensions of **R** were (n_t, n_r), while those of $\mathbf{R}_u$ are (n_t, n_f). The size reduction achieved has been substantial. The second step consists of eliminating the linear parameters [11]. This is achieved by replacing the array $\mathbf{S}_u$ in Eq. 25 by its least squares estimate, $\mathbf{S}_u = \mathbf{C}^+\mathbf{A}_u$,

$$\mathbf{R}_u(\kappa, K) = (\mathbf{I} - \mathbf{CC}^+)\mathbf{A}_u \tag{26}$$

where $\mathbf{C}^+ = (\mathbf{C}^T\mathbf{C})^{-1}\mathbf{C}^T$ stands for the Penrose pseudo-inverse array (operator $(^+)$), and **I** is the identity matrix. This algebraic manipulation makes the residuals to depend only on the parameters related to the rate law. Finally, the kinetic parameters are calculated by minimizing the objective least-squares function, where (tr) is the trace matrix operator,

$$\phi(\kappa, K) = \mathrm{tr}(\mathbf{R}_u\mathbf{R}_u^T) \tag{27}$$

8.2.1. Software

The calculations were carried out using the OPKMCR algorithm programmed in the Julia language (unpublished) [12]. The program was written by Professor F. Pérez-Pla, member of the ICMUV staff.

9. Recycling experiments

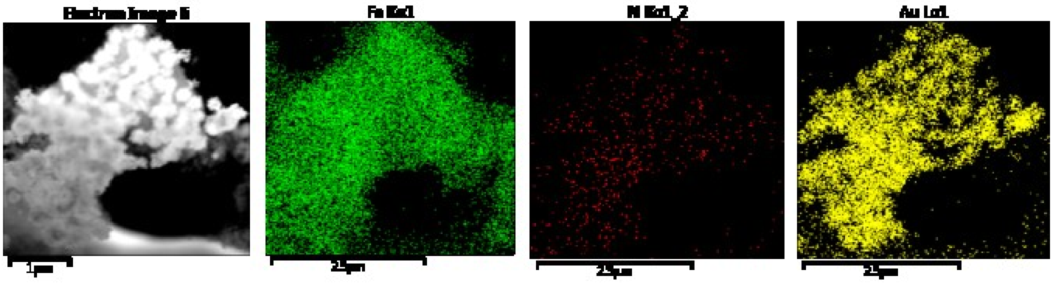

Figure S11. STEM-HAADF and EDX mapping of recycled C_6 catalyst.

10. $TOF_{1/2}$ values for selected catalysts calculated from bibliographic data

Table S1. $TOF_{1/2}$ values calculated from bibliographic data for the reduction of 4-nitrophenol with $NaBH_4$ in water at r.t. catalyzed by Cu based catalysts.

n	ref.	catalyst	[NP] /M $\times10^4$	$[NaBH_4]$ /M $\times10^2$	[C] /M $\times10^5$	k /min^{-1}	$TOF_{1/2}$ /h^{-1} $\times10^{-2}$
1	[13]	Cu/Pd @ ZIF-67	1.11	0.000	3.70	277	359
2	[14]	Cu/Ni@FeO	1.20	4.800	0.23	2.23	49.4

3	[15]	Cu NPs tannic acid	1.68	1.680	0.02	0.11	46.7
4	[16]	CuO NPs	2.40	0.002	0.02	0.09	38.3
5	[17]	Cu diimine complex	0.95	21.000	0.03	0.25	29.7
6	[18]	Au/Cu aerogel	2.26	10.900	0.94	2.21	23.0
7	[19]	$CuFe_2O_4$ MNPs	1.61	3.230	1.35	3.48	18.0
8	[20]	$Co_{40}Cu_{60}$ @ S16LC-20	0.51	0.387	0.45	2.28	11.2
9	[21]	Cu @ SiO_2 @ C-Ni/700	1.00	2.640	0.24	0.56	10.2
10	†	C7 Cu @ PDA @ Fe_3O_4					7.0
11	[22]	Cu/Cu_2O @ CN	0.75	2.980	3.49	7.56	6.9
12	[23]	Cu@Fe_2O_3	667	20.000	333.00	0.62	5.3
13	[24]	Cu/Cu_2O @ Mg AlO-rGO	0.74	0.919	2.21	3.32	4.8
14	[25]	Cu NPs @ CS @ Fe_3O_4	0.90	9.000	0.42	0.35	3.2
15	[26]	Cu @ Cu_2O-CuNiAl-(O)/rGO	0.74	0.923	5.39	4.58	2.7
16	[27]	CuO/Cu_2O	1.09	3.620	2.27	0.60	1.2
17	[28]	Cu@PS NC	12.50	12.500	27.20	0.62	1.2
18	†	C9 Ag/Cu @ PDA @Fe_3O_4					1.2
19	[6]	Cu@CN	1.50	1.250	5.90	0.85	0.94
20	[27]	Ag NPs CuO/Cu_2O	1.09	3.620	4.90	0.90	0.87
21	[29]	Cu_2O@CMK-8	0.74	0.909	6.21	1.33	0.68
22	[22]	Cu/Cu_xO@C	0.75	2.980	3.55	0.66	0.60
23	[30]	CuO NPs	0.50	0.500	10.10	2.70	0.58
24	[31]	Cu@ZIF-Co/Zn	0.88	0.877	5.35	0.80	0.56
25	[31]	Cu@ZIF-67	0.88	0.877	5.99	0.76	0.48
26	[32]	Ni@Cu@Pd	0.71	2.120	7.55	1.10	0.44
27	[33]	Cu@ZC	0.67	1.670	17.30	2.39	0.40
28	[26]	Cu/Ni/Al(O)	0.74	0.923	6.05	0.60	0.32
29	[34]	Cu NP cages	1.00	4.000	9.92	0.62	0.27
30	[35]	CuO NPs	12.5	12.500	74.50	0.35	0.26
31	[32]	Ni@Cu Nps	0.71	2.120	7.72	0.61	0.24
32	[36]	Cu@MCM-41	0.13	0.830	1.57	0.54	0.20
33	[37]	Cu/Cs@CMC	1.08	3.300	827.00	22.98	0.13
34	[6]	Cu@CS	1.50	1.250	17.70	0.35	0.13
35	[37]	Cu@CMC	1.08	3.300	481.00	12.60	0.12
36	[28]	Cu NPs	12.5	12.500	315.00	0.58	0.099
37	[38]	Pd @ AuCu6L core–shell NCs	0.33	8.740	7.60	0.50	0.095
38	[39]	CuOC	0.66	9.920	4.16	0.12	0.080
39	[40]	Au/Cu@rGO	2.00	1.670	647.00	5.76	0.077
40	[41]	Hollow porous Cu particles	1.00	4.000	31.50	0.56	0.077
41	[42]	Cu nanowires	3.33	1.670	47.70	0.25	0.076
42	[43]	$SrCu_{0.3}Fe_{11.7}O_{19}$	2.00	20.000	62.70	0.55	0.076
43	[38]	Pd@AuCu12L core–shell NCs	0.33	8.740	7.42	0.37	0.071
44	[44]	Cu_2O	0.75	0.250	34.90	0.74	0.069
45	[45]	Cu porous microspheres	1.48	0.617	19.00	0.18	0.061
46	[46]	CuO	30.80	1.920	3870.00	1.14	0.039
47	[47]	micro/nanostructured porous Cu microspheres	0.81	3.240	30.50	0.32	0.036
48	[45]	Cu	1.48	0.617	19.40	0.09	0.031
49	[48]	Cu NCs	0.68	1.120	60.00	0.61	0.030
50	[49]	Cu tartatre complex	4.00	0.600	315.00	0.37	0.021
51	[50]	Cu_2O	0.91	0.455	212.00	0.94	0.017
52	[51]	Cu/Ag@taro-rhizo powder	8.57	7.140	1130.00	0.31	0.010

53	[52]	N-C/Cu/N-C-700	6.25	1.130	977.00	0.25	0.007
54	[53]	Cu@PMCs	1.67	1.000	262.00	0.25	0.007
55	[54]	Ni/Cu nanowires	0.75	1.250	682.00	0.91	0.004
56	[53]	Cu@CNBs	1.67	1.000	262.00	0.13	0.003
57	[44]	Cu_2O DHP	0.75	0.250	349.00	0.33	0.003
58	[55]	Cu@MA	0.83	16.700	496.00	0.42	0.003
59	[56]	Cu@AG-sponge	1.11	2.860	540.00	0.32	0.003
60	[53]	Cu@SCMs	1.67	1.000	262.00	0.08	0.002
61	[57]	Cu@alginate/banana waste beads	1.08	1.670	540.00	0.21	0.002
62	[58]	Cu@CMC-PSIS	0.83	0.017	399.00	0.18	0.002
63	[59]	Cu/Ag@vigna radiata	0.83	0.017	826.00	0.28	0.001
64	[49]	Cu-commercial	4.00	0.600	315.00	0.02	0.0009
65	[60]	Cu NPs	0.60	1.000	393.00	0.10	0.0006
66	[61]	Cu@CH-LDH	1.71	0.014	5060.48	0.36	0.0005
67	[62]	Cu dopped glasses	0.50	1.250	41.90	0.01	0.0003
68	[63]	Ag/CuO@G1	0.74	0.988	5740.00	0.55	0.0003
69	[61]	Cu @ CH	1.71	0.014	1975.81	0.07	0.0003

Table S2. $TOF_{1/2}$ values calculated from bibliographic data for the reduction of 4-nitrophenol with $NaBH_4$ in water at r.t. catalyzed by Ag based catalysts.

n	ref.	catalyst	[NP] /M $\times 10^4$	[$NaBH_4$] /M $\times 10^2$	[C] /M $\times 10^5$	k /min^{-1}	$TOF_{1/2}$ /h^{-1}
1	[64]	Ag/NiO	0.05	1.27	0.000001	0.96	2847872
2	[65]	Ag NPs	10.5	2.09	0.0001	0.17	1040995
3	[66]	Ag@PDA/SBA-15 (0.6)	20.0	99.0	0.001	0.89	634744
4	[67]	Ag @ PPAA	0.66	3.28	0.000	0.93	494937
5	[68]	Ag@MR-3	12.5	6.25	0.001	0.14	82286
6	[69]	Ag@PDA@ZrP	1.13	3.56	0.01	2.09	8763
7	[70]	Ag NPs@MPTMS @ TiO_2 (7)	10.0	5.00	1.21	23.66	8444
8	[71]	Ag @ PCL-Ala-PTHF	64.2	13.2	0.34	0.69	5642
9	[72]	Ag @ pBNNS (air)	1.20	6.24	0.09	9.01	5153
10	[73]	Ag@Zn-BIF	21.6	20.0	0.24	1.29	5097
11	[74]	Au/Ag 1:9	1.36	2.98	0.07	5.86	5069
12	[75]	Ag@TiO_2 2	0.60	3.00	0.01	1.38	4069
13	[76]	Ag@MX/PAM	5.00	7.55	0.53	5.84	2384
14	[77]	Ag @ ZA4-MW	0.07	0.09	0.01	7.44	2311
15	[78]	Ag@SBA-16	0.79	0.80	0.01	0.38	1901
16	[79]	Ag 2.6@ C 15h	0.63	3.13	0.13	7.52	1556
17	[80]	Ag-Pt (9:1) NPs	1.02	0.06	0.12	3.54	1316
18	[81]	MSAg-50	1.33	1.33	0.05	1.18	1259
19	[82]	Ag@ZIF-7	1.59	1.27	0.03	0.62	1239
20	[83]	Ag@Fe_3O_4	3.57	10.6	0.02	0.11	887
21	†	Ag@PDA@Fe_3O_4 (C1)	0.92	6.67	–		765
22	[78]	Ag/Ni@SBA-16	0.79	0.80	0.08	1.73	716
23	†	Ag@PDA@Fe_3O_4 (C8)	0.92	6.67	–		700
24	†	Ag@PDA@Fe_3O_4 (C2)	0.92	6.67	–		680
25	[84]	Ag @ ZnO/MWCNT	0.77	0.97	0.03	0.66	660
26	[85]	Ag NPs@rGO	0.85	4.27	0.15	2.60	622
27	[84]	Ag @ ZnO	0.77	0.97	0.04	0.68	567
28	[86]	Ag@HMCSs	1.50	12.5	0.15	1.15	511

29	[87]	Cu/Ag @ BNPs	1.10	2.75	0.06	0.54	458
30	[88]	Ag NPs@SiNSs	1.13	1.88	0.57	4.81	413
31	[87]	Au/Ag@BNPs	1.10	2.75	0.06	0.48	406
32	[89]	Ag @ BNNs	0.63	6.67	0.01	0.10	374
33	[90]	Ag/CeO_2 @ SBA-15	1.50	3.00	0.26	0.96	238
34	[72]	Ag @ pBNNS (N_2)	1.20	6.24	0.09	0.39	226
35	[91]	Pd/Ag @ FA	0.10	0.10	0.01	0.72	222
36	[92]	Ag NPs@NC	9.09	1.82	0.35	0.19	217
37	[93]	Ag@TA-CFR	1.05	6.48	0.37	1.75	216
38	[94]	L-AgNPs	3.00	3.00	0.28	0.46	215
39	[95]	Ag@sulfamine-resin (photo)	12.5	125	0.41	0.15	200
40	[72]	Ag @ SiO_2 (air)	1.20	6.24	0.10	0.34	180
41	[71]	Ag @ PCL-Aspar-PTHF	64.2	13.2	0.43	0.03	178
42	[96]	Ag@m-Hap-Si(S)	6666.67	100	2667	1.50	162
43	[72]	Ag @ SiO_2 (N_2)	1.20	6.24	0.10	0.29	154
44	[97]	Ag @γ-Al_2O_3 cal	3.07	5.25	0.20	0.22	147
45	[90]	Ag @ SBA-15	1.50	3.00	0.27	0.60	146
46	[98]	rGO/CNT/Fe/Ag hybrid	0.89	9.26	0.27	0.88	127
47	[93]	Ag @ CFR	1.05	6.48	0.14	0.37	123
48	[99]	AgNPs @ SNTs-4	0.60	1.00	0.50	2.30	120
49	†	Ag@PDA@Fe_3O_4 (C3)	0.92	6.67	–		115
50	[100]	Ag@DODA-PMo_{12}	0.99	0.99	0.15	0.39	112
51	[88]	Ag NPs @ Si NSs	1.13	1.88	0.47	1.08	111
52	[101]	Ag NPs	1.27	6.70	0.03	0.05	110
53	[102]	Ag @ $Ca_2Nb_3O_{10}$	0.91	1.82	0.44	1.09	96.7
54	[103]	CA-Ag NPs beads	0.94	0.94	0.11	0.22	84.0
55	[104]	Ag/CeO_2@KIT-6	4.00	10.0	1.04	0.48	79.6
56	[105]	Ag/Ni @ ZnO	1.67	2.00	0.79	0.85	78.2
57	[106]	Ag @ C-TiO_2 @ Fe_3O_4	6.67	1.00	17.4	3.78	62.6
58	[84]	Ag @ MWCNT	0.77	0.97	0.15	0.27	61.4
59	[107]	Ag @ SiO_2	0.97	1.94	0.23	0.30	55.6
60	[108]	Ag@Pd satellites-Fe_3O_4	0.95	1.32	1.57	1.98	51.9
61	[95]	Ag @ sulfamine-resin (os)	12.5	125	0.41	0.04	50.4
62	[97]	Ag @γ-Al_2O_3 no cal	3.07	5.25	0.55	0.19	46.2
63	[109]	Ag @ NFC @ Fe_3O_4	2.50	6.25	6.12	2.44	43.2
64	[110]	Ag @ C Fiber	14.4	17.2	1.20	0.08	41.5
65	[111]	Ag @SLPF	0.91	0.91	0.97	0.86	34.9
66	[111]	Ag @ PF	0.91	0.91	0.23	0.19	32.4
67	[112]	Ag@SBA-15@Fe_3O_4	1.33	3.33	1.85	0.96	29.9
68	[113]	Ag NPs @ F-rGO	2.40	1.82	4.25	1.20	29.4
69	[114]	Ag/CeO_2	1.50	1.53	1.51	0.66	28.1
70	[115]	Ag@NC	0.80	10.0	0.31	0.25	27.8
71	[116]	Ag @ GLH-20	20.0	12.0	29.0	0.91	27.1
72	[117]	Ag @ PCTP @ Fe_3O_4 (MPCTP)	0.33	0.08	0.42	0.60	20.9
73	[114]	Ag/CeO_2 red	1.50	1.53	1.51	0.47	20.0
74	[118]	Ag@PF@ Fe_3O_4	7.50	7.50	9.27	0.52	18.1
75	[104]	Ag/CeO_2@TUD-1	4.00	10.0	1.17	0.12	17.8
76	[119]	Ag@AgCl Dual 4	6.33	3.33	3.05	0.18	16.4
77	[120]	Ag@MWCNTs-polymer	0.05	0.25	0.08	0.47	13.2
78	[121]	Ag @ TiO_2 (citric ac.)	0.50	5.00	9.36	5.65	13.1
79	[114]	Ag@CeO_2	1.50	1.53	1.51	0.23	10.0
80	[122]	Ag/AgCl @ PO	1.00	1.00	2.00	0.36	7.8
81	[123]	Ag @ CTAB/NCC	2.50	6.25	12.1	0.85	7.7

82	[124]	AgNPs@Pro-ESM	2.57	3.43	2.86	0.18	6.8
83	[125]	Ag@APC	0.91	4.55	2.22	0.33	5.9
84	[126]	Ag@TPMMs-MAA	2.00	5.00	25.5	1.72	5.8
85	[121]	Ag @ TiO_2 (UV light)	0.50	5.00	1.67	0.29	3.7
86	[127]	Ag @ LDH	0.72	0.52	2.94	0.35	3.7
87	[128]	Cu-CuO-Ag (CA3)	7.69	0.92	55.0	0.58	3.5
88	[129]	Ag NPs @ PC	8.33	1.67	55.8	0.47	3.0
89	[130]	Ag NPs	0.83	1.67	2.50	0.17	2.5
90	[122]	Ag/AgCl @ POGO	1.00	1.00	4.00	0.22	2.3
91	[131]	Co/Ag @ spongin	8.33	0.17	141	0.86	2.2
92	[132]	Ag @ rGO-PD-MCNT	0.98	0.65	13.1	0.66	2.1
93	[133]	AgNPs @ CV	0.83	1.67	19.1	1.09	2.1
94	[134]	Ag @ MGO-PDA	0.60	4.00	6.45	0.50	2.0
95	[135]	MCS-3	0.72	25.0	41.3	2.27	1.7
96	[136]	Ag NPs @ AAL	0.99	2.97	5.88	0.23	1.7
97	[137]	AgNPs @ LSR	0.60	0.01	1.06	0.07	1.6
98	[138]	Ag @ ZrGP S2	0.83	8.33	4.67	0.18	1.4
99	[137]	AgNPs @ LSA	0.60	0.01	1.30	0.06	1.3
100	[138]	Ag @ ZrGP S1	0.83	8.33	2.81	0.10	1.2
101	[139]	Ag @ C@ Fe_3O_4	12.5	2.50	21.1	0.04	1.1
102	[51]	Ag/Cu @ TP	8.57	7.14	113	0.31	1.0
103	[140]	Ag NPs-LR	8.33	1.67	70.3	0.19	1.0
104	[131]	Ag @ spongin	8.33	0.17	129	0.33	0.9
105	[141]	Ag @ ZnO-CH	8.57	7.14	98.8	0.17	0.6
106	[121]	Ag @ TiO_2 ($NaBH_4$)	0.50	5.00	9.92	0.27	0.6
107	[142]	Ag@PIN@Fe_3O_4	4.79	4.41	34.6	0.09	0.6
108	[143]	Ag @PPE/CNF	0.50	500	6.27	0.14	0.5
109	[144]	Ag NPs	0.83	1.67	23.3	0.26	0.4
110	[133]	Ag Nps @ QBC	0.83	1.67	24.4	0.25	0.4
111	[145]	Ag solid	0.91	1.82	23.7	0.20	0.3
112	[146]	Ag NPs @ NF	0.83	1.67	24.1	0.16	0.2
113	[147]	Ag NPs @ WBS	0.83	1.67	68.7	0.31	0.2
114	[108]	Ag @ SiO_2 @ Fe_3O_4	0.61	0.59	140	0.46	0.1
115	[148]	Ag NPs @ rGO-LS	0.33	3.33	595	1.89	0.05
116	[55]	Ag @ MA	0.83	16.7	413	0.27	0.02
117	[149]	Ag @ SiO_2 @ Fe_3O_4	0.03	0.01	23.7	0.39	0.02
118	[150]	Ag NPs	0.02	0.03	7.73	0.17	0.02
119	[151]	AgNPs @ CP	0.83	1.67	60.8	0.00	0.002

Table S3. $TOF_{1/2}$ values calculated from bibliographic data for the reduction of 4-nitrophenol with $NaBH_4$ in water at r.t. catalyzed by Au based catalysts.

n	ref.	catalyst	[NP] /M $\times 10^4$	[$NaBH_4$] /M $\times 10^2$	[C] /M $\times 10^5$	k /min^{-1}	$TOF_{1/2}$ /h^{-1}
1	[152]	Au–GO nanocomposite	6.67	19.7	0.33	6.48	56451
2	[153]	Au-Ce-MOF	1.00	1.47	0.01	0.32	18414
3	[154]	Au/Fe_2O_3-Au/Fe_2O_3	0.74	0.93	0.01	0.50	16165
4	[155]	Au NRs/PAN-g-PEI	400	200	3.50	0.31	15334
5	[40]	Au3 -Cu1 /rGO	2.00	1.67	0.65	5.76	7701
6	[156]	Au@DHBC NPs	6.48	19.2	0.32	0.77	6643
7	[157]	ACW	8.33	10.00	0.85	1.45	6181
8	[74]	Au/Ag (1:9)	1.36	2.9	0.68	5.86	5069

9	[158]	Au/g-C_3N_4-NS	0.66	3.30	0.07	0.97	4263
10	[159]	Au@TiO_2 hollow NFs	0.33	10.0	0.02	0.64	4216
11	[160]	NAP-Mg-Au(0)	431	200	20.4	0.46	4168
12	[161]	Fe_3O_4@C@Cys-Au NRs	1.33	0.67	0.44	3.18	4129
13	[162]	Au-SnO_2/SiO_2	1.67	0.50	1.61	6.64	2979
14	[18]	AuCu aerogel	2.26	10.9	0.74	2.21	2914
15	[163]	PCNFs-Au	5.00	6.00	5.45	6.27	2491
16	[164]	DFNS-SH/Au	0.69	9.22	0.10	0.78	2396
17	[165]	Au/AC	1.33	3.33	2.49	8.08	1875
18	[166]	Au@CCS	1.00	0.50	0.03	0.11	1731
19	[167]	Au@β-CDP-N	3.13	22.0	6.92	8.70	1704
20	[153]	AuNPs/CeO_2	1.00	1.47	0.06	0.21	1591
21	[168]	Au@NH_2-MIL-101(Fe) (60)	1.00	5.00	0.63	2.16	1490
22	†	Au@PDA@Fe_3O_4 (C4)	0.92	10.0	0.00	–	1434
23	[169]	AuAg2 NPs	0.45	2.27	0.82	5.94	1425
24	[170]	Au@CuO_x-CeO_2 CSNs	0.99	16.56	0.47	1.56	1423
25	[89]	Au-BNNs	0.63	6.25	0.03	0.15	1180
26	[171]	AuNWs	25.00	25.0	3.72	0.39	1133
27	[172]	Fe_3O_4@COF-Au	10.00	44.0	0.86	0.22	1113
28	[173]	Au-Pd NPs (GK)	0.20	2.00	0.027	0.29	930
29	[174]	SiO_2 @Au@SiO_2 NTs	0.97	17.0	0.25	0.52	857
30	†	Au@PDA@Fe_3O_4 (C5)	0.00	10.0	0.00	–	824
31	[175]	P-S1-Au	0.60	0.60	0.06	0.19	821
32	[176]	Au/FMOF	4.88	4.24	4.47	1.72	813
33	[177]	2-D Au NSs	1.94	0.32	1.60	1.18	616
34	[178]	Au@Fe_3O_4@polymer	6.82	20.2	1.73	0.35	604
35	[179]	Au NPs@chitosan composite	4.76	0.95	15.11	3.37	459
36	[180]	Au@CNFs	0.60	5.00	0.19	0.33	442
37	[179]	Au NPs/Fe_3O_4/chitosan	4.76	0.95	15.11	2.83	385
38	[181]	Au-NPs@ P6 @ g-C_3N_4	2.00	26.4	0.66	0.29	381
39	[182]	Au/RA-MC-2	2.39	9.80	0.80	0.28	364
40	[173]	Au-Ag NPs (GK)	0.20	2.00	0.077	0.32	359
41	[183]	Fe_3O_4-200 nm@TA@Au	1.25	2.50	4.82	3.09	347
42	[184]	Au/PMMA	0.51	8.16	0.30	0.47	346
43	[185]	Au@g-C_3N_4	0.72	0.39	0.44	0.48	338
44	†	Au@PDA@Fe_3O_4 (C6)	0.92	6.20	0.00	–	313
45	[182]	Au/OMC-2	2.39	9.80	0.80	0.20	260
46	[186]	Pt3Au1-PDA/RGO	1.00	0.00	1.00	0.57	249
47	[187]	Au/p(Aam-co-MTM) hydrogel	100	10.0	25.4	0.14	235
48	[167]	Au@β-CDP-C	3.13	22.0	34.6	5.82	228
49	[188]	Au-Ag@PCP	1.17	0.52	0.39	0.17	222
50	[189]	Au NPs@SCOFS@BP	0.76	20.0	1.68	1.10	216
51	[190]	AuNS@pSiO_2	0.99	15.9	1.87	0.88	203
52	[191]	Au-5@RCC3	1.00	1.65	1.07	0.39	157
53	[186]	Pt1Au1-PDA/RGO	1.00	0.00	1.00	0.34	147
54	[192]	Au-1/Co@N-C	2.42	29.0	15.79	2.18	145
55	[182]	Au/NCN-2	2.39	9.80	0.80	0.10	130
56	[162]	Au/SiO_2	1.67	0.50	0.40	0.07	120
57	[193]	Au-m-Co_3O_4	1.35	1.08	4.12	0.83	118
58	[170]	Au@CeO_2 CSNs	0.99	16.6	1.08	0.27	108
59	[188]	PCP@Au	1.17	0.52	0.33	0.06	99.9
60	[194]	AuNPs/SNTs nanocomposite	0.60	0.15	1.67	0.64	99.5

61	[195]	Au NPs	0.70	1.16	0.50	0.16	94.4
62	[186]	Pt-PDA/rGO	1.00	0.00	1.00	0.21	89.1
63	[196]	Au-S-COF	1.25	20.8	2.54	0.37	78.2
64	[197]	Au@g-C_3N_4	1.82	0.73	9.23	0.90	76.7
65	[198]	α-CD-capped Au 11 nm	1.10	0.50	2.00	0.28	66.4
66	[199]	Au-PAF-162	1.04	16.0	8.33	1.14	61.9
67	[200]	Au- PAFs-96	1.04	16.0	9.03	1.14	57.1
68	[200]	Au-PAF-95	1.04	16.0	6.39	0.78	55.2
69	[201]	Au@PD-COP-II	1.80	7.93	2.52	0.17	52.8
70	[186]	Au-PDA/rGO	1.00	0.00	1.00	0.12	51.9
71	[198]	α-CD-capped Au 20 nm	1.10	0.50	2.00	0.21	49.3
72	[201]	Au@PD-COP-I	1.80	7.93	2.08	0.13	47.5
73	[198]	α-CD-capped Au 26 nm	1.10	0.50	2.00	0.18	42.6
74	[202]	Fe_3C/Au@NG	0.95	0.48	29.7	1.86	25.8
75	[203]	Au@PZS@CNTs nanohybrid	1.13	0.50	2.13	0.11	24.6
76	[204]	Au NPs/GO/Fe_3O_4/PDA	13.8	55.4	39.1	0.14	22.1
77	[205]	Gold nanocomposite thin film.	0.04	9.78	0.01	0.01	21.2
78	[178]	Au@Fe_3O_4 modif./polymer	6.82	20.2	1.73	0.01	20.8
79	[206]	micelle-suppor ted Au nanoparticles	1.07	0.36	2.14	0.09	19.2
80	[207]	Au/graphene hydrogel	0.93	0.67	4.06	0.19	18.9
81	[186]	Pt1Au1-RGO	1.00	0.00	1.00	0.04	17.9
82	[208]	Au@C-PCTF	0.95	2.38	22.3	0.95	17.6
83	[178]	Au@Fe_3O_4modif./SiO_2	6.82	20.2	1.73	0.01	15.4
84	[209]	A/PDA@PDMAEMA	1.59	6.35	5.80	0.13	15.4
85	[210]	Au/PANI	14.6	21.9	388	0.71	11.5
86	[211]	Au-91Pir-HNTs-NH_2	0.13	6.67	2.69	0.51	11.0
87	[212]	Au-Fe_3O_4@carbon yolk-shell	1.07	7.32	9.91	0.12	5.6
88	[205]	Gold nanocomposite thin film	0.04	9.78	0.02	0.01	4.4
89	[213]	Au@Cu_2O (24:1)	2.00	1.50	53.7	0.20	3.2
90	[208]	Au@PCTF	0.95	2.38	28.4	0.21	3.0
91	[134]	Au@MGO-PDA	0.60	4.00	37.2	0.43	3.0
92	[121]	TiO_2@Au-C $NaBH_4$	0.50	5.00	72.6	0.83	2.5
93	[121]	TiO_2@Au-B $NaBH_4$	0.50	5.00	101	1.15	2.5
94	[214]	Au@MSNSs	14.3	5.71	89.4	0.04	2.4
95	[215]	3Au/g-C_3N_4	0.12	1.76	5.38	0.24	2.3
96	[216]	hematite/Au MS	1.00	2.00	135	0.68	2.2
97	[121]	TiO_2@Au-D $NaBH_4$	0.50	5.00	40.1	0.37	2.0
98	[217]	Au dopped mesoporous boehmite film	1.00	1.00	28.0	0.10	1.6
99	[218]	Au NPs	1.00	1.00	27.2	0.09	1.4
100	[211]	HNTs-NH_2-Au.	0.13	6.67	2.33	0.01	0.3
101	[219]	Au@HEP	0.91	0.91	104	0.07	0.3
102	[219]	Au@PEI	0.91	0.91	76.3	0.05	0.2
103	[219]	Au@APP	0.91	0.91	98.8	0.03	0.1
104	[121]	TiO_2@Au-A citric acid	0.50	5.00	68.5	0.03	0.1
105	[220]	Au/CMC-CH NPs	0.83		168	0.02	0.041
106	[220]	Au/CMC-CHZY NPs	0.83		687	0.04	0.019
107	[221]	RG-SMS	1.38	9.86	3914	0.07	0.011
108	[121]	TiO_2@Au-E sunlight	0.50	5.00	36.6	0.002	0.011
109	[222]	Au-CeO_2 @ZrO_2	0.73	0.09	6927	0.02	0.001
110	[222]	Au@ZrO_2	0.73	0.09	6927	0.01	0.0004

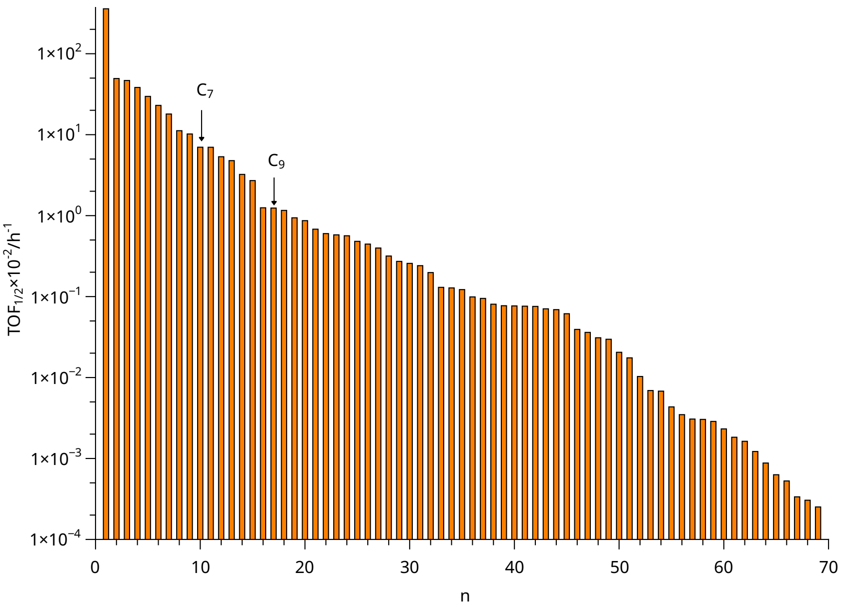

Figure S12. $TOF_{1/2}$ values (logarithmic scale) calculated from bibliographic data gathered in Table S1. The arrows show the order number (*n*) of catalysts C_7 (10/69, Q_1) and C_9 (18/69, Q_2).

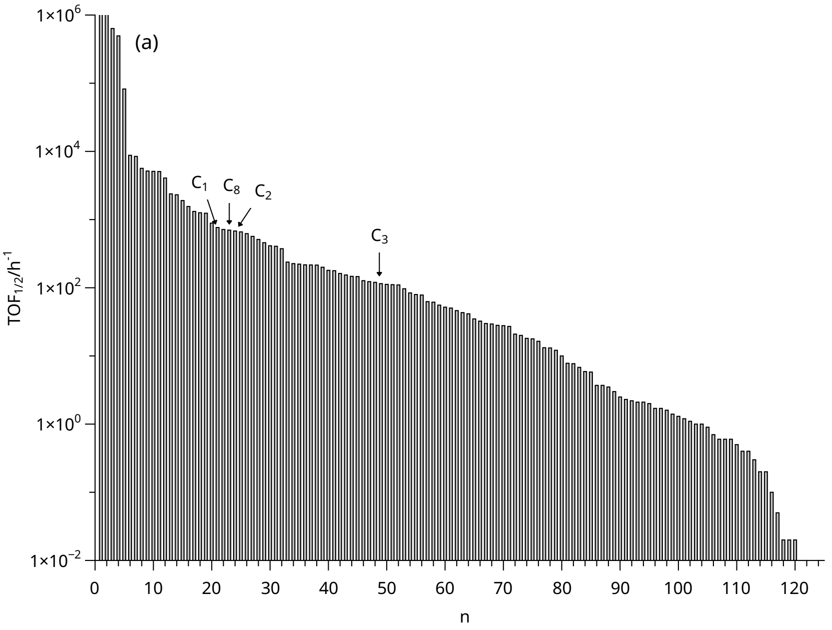

Figure S13. $TOF_{1/2}$ values (logarithmic scale) calculated from bibliographic data gathered in Table S2. The arrows show the order number (*n*) of catalysts C_1 (21/121, Q_1), C_8 (23/121, Q_1), C_2 (24/121, Q_1) and C_3 (49/121, Q_2 (Q = quartile)).

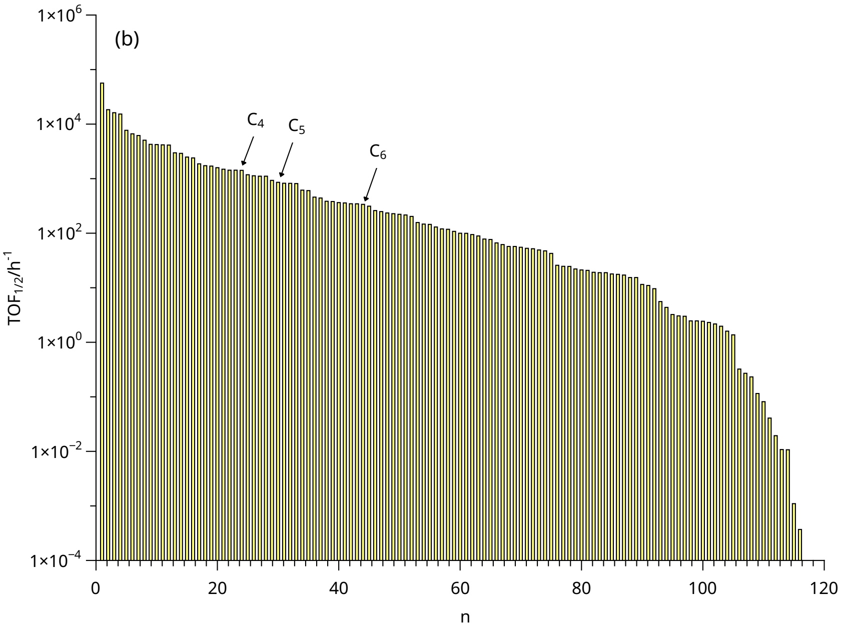

Figure S14. $TOF_{1/2}$ values (logarithmic scale) calculated from bibliographic data gathered in Table S3. The arrows show the order number (n) of catalysts C_4 (22/110, Q_1), C_5 (30/110, Q_2) and C_6 (44/110, Q_2). (Q = quartile)

References

1. Gelder, E.A.; Jackson, S.D.; Lok, C.M. The hydrogenation of nitrobenzene to aniline: a new mechanism. *Chem. Commun.* **2005**, pp. 522–524. https://doi.org/10.1039/B411603H.
2. Corma, A.; Concepción, P.; Serna, P. A different reaction pathway for the reduction of aromatic nitro compounds on gold catalysts. *Angew. Chem. Int. Ed.* **2007**, *46*, 7133–7133. https://doi.org/10.1002/anie.200790188.
3. Kadam, H.K.; Tilve, S.G. Advancement in methodologies for reduction of nitroarenes. *RSC Adv.* **2015**, *5*, 83391–83407. https://doi.org/10.1039/c5ra10076c.
4. Kojima, Y.; ichirou Suzuki, K.; Fukumoto, K.; Sasaki, M.; Yamamoto, T.; Kawai, Y.; Hayashi, H. Hydrogen generation using sodium borohydride solution and metal catalyst coated on metal oxide. *Int. J. Hydrogen Energy* **2002**, *27*, 1029–1034. https://doi.org/10.1016/s0360-3199(02)00014-9.
5. Demirci, U.B.; Miele, P. Cobalt in NaBH4 hydrolysis. *Phys. Chem. Chem. Phys.* **2010**, *12*, 14651. https://doi.org/10.1039/c0cp00295j.
6. Mounir, C.; Ahlafi, H.; Aazza, M.; Moussout, H.; Mounir, S. Kinetics and Langmuir–Hinshelwood mechanism for the catalytic reduction of para-nitrophenol over Cu catalysts supported on chitin and chitosan biopolymers. *React. Kinet., Mech. Catal.* **2021**, *134*, 285–302. https://doi.org/10.1007/s11144-021-02066-w.
7. Baxter, R.J.; Hu, P. Insight into why the Langmuir–Hinshelwood mechanism is generally preferred. *J. Chem. Phys.* **2002**, *116*, 4379–4381. https://doi.org/10.1063/1.1458938.
8. Malinowski, E.R. *Factor Analysis in Chemistry*, 2 ed.; Wiley Interscience: N. York, 1991.
9. Press, W.H.; Teukolsky, S.A.; Vetterling, W.T.; Flannery, B.P. Numerical Recipes in C. The Art of Scientific Computing.; Cambrigde University Press, 1992; chapter 11, pp. 456–495.
10. Meloun, M.; Syrový, T.; Vrána, A. Determination of the number of light-absorbing species in the protonation equilibra of selected drugs. *Anal. Chim. Acta* **2003**, *489*, 137–151.
11. Bonneau, R.; Wirz, J.; Zuberbuhler, A.D. Methods for the analysis of transient absorbance data (Technical Report). *Pure Appl. Chem.* **1997**, *69*, 979–992. https://doi.org/10.1351/pac199769050979.
12. Bezanson, J.; Karpinski, S.; Shah, V.B.; Edelman, A. Julia: A fast dynamic language for technical computing. *CoRR* **2012**, *abs/1209.5*.
13. Gholinejad, M.; Naghshbandi, Z.; Sansano, J.M. Zeolitic imidazolate frameworks-67 (ZIF-67) supported PdCu nanoparticles for enhanced catalytic activity in Sonogashira-Hagihara and nitro group reduction under mild conditions. *Molecular Catalysis* **2022**, *518*, 112093. https://doi.org/10.1016/j.mcat.2021.112093.
14. Zhang, M.; Cao, A.; Zhang, H.; Yang, C. Defective MNiFeO (M = Cu, Zn, Co, Mn) NRs derived from cation-exchanged Fe_2Ni-MOFs for catalytic nitroarene hydrogenation. *J. Colloid Interface Sci.* **2022**, *623*, 63–76. https://doi.org/10.1016/j.jcis.2022.04.177.
15. Liu, F.; Liu, X.; Chen, F.; Fu, Q. Tannic Acid: A green and efficient stabilizer of Au, Ag, Cu and Pd nanoparticles for the 4-Nitrophenol Reduction, Suzuki–Miyaura coupling reactions and click reactions in aqueous solution. *J. Colloid Interface Sci.* **2021**, *604*, 281–291. https://doi.org/10.1016/j.jcis.2021.07.015.
16. Tamuly, C.; Saikia, I.; Hazarika, M.; Das, M.R. Reduction of aromatic nitro compounds catalyzed by biogenic CuO nanoparticles. *RSC Adv.* **2014**, *4*, 53229–53236. https://doi.org/10.1039/c4ra10397a.
17. Ahmad, A.; Shah, S.N.A.; Arshad, M.; Bélanger-Gariepy, F.; Tiekink, E.R.; Rehman, Z. A copper diimine-based honeycomb-like porous network as an efficient reduction catalyst. *Appl. Organomet. Chem.* **2020**, *35*. https://doi.org/10.1002/aoc.6065.
18. Qin, J.; Tan, X.; Feng, F.; Li, H. Facile and controllable synthesis of AuCu aerogels for the enhanced degradation of 4-nitrophenol. *Appl. Surf. Sci.* **2021**, *561*, 150024. https://doi.org/10.1016/j.apsusc.2021.150024.
19. Feng, J.; Su, L.; Ma, Y.; Ren, C.; Guo, Q.; Chen, X. $CuFe_2O_4$ magnetic nanoparticles: A simple and efficient catalyst for the reduction of nitrophenol. *Chem. Eng. J.* **2013**, *221*, 16–24. https://doi.org/10.1016/j.cej.2013.02.009.
20. Deka, J.R.; Saikia, D.; Lu, N.F.; Chen, K.T.; Kao, H.M.; Yang, Y.C. Space confined synthesis of highly dispersed bimetallic CoCu nanoparticles as effective catalysts for ammonia borane dehydrogenation and 4-nitrophenol reduction. *Appl. Surf. Sci.* **2021**, *538*, 148091. https://doi.org/10.1016/j.apsusc.2020.148091.
21. Ding, L.; Hu, J.; Zhang, Y.; Xu, J.; Zhang, M. Copper-Based Nanocatalysts with SiO_2 and Carbon Dual-Layer Coatings and Metallic Ni/CuNi Decoration toward Highly Efficient Nitroaromatics Reduction. *Inorg. Chem.* **2022**, *61*, 1717–1727. https://doi.org/10.1021/acs.inorgchem.1c03582.
22. Jia, W.; Tian, F.; Zhang, M.; Li, X.; Ye, S.; Ma, Y.; Wang, W.; Zhang, Y.; Meng, C.; Zeng, G.; et al. Nitrogen-doped porous carbon-encapsulated copper composite for efficient reduction of 4-nitrophenol. *J. Colloid Interface Sci.* **2021**, *594*, 254–264. https://doi.org/10.1016/j.jcis.2021.03.020.
23. Borah, B.J.; Bharali, P. Direct Hydrogenation of Nitroaromatics at Room Temperature Catalyzed by Magnetically Recoverable Cu@Fe_2O_3 Nanoparticles. *Appl. Organomet. Chem.* **2020**, *34*. https://doi.org/10.1002/aoc.5753.
24. Dou, L.; Wang, Y.; Li, Y.; Zhang, H. Novel core–shell-like nanocomposites xCu@Cu_2O/MgAlO-rGO through an in situ self-reduction strategy for highly efficient reduction of 4-nitrophenol. *Dalton Trans.* **2017**, *46*, 15836–15847. https://doi.org/10.1039/c7dt03276e.
25. Arora, A.; Oswal, P.; Singh, S.; Nautiyal, D.; Rao, G.K.; Kumar, S.; Singh, A.K.; Kumar, A. Organoselenium ligand-stabilized copper nanoparticles: Development of a magnetically separable catalytic system for efficient, room temperature and aqueous phase reduction of nitroarenes. *Inorg. Chim. Acta* **2021**, *522*, 120267. https://doi.org/10.1016/j.ica.2021.120267.

26. Wei, Z.; Feng, D.; Li, J.; Lin, Y.; Zhang, H. Nanosheet array-like Cu@Cu_2O-CuNiAl(O)/rGO composites for highly efficient reduction of nitrophenol: Electronic and structure promotion effect of nickel. *Chem. Eng. J.* **2022**, *427*, 131659. https://doi.org/10.1016/j.cej.2021.131659.
27. Zhang, N.; Xie, R.; Song, W.; Zhang, F.; Chen, L.; Chai, F. Facile synthesis of Ag NPs decorated CuO/Cu_2O composite nanoparticles for enhancing catalytic activity in the hydrogenation of 4- nitrophenol. *ChemistrySelect* **2021**, *6*, 4041–4046. https://doi.org/10.1002/slct.202100815.
28. Taghizadeh, A.; Rad-Moghadam, K. Green fabrication of Cu/pistachio shell nanocomposite using Pistacia Vera L. hull: An efficient catalyst for expedient reduction of 4-nitrophenol and organic dyes. *J. Cleaner Prod.* **2018**, *198*, 1105–1119. https://doi.org/10.1016/j.jclepro.2018.07.042.
29. Rath, P.C.; Saikia, D.; Mishra, M.; Kao, H.M. Exceptional catalytic performance of ultrafine Cu_2O nanoparticles confined in cubic mesoporous carbon for 4-nitrophenol reduction. *Appl. Surf. Sci.* **2018**, *427*, 1217–1226. https://doi.org/10.1016/j.apsusc.2017.08.097.
30. Nasrollahzadeh, M.; Maham, M.; Sajadi, S.M. Green synthesis of CuO nanoparticles by aqueous extract of Gundelia tournefortii and evaluation of their catalytic activity for the synthesis of N -monosubstituted ureas and reduction of 4-nitrophenol. *J. Colloid Interface Sci.* **2015**, *455*, 245–253. https://doi.org/10.1016/j.jcis.2015.05.045.
31. Budi, C.S.; Deka, J.R.; Hsu, W.C.; Saikia, D.; Chen, K.T.; Kao, H.M.; Yang, Y.C. Bimetallic Co/Zn zeolitic imidazolate framework ZIF-67 supported Cu nanoparticles: An excellent catalyst for reduction of synthetic dyes and nitroarenes. *J. Hazard. Mater.* **2021**, *407*, 124392. https://doi.org/10.1016/j.jhazmat.2020.124392.
32. Gong, Z.; Ma, T.; Liang, F. Syntheses of magnetic blackberry-like Ni@Cu@Pd nanoparticles for efficient catalytic reduction of organic pollutants. *J. Alloys Compd.* **2021**, *873*, 159802. https://doi.org/10.1016/j.jallcom.2021.159802.
33. Benmaati, A.; Boukoussa, B.; Aoul, R.H.; Hachemaoui, M.; Kerbadou, R.M.; Zahmani, H.H.; Hacini, S. Insights into Catalytic Reduction of Organic Pollutants Catalyzed by Nanoparticles Supported on Zeolite Clinoptilolite. *Silicon* **2022**, *14*, 8831–8843. https://doi.org/10.1007/s12633-022-01671-1.
34. Jiang, J.; Gunasekar, G.H.; Park, S.; Kim, S.H.; Yoon, S.; Piao, L. Hierarchical Cu nanoparticle-aggregated cages with high catalytic activity for reduction of 4-nitrophenol and carbon dioxide. *Mater. Res. Bull.* **2018**, *100*, 184–190. https://doi.org/10.1016/j.materresbull.2017.12.018.
35. Badri, A.; Slimi, S.; Guergueb, M.; Kahri, H.; Mateos, X. Green synthesis of copper oxide nanoparticles using Prickly Pear peel fruit extract: Characterization and catalytic activity. *Inorg. Chem. Commun.* **2021**, *134*, 109027. https://doi.org/10.1016/j.inoche.2021.109027.
36. Belkaid, N.; Boukoussa, B.; Mokhtar, A.; Hachemaoui, M.; Beldjilali, M.; Mekki, A.; Hacini, S.; Bengueddach, A.; Hamacha, R. Antibacterial Activity and Catalytic Reduction of 4-Nitrophenol and Methylene Blue on MCM-41 Modified by CuNPs. *Silicon* **2022**, *14*, 8505–8516. https://doi.org/10.1007/s12633-021-01642-y.
37. Maslamani, N.; Khan, S.B.; Danish, E.Y.; Bakhsh, E.M.; Akhtar, K.; Asiri, A.M. Metal nanoparticles supported chitosan coated carboxymethyl cellulose beads as a catalyst for the selective removal of 4-nitrophenol. *Chemosphere* **2022**, *291*, 133010. https://doi.org/10.1016/j.chemosphere.2021.133010.
38. Krishnan, S.K.; Esparza, R.; Uribe, D.B.; Mukherjee, S.; Pal, U. Facile seed-mediated growth of ultrathin AuCu shells on pd nanocubes and their enhanced nitrophenol degradation reactions. *J. Phys. Chem. C* **2021**, *125*, 13759–13769. https://doi.org/10.1021/acs.jpcc.1c00646.
39. Konar, S.; Kalita, H.; Puvvada, N.; Tantubay, S.; Mahto, M.K.; Biswas, S.; Pathak, A. Shape-dependent catalytic activity of CuO nanostructures. *J. Catal.* **2016**, *336*, 11–22. https://doi.org/10.1016/j.jcat.2015.12.017.
40. Rout, L.; Kumar, A.; Dhaka, R.S.; Reddy, G.N.; Giri, S.; Dash, P. Bimetallic Au-Cu alloy nanoparticles on reduced graphene oxide support: Synthesis, catalytic activity and investigation of synergistic effect by DFT analysis. *Applied Catalysis A: General* **2017**, *538*, 107–122. https://doi.org/10.1016/j.apcata.2017.03.017.
41. Jiang, J.; Lim, Y.S.; Park, S.; Kim, S.H.; Yoon, S.; Piao, L. Hollow porous Cu particles from silica-encapsulated Cu_2O nanoparticle aggregates effectively catalyze 4-nitrophenol reduction. *Nanoscale* **2017**, *9*, 3873–3880. https://doi.org/10.1039/c6nr09934c.
42. Sun, Y.; Zhang, F.; Xu, L.; Yin, Z.; Song, X. Roughness-controlled copper nanowires and Cu nanowires–Ag heterostructures: synthesis and their enhanced catalysis. *J. Mater. Chem. A* **2014**, *2*, 18583–18592. https://doi.org/10.1039/c4ta03689a.
43. Anantharamaiah, P.N.; Mondal, S.; Saha, S. Inducing the Catalytic Activity in SrFe12O19 Via Chemical Modification. *Catalysis Letters* **2020**, *151*, 221–231. https://doi.org/10.1007/s10562-020-03292-y.
44. Aditya, T.; Jana, J.; Singh, N.K.; Pal, A.; Pal, T. Remarkable Facet Selective Reduction of 4-Nitrophenol by Morphologically Tailored (111) Faceted Cu_2O Nanocatalyst. *ACS Omega* **2017**, *2*, 1968–1984. https://doi.org/10.1021/acsomega.6b00447.
45. Zhang, Y.; Zhu, P.; Chen, L.; Li, G.; Zhou, F.; Lu, D.D.; Sun, R.; Zhou, F.; ping Wong, C. Hierarchical architectures of monodisperse porous Cu microspheres: synthesis, growth mechanism, high-efficiency and recyclable catalytic performance. *Journal of Materials Chemistry A* **2014**, *2*, 11966. https://doi.org/10.1039/c4ta01920b.
46. Mandlimath, T.R.; Gopal, B. Catalytic activity of first row transition metal oxides in the conversion of p-nitrophenol to p-aminophenol. *Journal of Molecular Catalysis A: Chemical* **2011**, *350*, 9–15. https://doi.org/10.1016/j.molcata.2011.08.009.
47. Gao, S.; Jia, X.; Yang, J.; Wei, X. Hierarchically micro/nanostructured porous metallic copper: Convenient growth and superhydrophilic and catalytic performance. *J. Mater. Chem.* **2012**, *22*, 21733. https://doi.org/10.1039/c2jm35233h.

48. Zhang, P.; Sui, Y.; Xiao, G.; Wang, Y.; Wang, C.; Liu, B.; Zou, G.; Zou, B. Facile fabrication of faceted copper nanocrystals with high catalytic activity for p-nitrophenol reduction. *J. Mater. Chem. A* **2013**, *1*, 1632–1638. https://doi.org/10.1039/c2ta00350c.
49. Sun, Y.; Xu, L.; Yin, Z.; Song, X. Synthesis of copper submicro/nanoplates with high stability and their recyclable superior catalytic activity towards 4-nitrophenol reduction. *J. Mater. Chem.A* **2013**, *1*, 12361. https://doi.org/10.1039/c3ta12526b.
50. Sasmal, A.K.; Dutta, S.; Pal, T. A ternary Cu_2O-Cu-CuO nanocomposite: a catalyst with intriguing activity. *Dalton Trans.* **2016**, *45*, 3139–3150. https://doi.org/10.1039/c5dt03859f.
51. Bakhsh, E.M.; Ismail, M.; Sharafat, U.; Akhtar, K.; Fagieh, T.M.; Danish, E.Y.; Khan, S.B.; Khan, M.I.; Khan, M.A.; Asiri, A.M. Highly efficient and recoverable Ag-Cu bimetallic catalyst supported on taro-rhizome powder applied for nitroarenes and dyes reduction. *Journal of Materials Research and Technology* **2022**, *18*, 769–787. https://doi.org/10.1016/j.jmrt.2022.02.062.
52. Zhao, Z.; Xiao, Z.; Qin, C.; Lv, H.; Qin, L.; Niu, W.; Zhai, S.; An, Q. Sandwich-like N-C/Cu/N-C porous beads derived from alginate with enhanced catalytic activity and excellent recyclability for 4-nitrophenol reduction. *Industrial Crops and Products* **2021**, *164*, 113413. https://doi.org/10.1016/j.indcrop.2021.113413.
53. Li, M.; Su, Y.; Hu, J.; Geng, H.; Wei, H.; Yang, Z.; Zhang, Y. Hydrothermal synthesis of porous copper microspheres towards efficient 4-nitrophenol reduction. *Mater. Res. Bull.* **2016**, *83*, 329–335. https://doi.org/10.1016/j.materresbull.2016.04.022.
54. Sun, L.; Deng, Y.; Yang, Y.; Xu, Z.; Xie, K.; Liao, L. Preparation and catalytic activity of magnetic bimetallic nickel/copper nanowires. *RSC Advances* **2017**, *7*, 17781–17787. https://doi.org/10.1039/c7ra01068k.
55. Shah, S.A.; Ahmad, Z.; Khan, S.A.; Al-Ghamdi, Y.O.; Bakhsh, E.M.; Khan, N.; ur Rehman, M.; Jabli, M.; Khan, S.B. Biomass impregnated zero-valent Ag and Cu supported-catalyst: Evaluation in the reduction of nitrophenol and discoloration of dyes in aqueous medium. *Journal of Organometallic Chemistry* **2021**, *938*, 121756. https://doi.org/10.1016/j.jorganchem.2021.121756.
56. Kamal, T.; Asiri, A.M.; Ali, N. Catalytic reduction of 4-nitrophenol and methylene blue pollutants in water by copper and nickel nanoparticles decorated polymer sponges. *Spectrochimica Acta Part A: Molecular and Biomolecular Spectroscopy* **2021**, *261*, 120019. https://doi.org/10.1016/j.saa.2021.120019.
57. Fagieh, T.M.; Bakhsh, E.M.; Khan, S.B.; Akhtar, K.; Asiri, A.M. Alginate/Banana Waste Beads Supported Metal Nanoparticles for Efficient Water Remediation. *Polymers* **2021**, *13*, 4054. https://doi.org/10.3390/polym13234054.
58. Khan, N.; Shahida, B.; Khan, S.A.; Ahmad, Z.; Saeeduddin.; Sheikh, Z.; Bakhsh, E.M.; Alraddadi, H.M.; Fagieh, T.M.; Khan, S.B. Anchoring Zero-Valent Cu and Ni Nanoparticles on Carboxymethyl Cellulose-Polystyrene–Block Polyisoprene–Block Polystyrene Composite Films for Nitrophenol Reduction and Dyes Degradation. *Journal of Polymers and the Environment* **2022**, *31*, 608–620. https://doi.org/10.1007/s10924-022-02579-y.
59. Ali, H.S.H.M.; Anwar, Y.; Khan, S.A. Vigna radiata Impregnated Zero-Valent CuAg NPs: Applications in Nitrophenols Reduction, Dyes Discoloration and Antibacterial Activity. *Journal of Cluster Science* **2021**, *33*, 1407–1416. https://doi.org/10.1007/s10876-021-02067-8.
60. Deka, P.; Deka, R.C.; Bharali, P. In situ generated copper nanoparticle catalyzed reduction of 4-nitrophenol. *New J. Chem.* **2014**, *38*, 1789. https://doi.org/10.1039/c3nj01589k.
61. Khan, S.A.; Bakhsh, E.M.; Asiri, A.M.; Khan, S.B. Chitosan coated NiAl layered double hydroxide microsphere templated zero-valent metal NPs for environmental remediation. *Journal of Cleaner Production* **2021**, *285*, 124830. https://doi.org/10.1016/j.jclepro.2020.124830.
62. Locatelli, P.P.; Gurtat, M.; Lenz, G.F.; Marroquin, J.F.R.; Felix, J.F.; Schneider, R.; Borba, C.E. Simple borophosphate glasses for on-demand growth of self-supported copper nanoparticles in the reduction of 4-nitrophenol. *Journal of Hazardous Materials* **2021**, *416*, 125801. https://doi.org/10.1016/j.jhazmat.2021.125801.
63. Singh, V.K.; Kumar, K.; Das, A.; Tiwari, R.; Krishnamoorthi, S. Ameliorated microgel for bimetallic Ag/CuO nanoparticles and their expeditious catalytic applications. *Iranian Polymer Journal* **2023**, *32*, 687–701. https://doi.org/10.1007/s13726-023-01155-y.
64. Bhatia, P.; Nath, M. Ag nanoparticles anchored on NiO octahedrons (Ag/NiO composite): An efficient catalyst for reduction of nitro substituted phenols and colouring dyes. *Chemosphere* **2022**, *290*, 133188. https://doi.org/10.1016/j.chemosphere.2021.133188.
65. Rani, P.; Kumar, V.; Singh, P.P.; Matharu, A.S.; Zhang, W.; Kim, K.H.; Singh, J.; Rawat, M. Highly stable AgNPs prepared via a novel green approach for catalytic and photocatalytic removal of biological and non-biological pollutants. *Environ. Int.* **2020**, *143*, 105924. https://doi.org/10.1016/j.envint.2020.105924.
66. Song, Y.; Jiang, H.; Wang, B.; Kong, Y.; Chen, J. Silver-incorporated mussel-inspired polydopamine coatings on mesoporous silica as an efficient nanocatalyst and antimicrobial agent. *ACS Appl. Mater. Interfaces* **2018**, *10*, 1792–1801. https://doi.org/10.1021/acsami.7b18136.
67. Kästner, C.; Thünemann, A.F. Catalytic Reduction of 4-Nitrophenol Using Silver Nanoparticles with Adjustable Activity. *Langmuir* **2016**, *32*, 7383–7391. https://doi.org/10.1021/acs.langmuir.6b01477.
68. Jiang, S.; Wang, L.; Duan, Y.; An, J.; Luo, Q.; Zhang, Y.; Tang, Y.; Huang, J.; Zhang, B.; Liu, J.; et al. A novel strategy to construct supported silver nanocomposite as an ultra-high efficient catalyst. *Appl. Catal., B* **2021**, *283*, 119592. https://doi.org/10.1016/j.apcatb.2020.119592.
69. Xu, Y.; Zhou, F.; Chen, M.; Hu, H.; Lin, L.; Wu, J.; Zhang, M. Facile assembly of 2D α-zirconium phosphate supported silver nanoparticles: superior and recyclable catalysis. *New J. Chem.* **2020**, *44*, 9793–9801. https://doi.org/10.1039/d0nj01378a.
70. Meng, L.; Liu, Z.; Lan, C.; Xu, N. In-situ fabricating ag nanoparticles on TiO_2 for unprecedented high catalytic activity of 4-nitrophenol reduction. *Catal. Lett.* **2021**, *152*, 912–920. https://doi.org/10.1007/s10562-021-03671-z.

71. Jeyapriya, M.; Meenarathi, B.; Tung, K.L.; Anbarasan, R. Characterization and applications of amino acid-bridged nano-Ag end-capped diblock copolymer. *Iran. Polym. J.* **2019**, *29*, 77–90. https://doi.org/10.1007/s13726-019-00776-6.
72. Lu, Q.; An, J.; Duan, Y.; Luo, Q.; Yin, R.; Li, X.; Tang, C.; Wang, D. A strategy for preparing efficient Ag/p-BNNS nanocatalyst with a synergistic effect between Ag and p-BNNS. *J. Catal.* **2021**, *395*, 457–466. https://doi.org/10.1016/j.jcat.2021.02.004.
73. Qi, Y.; Ye, J.; Ren, S.; Lv, J.; Zhang, S.; Che, Y.; Ning, G. In-situ synthesis of metal nanoparticles@metal-organic frameworks: Highly effective catalytic performance and synergistic antimicrobial activity. *J. Hazard. Mater.* **2020**, *387*, 121687. https://doi.org/10.1016/j.jhazmat.2019.121687.
74. Kist, J.A.; Essner, J.B.; Woodward, J.D.; Baker, G.A. Silver-mediated squaric acid reduction as a facile, ambient-temperature and seedless route to tunable bimetallic Au/Ag nanostars and nanosnowflakes. *ChemNanoMat* **2022**, *8*. https://doi.org/10.1002/cnma.202200189.
75. Yahagi, T.; Togashi, T.; Kurihara, M. An exclusive deposition method of silver nanoparticles on TiO_2 particles via low-temperature decomposition of silver-alkyldiamine complexes in aqueous media. *RSC Adv.* **2020**, *10*, 4545–4553. https://doi.org/10.1039/c9ra10307d.
76. Peng, C.; Kuai, Z.; Li, X.; Lian, S.; Jiang, D.; Tang, J.; Li, L.; Wu, R.; Wu, A.; Chen, S. Facile synthesis of Ag nanoparticles/$Ti_3C_2T_x$/polyacrylamide composite hydrogel as efficient catalyst for methylene blue and 4-nitrophenol reduction. *Mater. Design* **2021**, *210*, 110061. https://doi.org/10.1016/j.matdes.2021.110061.
77. Horta-Fraijo, P.; Smolentseva, E.; Simakov, A.; José-Yacaman, M.; Acosta, B. Ag nanoparticles in A4 zeolite as efficient catalysts for the 4-nitrophenol reduction. *Microporous Mesoporous Mater.* **2021**, *312*, 110707. https://doi.org/10.1016/j.micromeso.2020.110707.
78. Budi, C.S.; Deka, J.R.; Saikia, D.; Kao, H.M.; Yang, Y.C. Ultrafine bimetallic Ag-doped Ni nanoparticles embedded in cage-type mesoporous silica SBA-16 as superior catalysts for conversion of toxic nitroaromatic compounds. *J. Hazard. Mater.* **2020**, *384*, 121270. https://doi.org/10.1016/j.jhazmat.2019.121270.
79. Chen, C.S.; Chen, T.C.; Chiu, K.L.; Wu, H.C.; Pao, C.W.; Chen, C.L.; Hsu, H.C.; Kao, H.M. Silver particles deposited onto magnetic carbon nanofibers as highly active catalysts for 4-nitrophenol reduction. *Appl. Catal., B* **2022**, *315*, 121596. https://doi.org/10.1016/j.apcatb.2022.121596.
80. Varshney, S.; Bar-Ziv, R.; Zidki, T. On the remarkable performance of silver-based alloy nanoparticles in 4-nitrophenol catalytic reduction. *ChemCatChem* **2020**, *12*, 4680–4688. https://doi.org/10.1002/cctc.202000584.
81. Huo, H.; Jiang, Y.; Zhao, T.; Wang, Z.; Hu, Y.; Xu, X.; Lin, K. Quantitatively loaded ultra-small Ag nanoparticles on molecularly imprinted mesoporous silica for highly efficient catalytic reduction process. *J. Mater. Sci.* **2019**, *55*, 1475–1488. https://doi.org/10.1007/s10853-019-04054-x.
82. Malik, A.; Nath, M. Synthesis of Ag/ZIF-7 by immobilization of Ag nanoparticles onto ZIF-7 microcrystals: A heterogeneous catalyst for the reduction of nitroaromatic compounds and organic dyes. *J. Environ. Chem. Eng.* **2020**, *8*, 104547. https://doi.org/10.1016/j.jece.2020.104547.
83. Woo, H.; Lee, K.; Park, S.; Park, K. Magnetically separable and recyclable Fe_3O_4-supported ag nanocatalysts for reduction of nitro compounds and selective hydration of nitriles to amides in water. *Molecules* **2014**, *19*, 699–712. https://doi.org/10.3390/molecules19010699.
84. García-Valdivieso, G.; Arenas-Sánchez, E.; Horta-Fraijo, P.; Simakov, A.; Navarro-Contreras, H.R.; Acosta, B. Ag@ZnO/MWCNT ternary nanocomposite as an active and stable catalyst for the 4-nitrophenol reduction in water. *Nanotechnology* **2021**, *32*, 315713. https://doi.org/10.1088/1361-6528/abf96b.
85. Han, X.W.; Pan, H.; liu, M. In situ construction of reduced graphene oxide supported Ag nanoneedles heterogenous nanostructures with superior catalytic activity for 4-nitrophenol. *Colloids and Surfaces A: Physicochemical and Engineering Aspects* **2020**, *600*, 124929. https://doi.org/10.1016/j.colsurfa.2020.124929.
86. Xu, P.; Wu, Z.; Dai, W.; Wang, Y.; Zheng, M.; Su, X.; Teng, Z. Synthesis of multiple Ag nanoparticles loaded hollow mesoporous carbon spheres for highly efficient and recyclable catalysis. *Microporous Mesoporous Mater.* **2021**, *314*, 110856. https://doi.org/10.1016/j.micromeso.2020.110856.
87. Dsouza, A.; Shilpa, M.P.; Gurumurthy, S.C.; Nagaraja, B.S.; Mundinamani, S.; Ramam, K.; Gedda, M.; Murari, M.S. CuAg and AuAg bimetallic nanoparticles for catalytic and heat transfer applications. *Clean Technol. Environ. Policy* **2021**, *23*, 2145–2155. https://doi.org/10.1007/s10098-021-02120-0.
88. Yan, Z.; Fu, L.; Zuo, X.; Yang, H. Green assembly of stable and uniform silver nanoparticles on 2D silica nanosheets for catalytic reduction of 4-nitrophenol. *Appl. Catal., B* **2018**, *226*, 23–30. https://doi.org/10.1016/j.apcatb.2017.12.040.
89. Deshmukh, A.R.; Aloui, H.; Kim, B.S. In situ growth of gold and silver nanoparticles onto phyto-functionalized boron nitride nanosheets: Catalytic, peroxidase mimicking, and antimicrobial activity. *J. Cleaner Prod.* **2020**, *270*, 122339. https://doi.org/10.1016/j.jclepro.2020.122339.
90. Taratayko, A.; Larichev, Y.; Zaikovskii, V.; Mikheeva, N.; Mamontov, G. Ag–CeO2/SBA-15 composite prepared from Pluronic P123@SBA-15 hybrid as catalyst for room-temperature reduction of 4-nitrophenol. *Catal. Today* **2021**, *375*, 576–584. https://doi.org/10.1016/j.cattod.2020.05.001.
91. Maity, N.; Sahoo, A.; Boddhula, R.; Chatterjee, S.; Patra, S.; Panda, B.B. Fly ash supported Pd–Ag bimetallic nanoparticles exhibiting a synergistic catalytic effect for the reduction of nitrophenol. *Dalton Trans.* **2020**, *49*, 11019–11026. https://doi.org/10.1039/d0dt01899f.

92. Das, T.K.; Remanan, S.; Ghosh, S.; Das, N.C. An environment friendly free-standing cellulose membrane derived for catalytic reduction of 4-nitrophenol: A sustainable approach. *J. Environ. Chem. Eng.* **2021**, *9*, 104596. https://doi.org/10.1016/j.jece.2020.104596.
93. Zhang, S.; Jiang, W.; Liu, G.; Liu, S.; Chen, H.; Lyu, G.; Yang, G.; Liu, Y.; Ni, Y. Preparation of ultrafine and highly loaded silver nanoparticle composites and their highly efficient applications as reductive catalysts and antibacterial agents. *J. Colloid Interface Sci.* **2023**, *629*, 766–777. https://doi.org/10.1016/j.jcis.2022.09.018.
94. Lee, S.J.; Begildayeva, T.; Yeon, S.; Naik, S.S.; Ryu, H.; Kim, T.H.; Choi, M.Y. Eco-friendly synthesis of lignin mediated silver nanoparticles as a selective sensor and their catalytic removal of aromatic toxic nitro compounds. *Environ. Pollut.* **2021**, *269*, 116174. https://doi.org/10.1016/j.envpol.2020.116174.
95. Wang, L.; Chen, X.; Duan, Y.; Luo, Q.; Wang, D. Macroporous polymer resin with conjugated side-chains: an efficient Ag nanoparticle support for preparing a photocatalyst. *Catal. Sci. Technol.* **2020**, *10*, 4191–4200. https://doi.org/10.1039/d0cy00435a.
96. Bahadorikhalili, S.; Arshadi, H.; Afrouzandeh, Z.; Ma'mani, L. Ultrasonic promoted synthesis of Ag nanoparticle decorated thiourea-functionalized magnetic hydroxyapatite: a robust inorganic–organic hybrid nanocatalyst for oxidation and reduction reactions. *New J. Chem.* **2020**, *44*, 8840–8848. https://doi.org/10.1039/d0nj00829j.
97. Aazza, M.; Ahlafi, H.; Moussout, H.; Mounir, C.; Fadel, A.; Addad, A. Catalytic reduction of nitro-phenolic compounds over Ag, Ni and Co nanoparticles catalysts supported on γ-Al2O3. *Journal of Environmental Chemical Engineering* **2020**, *8*, 103707. https://doi.org/10.1016/j.jece.2020.103707.
98. Tran, X.T.; Hussain, M.; Kim, H.T. Facile and fast synthesis of a reduced graphene oxide/carbon nanotube/iron/silver hybrid and its enhanced performance in catalytic reduction of 4–nitrophenol. *Solid State Sci.* **2020**, *100*, 106107. https://doi.org/10.1016/j.solidstatesciences.2019.106107.
99. Zhang, Z.; Shao, C.; Sun, Y.; Mu, J.; Zhang, M.; Zhang, P.; Guo, Z.; Liang, P.; Wang, C.; Liu, Y. Tubular nanocomposite catalysts based on size-controlled and highly dispersed silver nanoparticles assembled on electrospun silicananotubes for catalytic reduction of 4-nitrophenol. *J. Mater. Chem.* **2012**, *22*, 1387–1395. https://doi.org/10.1039/c1jm13421c.
100. Ji, X.Y.; Yu, F.Y.; Li, Y.Q.; Zhu, H.T.; Zhao, H.Y.; Shi, Y.; Wang, Y.H.; Tan, H.Q.; Li, Y.G. Two-dimensional ultrathin surfactant-encapsulating polyoxometalate assemblies as carriers for monodispersing noble-metal nanoparticles with high catalytic activity and stability. *Dalton Trans.* **2021**, *50*, 1666–1671. https://doi.org/10.1039/d0dt03976d.
101. Gondwal, M.; Sharma, N.; nee Pant, G.J.; Gautam, B.P.S.; Singh, S.; Tumba, K.; Bahadur, I. Bioactivity and Catalytic Reduction of Aryl Nitro-Compounds by Biosynthesized Silver Nanoparticles using Skimmiaanquetilia. *ChemistrySelect* **2023**, *8*. https://doi.org/10.1002/slct.202203782.
102. Ma, J.; Zhang, S.; Liu, L.; Zhang, C.; Shen, C.; Zhou, J.; Cheng, H.; Ge, Y.; Tong, Z.; Chen, Z.; et al. Immobilization of well-dispersed Ag nanoparticles on calcium niobate nanosheets as highly active catalyst towards reduction of 4-nitrophenol. *J. Taiwan Inst. Chem. Eng.* **2020**, *110*, 92–99. https://doi.org/10.1016/j.jtice.2020.02.011.
103. Supriya.; Pal, C.K.; Sengupta, S.; Basu, J.K. One pot synthesis of nano Ag in calcium alginate beads and its catalytic application in p-Nitrophenol reduction with kinetic parameter estimation and model fitting. *The Canadian Journal of Chemical Engineering* **2020**, *99*, 359–373. https://doi.org/10.1002/cjce.23831.
104. Keshri, K.S.; Chowdhury, B. Effect of the Ag-CeO_2 interaction and the nature of pore structure on the catalytic activities of different Ag-CeO_2/mesoporous-SiO_2 catalysts on the reduction of 4-nitrophenol. *J. Porous Mater.* **2022**, *29*, 893–906. https://doi.org/10.1007/s10934-022-01222-4.
105. Sachi.; Singh, A.P.; Thirumal, M. Fabrication of AgNi nano-alloy-decorated ZnO nanocomposites as an efficient and novel hybrid catalyst to degrade noxious organic pollutants. *ACS Omega* **2021**, *6*, 34771–34782. https://doi.org/10.1021/acsomega.1c05266.
106. Chishti, A.N.; Ma, Z.; Liu, Y.; Chen, M.; Gautam, J.; Guo, F.; Ni, L.; Diao, G. Synthesis of highly efficient and magnetically separable Fe_3O_4@C-TiO_2-Ag catalyst for the reduction of organic dyes and 4-nitrophenol. *Colloids Surf., A* **2021**, *631*, 127694. https://doi.org/10.1016/j.colsurfa.2021.127694.
107. Yang, Q.; Li, L.; Zhao, F.; Wang, Y.; Ye, Z.; Hua, C.; Liu, Z.; Bohinc, K.; Guo, X. Spherical polyelectrolyte brushes as templates to prepare hollow silica spheres encapsulating metal nanoparticles. *Nanomaterials* **2020**, *10*, 799. https://doi.org/10.3390/nano10040799.
108. Jiang, K.; Zhang, H.X.; Yang, Y.Y.; Mothes, R.; Lang, H.; Cai, W.B. Facile synthesis of Ag@Pd satellites-Fe_3O_4 core nanocomposites as efficient and reusable hydrogenation catalysts. *Chem. Commun.* **2011**, *47*, 11924. https://doi.org/10.1039/c1cc14675k.
109. Heidari, H.; Aliramezani, F. Reductant-free and in-situ green synthesis of ag nanoparticles on Fe_3O_4@nanocellulose and their catalytic activity for the reduction of dyes. *ChemistrySelect* **2021**, *6*, 1223–1229. https://doi.org/10.1002/slct.202004579.
110. Wang, M.; Yuan, B.; Bai, S. Preparation of Ag/C fiber with nanostructure through in situ thermally induced redox reaction between PVA and $AgNO_3$ and its catalysis for 4-nitrophenol reduction. *Polym. Adv. Technol.* **2020**, *31*, 1312–1320. https://doi.org/10.1002/pat.4860.
111. Chen, S.; Wang, G.; Pang, T.; Sui, W.; Chen, Z.; Si, C. Green assembly of high-density and small-sized silver nanoparticles on lignosulfonate-phenolic resin spheres: Focusing on multifunction of lignosulfonate. *Int. J. Biol. Macromol.* **2021**, *166*, 893–901. https://doi.org/10.1016/j.ijbiomac.2020.10.246.
112. Cao, T.; Wang, C.; Zhou, Z.; Liu, L.; Xu, S.; Song, H.; Lin, W.; Xu, Z. Magnetic multi-functional SBA-15 supported silver nanocomposites: Synthesis, characterization and application. *Appl. Surf. Sci.* **2021**, *552*, 149487. https://doi.org/10.1016/j.apsusc.2021.149487.

113. Zarei, M.; Seyedi, N.; Maghsoudi, S.; Nejad, M.S.; Sheibani, H. Green synthesis of Ag nanoparticles on the modified graphene oxide using Capparis spinosa fruit extract for catalytic reduction of organic dyes. *Inorg. Chem. Commun.* **2021**, *123*, 108327. https://doi.org/10.1016/j.inoche.2020.108327.
114. Chernykh, M.; Mikheeva, N.; Zaikovskii, V.; Salaev, M.; Liotta, L.F.; Mamontov, G. Room-Temperature Nitrophenol Reduction over Ag–CeO2 Catalysts: The Role of Catalyst Preparation Method. *Catalysts* **2020**, *10*, 580. https://doi.org/10.3390/catal10050580.
115. Veeramani, V.; Chi, N.V.; Yang, Y.L.; Huong, N.T.H.; Tran, T.V.; Ahamad, T.; Alshehri, S.M.; Wu, K.C.W. Decoration of silver nanoparticles on nitrogen-doped nanoporous carbon derived from zeolitic imidazole framework-8 (ZIF-8) via in situ auto-reduction. *RSC Advances* **2021**, *11*, 6614–6619. https://doi.org/10.1039/d0ra10546e.
116. Gao, C.; Xiao, L.; Zhou, J.; Wang, H.; Zhai, S.; An, Q. Immobilization of nanosilver onto glycine modified lignin hydrogel composites for highly efficient p-nitrophenol hydrogenation. *Chemical Engineering Journal* **2021**, *403*, 126370. https://doi.org/10.1016/j.cej.2020.126370.
117. Shi, X.; Huang, C.; Zheng, Z.; Zhong, B.; Ding, G.; Li, J.; You, L.; Wang, S. Preparation of magnetically recoverable MPCTP-Ag composite nanoparticles and their application as high-performance catalysts. *Langmuir* **2021**, *37*, 10249–10258. https://doi.org/10.1021/acs.langmuir.1c00944.
118. Wang, Y.; Gao, P.; Wei, Y.; Jin, Y.; Sun, S.; Wang, Z.; Jiang, Y. Silver nanoparticles decorated magnetic polymer composites (Fe_3O_4@PS@Ag) as highly efficient reusable catalyst for the degradation of 4-nitrophenol and organic dyes. *J. Environ. Manage.* **2021**, *278*, 111473. https://doi.org/10.1016/j.jenvman.2020.111473.
119. Lu, Y.; Mao, J.; Wang, Z.; Qin, Y.; Zhou, J. Facile synthesis of porous hexapod Ag@AgCl dual catalysts for in situ SERS monitoring of 4-nitrothiophenol reduction. *Catalysts* **2020**, *10*, 746. https://doi.org/10.3390/catal10070746.
120. Alshehri, S.M.; Almuqati, T.; Almuqati, N.; Al-Farraj, E.; Alhokbany, N.; Ahamad, T. Chitosan based polymer matrix with silver nanoparticles decorated multiwalled carbon nanotubes for catalytic reduction of 4-nitrophenol. *Carbohydr. Polym.* **2016**, *151*, 135–143. https://doi.org/10.1016/j.carbpol.2016.05.018.
121. Rocha, M.; Pereira, C.; Freire, C. Au/Ag nanoparticles-decorated TiO_2 with enhanced catalytic activity for nitroarenes reduction. *Colloids Surf., A* **2021**, *621*, 126614. https://doi.org/10.1016/j.colsurfa.2021.126614.
122. Lee, Y.J.; Park, Y. Graphene oxide grafted gold nanoparticles and silver/silver chloride nanoparticles green-synthesized by a Portulaca oleracea extract: Assessment of catalytic activity. *Colloids and Surfaces A: Physicochemical and Engineering Aspects* **2020**, *607*, 125527. https://doi.org/10.1016/j.colsurfa.2020.125527.
123. Heidari, H.; Karbalaee, M. Silver-nanoparticle supported on nanocrystalline cellulose using cetyltrimethylammonium bromide: synthesis and catalytic performance for decolorization of dyes. *Journal of Nanostructures* **2021**, *11*. https://doi.org/10.22052/JNS.2021.01.006.
124. Liang, M.; Su, R.; Huang, R.; Qi, W.; Yu, Y.; Wang, L.; He, Z. Facile in situ synthesis of silver nanoparticles on procyanidin-grafted eggshell membrane and their catalytic properties. *ACS Appl. Mater. Interfaces* **2014**, *6*, 4638–4649. https://doi.org/10.1021/am500665p.
125. Erdem, H.B.; Çetinkaya, S. Facile insitu preparation of silver nanoparticles supported on petroleum asphaltene-derived porous carbon for efficient reduction of nitrophenols. *Heliyon* **2022**, *8*, e10659. https://doi.org/10.1016/j.heliyon.2022.e10659.
126. He, L.; Zhou, Z.; Liu, Z.; Nan, X.; Wang, T.; Sun, X.; Bai, P. Morphology design and synthesis of magnetic microspheres as highly efficient reusable catalyst for organic dyes. *Colloids Surf., A* **2023**, *656*, 130542. https://doi.org/10.1016/j.colsurfa.2022.130542.
127. Hoang, T.T.T.L.; Insin, N.; Sukpirom, N. Catalytic activity of silver nanoparticles anchored on layered double hydroxides and hydroxyapatite. *Inorg. Chem. Commun.* **2020**, *121*, 108199. https://doi.org/10.1016/j.inoche.2020.108199.
128. Kottappara, R.; Palantavida, S.; Vijayan, B.K. A facile synthesis of Cu–CuO–Ag nanocomposite and their hydrogenation reduction of p-nitrophenol. *SN Applied Sciences* **2020**, *2*. https://doi.org/10.1007/s42452-020-03386-7.
129. Doan, V.D.; Phan, T.L.; Le, V.T.; Vasseghian, Y.; Evgenievna, L.O.; Tran, D.L.; Le, V.T. Efficient and fast degradation of 4-nitrophenol and detection of Fe(III) ions by Poria cocos extract stabilized silver nanoparticles. *Chemosphere* **2022**, *286*, 131894. https://doi.org/10.1016/j.chemosphere.2021.131894.
130. Skiba, M.I.; Vorobyova, V.I.; Kosogina, I.V. Preparation of Silver Nanoparticles in a Plasma-Liquid System in the Presence of PVA: Antimicrobial, Catalytic, and Sensing Properties. *Journal of Chemistry* **2020**, *2020*, 1–9. https://doi.org/10.1155/2020/5380950.
131. Żółtowska, S.; Modelska, M.; Piasecki, A.; Jesionowski, T. Commercial sponges in heterogeneous catalysis: developing novel composites with cobalt and silver. *Physicochem. Probl. Mi.* **2020**, pp. 89–100. https://doi.org/10.37190/ppmp/126866.
132. Islam, M.R.; Ferdous, M.; Sujan, M.I.; Mao, X.; Zeng, H.; Azam, M.S. Recyclable Ag-decorated highly carbonaceous magnetic nanocomposites for the removal of organic pollutants. *J. Colloid Interface Sci.* **2020**, *562*, 52–62. https://doi.org/10.1016/j.jcis.2019.11.119.
133. Nguyen, T.M.T.; Huynh, T.T.T.; Dang, C.H.; Mai, D.T.; Nguyen, T.T.N.; Nguyen, D.T.; Dang, V.S.; Nguyen, T.D.; Nguyen, T.D. Novel biogenic silver nanoparticles used for antibacterial effect and catalytic degradation of contaminants. *Res. Chem. Intermed.* **2020**, *46*, 1975–1990. https://doi.org/10.1007/s11164-019-04075-w.
134. Upoma, B.P.; Mahnaz, F.; Sajal, W.R.; Zahan, N.; Firoz, M.S.H.; Azam, M.S. Bio-inspired immobilization of silver and gold on magnetic graphene oxide for rapid catalysis and recyclability. *J. Environ. Chem. Eng.* **2020**, *8*, 103739. https://doi.org/10.1016/j.jece.2020.103739.

135. Cao, H.L.; Liu, C.; Cai, F.Y.; Qiao, X.X.; Dichiara, A.B.; Tian, C.; Lü, J. In situ immobilization of ultra-fine Ag NPs onto magnetic Ag@RF@Fe_3O_4 core-satellite nanocomposites for the rapid catalytic reduction of nitrophenols. *Water Res.* **2020**, *179*, 115882. https://doi.org/10.1016/j.watres.2020.115882.
136. Pang, Y.; Chen, Z.; Zhao, R.; Yi, C.; Qiu, X.; Qian, Y.; Lou, H. Facile synthesis of easily separated and reusable silver nanoparticles/aminated alkaline lignin composite and its catalytic ability. *J. Colloid Interface Sci.* **2021**, *587*, 334–346. https://doi.org/10.1016/j.jcis.2020.11.113.
137. Srećković, N.Z.; Nedić, Z.P.; Liberti, D.; Monti, D.M.; Mihailović, N.R.; Stanković, J.S.K.; Dimitrijević, S.; Mihailović, V.B. Application potential of biogenically synthesized silver nanoparticles using Lythrum salicaria L. extracts as pharmaceuticals and catalysts for organic pollutant degradation. *RSC Advances* **2021**, *11*, 35585–35599. https://doi.org/10.1039/d1ra05570d.
138. Zhou, A.; Li, J.; Wang, G.; Xu, Q. Preparation of Ag/ZrGP nanocomposites with enhanced catalytic activity for catalytic reduction of 4-nitrophenol. *Appl. Surf. Sci.* **2020**, *506*, 144570. https://doi.org/10.1016/j.apsusc.2019.144570.
139. Chishti, A.N.; Guo, F.; Aftab, A.; Ma, Z.; Liu, Y.; Chen, M.; Gautam, J.; Chen, C.; Ni, L.; Diao, G. Synthesis of silver doped Fe_3O_4/C nanoparticles and its catalytic activities for the degradation and reduction of methylene blue and 4-nitrophenol. *Appl. Surf. Sci.* **2021**, *546*, 149070. https://doi.org/10.1016/j.apsusc.2021.149070.
140. Le, V.T.; Ngu, N.N.Q.; Chau, T.P.; Nguyen, T.D.; Nguyen, V.T.; Nguyen, T.L.H.; Cao, X.T.; Doan, V.D. Silver and Gold Nanoparticles from Limnophila rugosa Leaves: Biosynthesis, Characterization, and Catalytic Activity in Reduction of Nitrophenols. *Journal of Nanomaterials* **2021**, *2021*, 1–11. https://doi.org/10.1155/2021/5571663.
141. Khan, S.B.; Ismail, M.; Bakhsh, E.M.; Asiri, A.M. Design of simple and efficient metal nanoparticles templated on ZnO-chitosan coated textile cotton towards the catalytic reduction of organic pollutants. *J. Ind. Text.* **2020**, *51*, 1703S–1728S. https://doi.org/10.1177/1528083720931481.
142. Karegar, M.; Khodaei, M.M. Magnetic polyindole-Ag composite for the catalytic reduction and removing of the organic pollutants. *Polym. Bull.* **2022**, *79*, 11431–11460. https://doi.org/10.1007/s00289-021-04043-8.
143. Deshmukh, A.R.; Dikshit, P.K.; Kim, B.S. Green in situ immobilization of gold and silver nanoparticles on bacterial nanocellulose film using Punica granatum peels extract and their application as reusable catalysts. *Int. J. Biol. Macromol.* **2022**, *205*, 169–177. https://doi.org/10.1016/j.ijbiomac.2022.02.064.
144. Vo, T.T.; Dang, C.H.; Doan, V.D.; Dang, V.S.; Nguyen, T.D. Biogenic Synthesis of Silver and Gold Nanoparticles from Lactuca indica Leaf Extract and Their Application in Catalytic Degradation of Toxic Compounds. *J. Inorg. Organomet. Polym. Mater.* **2019**, *30*, 388–399. https://doi.org/10.1007/s10904-019-01197-x.
145. Krishnan, R.R.; Chandran, S.R.; Johnson, E.; Raveendrakurup, R.; Hariharan, P.K. Bulk Level Synthesis of Solid Silver Nanocatalyst: Green Mediated Approach. *ChemistrySelect* **2022**, *7*. https://doi.org/10.1002/slct.202201554.
146. Doan, V.D.; Phung, M.T.; Nguyen, T.L.H.; Mai, T.C.; Nguyen, T.D. Noble metallic nanoparticles from waste Nypa fruticans fruit husk: Biosynthesis, characterization, antibacterial activity and recyclable catalysis. *Arabian J. Chem.* **2020**, *13*, 7490–7503. https://doi.org/10.1016/j.arabjc.2020.08.024.
147. Doan, V.D.; Le, V.T.; Phan, T.L.; Nguyen, T.L.H.; Nguyen, T.D. Waste banana stem utilized for biosynthesis of silver and gold nanoparticles and their antibacterial and catalytic properties. *J. Cluster Sci.* **2020**, *32*, 1673–1682. https://doi.org/10.1007/s10876-020-01930-4.
148. Liu, Y.Y.; Zhao, Y.H.; Zhou, Y.; Guo, X.L.; Chen, Z.T.; Zhang, W.J.; Zhang, Y.; Chen, J.; Wang, Z.M.; Sun, L.T.; et al. High-efficient catalytic reduction of 4-nitrophenol based on reusable Ag nanoparticles/graphene-loading loofah sponge hybrid. *Nanotechnology* **2018**, *29*, 315702. https://doi.org/10.1088/1361-6528/aac3e8.
149. Chishti, A.N.; Ni, L.; Guo, F.; Lin, X.; Liu, Y.; Wu, H.; Chen, M.; Diao, G.W. Magnetite-Silica core-shell nanocomposites decorated with silver nanoparticles for enhanced catalytic reduction of 4-nitrophenol and degradation of methylene blue dye in the water. *J. Environ. Chem. Eng.* **2021**, *9*, 104948. https://doi.org/10.1016/j.jece.2020.104948.
150. Gouyau, J.; Duval, R.E.; Boudier, A.; Lamouroux, E. Investigation of nanoparticle metallic core antibacterial activity: gold and silver nanoparticles against escherichia coli and staphylococcus aureus. *Int. J. Mol. Sci.* **2021**, *22*, 1905. https://doi.org/10.3390/ijms22041905.
151. Doan, V.D.; Huynh, B.A.; Nguyen, T.D.; Cao, X.T.; Nguyen, V.C.; Nguyen, T.L.H.; Nguyen, H.T.; Le, V.T. Biosynthesis of silver and gold nanoparticles using aqueous extract of codonopsis pilosula roots for antibacterial and catalytic applications. *J. Nanomater.* **2020**, *2020*, 1–18. https://doi.org/10.1155/2020/8492016.
152. Choi, Y.; Bae, H.S.; Seo, E.; Jang, S.; Park, K.H.; Kim, B.S. Hybrid gold nanoparticle-reduced graphene oxide nanosheets as active catalysts for highly efficient reduction of nitroarenes. *Journal of Materials Chemistry* **2011**, *21*, 15431. https://doi.org/10.1039/c1jm12477c.
153. Guo, S.; Yuan, H.; Luo, W.; Liu, X.; Zhang, X.; Jiang, H.; Liu, F.; Cheng, G.J. Isolated atomic catalysts encapsulated in MOF for ultrafast water pollutant treatment. *Nano Research* **2021**, *14*, 1287–1293. https://doi.org/10.1007/s12274-020-3138-5.
154. López-Cisneros, M.; Smolentseva, E.; Acosta, B.; Simakov, A. Synthesis by spray pyrolysis of gold nano species confined in iron oxide nanospheres effective in the reduction of 4-nitrophenol to 4-aminophenol. *Nanotechnology* **2021**, *32*, 425602. https://doi.org/10.1088/1361-6528/ac137c.
155. Yan, X.X.; Zhang, Y.F.; Chen, Y.; Wang, B. In-situ generated gold nanorods on surface layer of fibers: facile preparation and unprecedented high catalytic activities in reduction of 4-nitrophenol. *Colloids and Surfaces A: Physicochemical and Engineering Aspects* **2020**, *598*, 124826. https://doi.org/10.1016/j.colsurfa.2020.124826.

156. Seo, E.; Kim, J.; Hong, Y.; Kim, Y.S.; Lee, D.; Kim, B.S. Double hydrophilic block copolymer templated Au nanoparticles with enhanced catalytic activity toward nitroarene reduction. *J. Phys. Chem. C* **2013**, *117*, 11686–11693, [https://doi.org/10.1021/jp4027139]. https://doi.org/10.1021/jp4027139.
157. Sun, L.; Diao, Y.; Shan, Y.; Zhi, Y.; Zhang, H.; Dou, B.; Huang, W. Novel in-situ synthesis of metal nanoparticles-supported bulk photocatalyst and its application in 4-nitrophenol reduction. *Appl. Surf. Sci.* **2022**, *598*, 153791. https://doi.org/10.1016/j.apsusc.2022.153791.
158. Zhou, X.; Li, Y.; Xing, Y.; Liu, X.; Yu, X.; Yu, Y. Comparison of the catalytic properties of Au nanoparticles supported on different two-dimensional carriers. *Journal of Physics and Chemistry of Solids* **2020**, *142*, 109438. https://doi.org/10.1016/j.jpcs.2020.109438.
159. Kumar, L.; Singh, S.; Horechyy, A.; Formanek, P.; Hübner, R.; Albrecht, V.; Weißpflog, J.; Schwarz, S.; Puneet, P.; Nandan, B. Hollow Au@TiO_2 porous electrospun nanofibers for catalytic applications. *RSC Adv.* **2020**, *10*, 6592–6602. https://doi.org/10.1039/c9ra10487a.
160. Layek, K.; Kantam, M.L.; Shirai, M.; Nishio-Hamane, D.; Sasaki, T.; Maheswaran, H. Gold nanoparticles stabilized on nanocrystalline magnesium oxide as an active catalyst for reduction of nitroarenes in aqueous medium at room temperature. *Green Chemistry* **2012**, *14*, 3164. https://doi.org/10.1039/c2gc35917k.
161. Lamei, K.; Eshghi, H.; Bakavoli, M.; Rostamnia, S. Magnetically Recoverable Gold Nanorods as a Novel Catalyst for the Facile Reduction of Nitroarenes Under Aqueous Conditions. *Catalysis Letters* **2016**, *147*, 491–501. https://doi.org/10.1007/s10562-016-1921-4.
162. Chen, Z.; Huang, Q.; Zhang, Y.; Sheng, P.; Cui, Z. Confined generation of homogeneously dispersed Au and SnO_2 nanoparticles in layered silicate as synergistic catalysts. *Langmuir* **2021**, *37*, 2341–2348. https://doi.org/10.1021/acs.langmuir.0c03216.
163. Wang, J.; Cai, C.; Zhang, Z.; Li, C.; Liu, R. Electrospun metal-organic frameworks with polyacrylonitrile as precursors to hierarchical porous carbon and composite nanofibers for adsorption and catalysis. *Chemosphere* **2020**, *239*, 124833. https://doi.org/10.1016/j.chemosphere.2019.124833.
164. Meng, J.; Ma, H.; Shu, J.; Li, R.; Jin, R.; Li, S.; Yang, H. Gold nanoparticles self-assemble on the thiol-functionalized fibrous silica microspheres to produce a robust catalyst. *J. Phys. Chem. Solids* **2023**, *174*, 111190. https://doi.org/10.1016/j.jpcs.2022.111190.
165. Fu, Y.; Xu, P.; Huang, D.; Zeng, G.; Lai, C.; Qin, L.; Li, B.; He, J.; Yi, H.; Cheng, M.; et al. Au nanoparticles decorated on activated coke via a facile preparation for efficient catalytic reduction of nitrophenols and azo dyes. *Applied Surface Science* **2019**, *473*, 578–588. https://doi.org/10.1016/j.apsusc.2018.12.207.
166. Dong, J.; Tong, J.; Luo, J.; Liu, X. Gold nanoparticles for smart and recoverable catalyst using thermo-responsive core-crosslinked star polymer as the nanoreactor. *Applied Surface Science* **2020**, *507*, 144950. https://doi.org/10.1016/j.apsusc.2019.144950.
167. Zhang, Y.; Hu, B.; Cao, X.M.; Luo, L.; Xiong, Y.; Wang, Z.P.; Hong, X.; Ding, S.Y. β-Cyclodextrin polymer networks stabilized gold nanoparticle with superior catalytic activities. *Nano Research* **2020**, *14*, 1018–1025. https://doi.org/10.1007/s12274-020-3144-7.
168. Hu, C.; Yang, C.; Wang, X.; Wang, X.; Zhen, S.; Zhan, L.; Huang, C.; Li, Y. Rapid and facile synthesis of Au nanoparticle-decorated porous MOFs for the efficient reduction of 4-nitrophenol. *Sep. Purif. Technol.* **2022**, *300*, 121801. https://doi.org/10.1016/j.seppur.2022.121801.
169. Yao, Y.; Zhang, K.; Chen, J.; Li, W.; Zhang, A. Dendronized chitosan-mediated fabrication of Au@AgNPs with visible light trigger. *ACS Sustainable Chem. Eng.* **2022**, *10*, 8265–8274. https://doi.org/10.1021/acssuschemeng.2c00615.
170. Wu, K.; Wang, X.Y.; Guo, L.L.; Xu, Y.J.; Zhou, L.; Lyu, Z.Y.; Liu, K.Y.; Si, R.; Zhang, Y.W.; Sun, L.D.; et al. Facile synthesis of Au embedded CuOx-CeO_2 core/shell nanospheres as highly reactive and sinter-resistant catalysts for catalytic hydrogenation of p-nitrophenol. *Nano Res.* **2020**, *13*, 2044–2055. https://doi.org/10.1007/s12274-020-2806-9.
171. He, J.; Ji, W.; Yao, L.; Wang, Y.; Khezri, B.; Webster, R.D.; Chen, H. Strategy for Nano-Catalysis in a Fixed-Bed System. *Adv. Mater.* **2014**, *26*, 4151–4155. https://doi.org/10.1002/adma.201306157.
172. Xu, Y.; Shi, X.; Hua, R.; Zhang, R.; Yao, Y.; Zhao, B.; Liu, T.; Zheng, J.; Lu, G. Remarkably catalytic activity in reduction of 4-nitrophenol and methylene blue by Fe_3O_4@COF supported noble metal nanoparticles. *Appl. Catal., B* **2020**, *260*, 118142. https://doi.org/10.1016/j.apcatb.2019.118142.
173. Velpula, S.; Beedu, S.R.; Rupula, K. Bimetallic nanocomposite (Ag-Au, Ag-Pd, Au-Pd) synthesis using gum kondagogu a natural biopolymer and their catalytic potentials in the degradation of 4-nitrophenol. *International Journal of Biological Macromolecules* **2021**, *190*, 159–169. https://doi.org/10.1016/j.ijbiomac.2021.08.211.
174. Kong, L.; Guo, Y.; Wang, X.; Zhang, X. Double-walled hierarchical porous silica nanotubes loaded Au nanoparticles in the interlayer as a high-performance catalyst. *Nanotechnology* **2019**, *31*, 015701. https://doi.org/10.1088/1361-6528/ab4401.
175. Li, C.; Xu, S.; Jin, M.; Wan, D. Trace thioether inserted polyamine patches on a support mediate uniform gold nanoclusters as ultrahigh active catalysts. *J. Mater. Chem.A* **2021**, *9*, 15714–15723. https://doi.org/10.1039/d1ta03220h.
176. Liu, J.; Yu, H.; Wang, L. Effective reduction of 4-nitrophenol with Au NPs loaded ultrathin two dimensional metal-organic framework nanosheets. *Appl. Catal., A* **2020**, *599*, 117605. https://doi.org/10.1016/j.apcata.2020.117605.
177. Baral, A.; Cavalieri, F.; Chattopadhyay, S.; Ashokkumar, M. Synthesis of gold nanosheets with controlled morphology by combining a natural amino acid with high-frequency ultrasound. *ACS Sustainable Chem. Eng.* **2021**, *9*, 13953–13962. https://doi.org/10.1021/acssuschemeng.1c05574.
178. Woo, H.; Park, K.H. Hybrid Au nanoparticles on Fe_3O_4@polymer as efficient catalyst for reduction of 4-nitrophenol. *Catal. Commun.* **2014**, *46*, 133–137. https://doi.org/10.1016/j.catcom.2013.12.007.

179. Qiu, Y.; Ma, Z.; Hu, P. Environmentally benign magnetic chitosan/Fe_3O_4 composites as reductant and stabilizer for anchoring Au NPs and their catalytic reduction of 4-nitrophenol. *J. Mater. Chem. A* **2014**, *2*, 13471–13478. https://doi.org/10.1039/c4ta02268h.
180. Zhang, P.; Shao, C.; Li, X.; Zhang, M.; Zhang, X.; Su, C.; Lu, N.; Wang, K.; Liu, Y. An electron-rich free-standing carbon@Au core–shell nanofiber network as a highly active and recyclable catalyst for the reduction of 4-nitrophenol. *Phys. Chem. Chem. Phys.* **2013**, *15*, 10453. https://doi.org/10.1039/c3cp50917f.
181. Huang, J.; Tan, X.; Li, C.; Wu, R.; Ran, S.; Tao, Y.; Mou, T. Green synthesis of Au-NPs on g-C_3N_4 hybrid nanomaterials based on supramolecular pillar[6]arene and its applications for catalysis. *ACS Omega* **2022**, *7*, 18085–18093. https://doi.org/10.1021/acsomega.2c01603.
182. Yang, X.; Lu, P.; Yu, L.; Pan, P.; Elzatahry, A.A.; Alghamdi, A.; Luo, W.; Cheng, X.; Deng, Y. An Efficient Emulsion-Induced Interface Assembly Approach for Rational Synthesis of Mesoporous Carbon Spheres with Versatile Architectures. *Advanced Functional Materials* **2020**, *30*, 2002488. https://doi.org/10.1002/adfm.202002488.
183. Xiong, L.L.; Huang, R.; Chai, H.H.; Yu, L.; Li, C.M. Facile synthesis of Fe_3O_4@tannic acid@Au nanocomposites as a catalyst for 4-nitrophenol and methylene blue removal. *ACS Omega* **2020**, *5*, 20903–20911. https://doi.org/10.1021/acsomega.0c02347.
184. Kuroda, K.; Ishida, T.; Haruta, M. Reduction of 4-nitrophenol to 4-aminophenol over Au nanoparticles deposited on PMMA. *J. Mol. Catal. A: Chem.* **2009**, *298*, 7–11. https://doi.org/10.1016/j.molcata.2008.09.009.
185. Fu, Y.; Huang, T.; Jia, B.; Zhu, J.; Wang, X. Reduction of nitrophenols to aminophenols under concerted catalysis by Au/g-C_3N_4 contact system. *Appl. Catal., B* **2017**, *202*, 430–437. https://doi.org/10.1016/j.apcatb.2016.09.051.
186. Ye, W.; Yu, J.; Zhou, Y.; Gao, D.; Wang, D.; Wang, C.; Xue, D. Green synthesis of Pt–Au dendrimer-like nanoparticles supported on polydopamine-functionalized graphene and their high performance toward 4-nitrophenol reduction. *Appl. Catal., B* **2016**, *181*, 371–378. https://doi.org/10.1016/j.apcatb.2015.08.013.
187. Ilgin, P.; Ozay, O.; Ozay, H. A novel hydrogel containing thioether group as selective support material for preparation of gold nanoparticles: Synthesis and catalytic applications. *Appl. Catal., B* **2019**, *241*, 415–423. https://doi.org/10.1016/j.apcatb.2018.09.066.
188. Fu, J.; Wang, S.; Zhu, J.; Wang, K.; Gao, M.; Wang, X.; Xu, Q. Au-Ag bimetallic nanoparticles decorated multi-amino cyclophosphazene hybrid microspheres as enhanced activity catalysts for the reduction of 4-nitrophenol. *Mater. Chem. Phys.* **2018**, *207*, 315–324. https://doi.org/10.1016/j.matchemphys.2018.01.002.
189. Zheng, Y.; Qi, X.; Xiao, F.; Wang, F.; Wang, N. Spherical covalent organic frameworks supported black phosphorus@Au nanocatalysts for nitrophenol hydrogenation in a high efficiently flow-through process. *Applied Surface Science* **2023**, *611*, 155723. https://doi.org/10.1016/j.apsusc.2022.155723.
190. Balakrishnan, T.; Choi, S.M. Encapsulation of atomically thin gold nanosheets within porous silica for enhanced structural stability and superior catalytic performance. *New J. Chem.* **2022**, *46*, 18699–18709. https://doi.org/10.1039/d2nj03221j.
191. Liu, Y.; Dong, H.; Huang, H.; Zong, W.; Miao, Y.E.; He, G.; Parkin, I.P.; Lai, F.; Liu, T. Electron-deficient Au nanoparticles confined in organic molecular cages for catalytic reduction of 4-nitrophenol. *ACS Appl. Nano Mater.* **2022**, *5*, 1276–1283. https://doi.org/10.1021/acsanm.1c03859.
192. Li, C.; Su, Y.; Cheng, M.; Liu, J.; Hou, S. Gold and cobalt nanoparticles dispersed on N-doped carbon matrix as a catalyst for 4-nitrophenol reduction. *ChemistrySelect* **2022**, *7*. https://doi.org/10.1002/slct.202103739.
193. Zhou, S.; Liu, C.; Jin, W.; Pan, L.; Jiang, Q.; Hu, Y.; Kong, Y. Density functional theory study of small Au nanoparticles anchored on the inner surface of mesoporous Co_3O_4 for the catalytic reduction of 4-nitrophenol. *ACS Appl. Nano Mater.* **2021**, *4*, 4763–4773. https://doi.org/10.1021/acsanm.1c00362.
194. Zhang, Z.; Shao, C.; Zou, P.; Zhang, P.; Zhang, M.; Mu, J.; Guo, Z.; Li, X.; Wang, C.; Liu, Y. In situ assembly of well-dispersed gold nanoparticles on electrospun silica nanotubes for catalytic reduction of 4-nitrophenol. *Chem. Commun.* **2011**, *47*, 3906. https://doi.org/10.1039/c0cc05693f.
195. Wu, X.Q.; Wu, X.W.; Huang, Q.; Shen, J.S.; Zhang, H.W. In situ synthesized gold nanoparticles in hydrogels for catalytic reduction of nitroaromatic compounds. *Appl. Surf. Sci.* **2015**, *331*, 210–218. https://doi.org/10.1016/j.apsusc.2015.01.077.
196. Zhang, Q.P.; ling Sun, Y.; Cheng, G.; Wang, Z.; Ma, H.; Ding, S.Y.; Tan, B.; hua Bu, J.; Zhang, C. Highly dispersed gold nanoparticles anchoring on post-modified covalent organic framework for catalytic application. *Chem. Eng. J.* **2020**, *391*, 123471. https://doi.org/10.1016/j.cej.2019.123471.
197. Nguyen, T.B.; Huang, C.; an Doong, R. Enhanced catalytic reduction of nitrophenols by sodium borohydride over highly recyclable Au@graphitic carbon nitride nanocomposites. *Appl. Catal., B* **2019**, *240*, 337–347. https://doi.org/10.1016/j.apcatb.2018.08.035.
198. Huang, T.; Meng, F.; Qi, L. Facile synthesis and one-dimensional assembly of cyclodextrin-capped gold nanoparticles and their applications in catalysis and surface-enhanced raman scattering. *The Journal of Physical Chemistry C* **2009**, *113*, 13636–13642. https://doi.org/10.1021/jp903405y.
199. Yang, Y.; Zhang, Y.; Wang, T.; Jing, X.; Liu, Y. Gold nanoparticles immobilized in porous aromatic frameworks with abundant metal anchoring sites as heterogeneous nanocatalysts. *ACS Appl. Mater. Interfaces* **2023**, *15*, 9307–9314. https://doi.org/10.1021/acsami.2c20602.
200. Yang, Y.; Yang, Y.; Wang, T.; Tian, Y.; Jing, X.; Zhu, G. Highly selective reduction of nitroarenes with gold nano-catalysts immobilized in porous aromatic frameworks. *Microporous Mesoporous Mater.* **2020**, *306*, 110393. https://doi.org/10.1016/j.micromeso.2020.110393.

201. Rajagopal, V.; Ragunath, M.; Khan, N.A.; Kathiresan, M.; Suryanarayanan, V.; Jones, L.; Kundu, S. Gold nanoparticles decorated covalent organic polymer as a bimodal catalyst for total water splitting and nitro compound reduction. *Materials Today Chemistry* **2023**, *27*, 101327. https://doi.org/10.1016/j.mtchem.2022.101327.
202. Wang, Y.L.; Dai, Y.M.; Tsai, M.H. Highly efficient and recyclable Fe_3C/Au@NG catalyst for 4-nitrophenol reduction. *Catal. Commun.* **2021**, *149*, 106251. https://doi.org/10.1016/j.catcom.2020.106251.
203. Wang, X.; Fu, J.; Wang, M.; Wang, Y.; Chen, Z.; Zhang, J.; Chen, J.; Xu, Q. Facile synthesis of Au nanoparticles supported on polyphosphazene functionalized carbon nanotubes for catalytic reduction of 4-nitrophenol. *J. Mater. Sci.* **2014**, *49*, 5056–5065. https://doi.org/10.1007/s10853-014-8212-5.
204. Hemmati, S.; Heravi, M.M.; Karmakar, B.; Veisi, H. In situ decoration of Au NPs over polydopamine encapsulated GO/Fe_3O_4 nanoparticles as a recyclable nanocatalyst for the reduction of nitroarenes. *Sci. Rep.* **2021**, *11*. https://doi.org/10.1038/s41598-021-90514-x.
205. Dhar, J.; Patil, S. Self-assembly and catalytic activity of metal nanoparticles immobilized in polymer membrane prepared via layer-by-layer approach. *ACS Appl. Mater. Interfaces* **2012**, *4*, 1803–1812. https://doi.org/10.1021/am300068e.
206. Wang, Y.; Wei, G.; Zhang, W.; Jiang, X.; Zheng, P.; Shi, L.; Dong, A. Responsive catalysis of thermoresponsive micelle-supported gold nanoparticles. *J. Mol. Catal. A: Chem.* **2007**, *266*, 233–238. https://doi.org/10.1016/j.molcata.2006.11.014.
207. Li, J.; yan Liu, C.; Liu, Y. Au/graphene hydrogel: synthesis, characterization and its use for catalytic reduction of 4-nitrophenol. *J. Mater. Chem.* **2012**, *22*, 8426. https://doi.org/10.1039/c2jm16386a.
208. Liu, J.; Wang, Z.; Wang, Q.; Zhang, K.; Luo, Y.; Liu, Y.; Lyu, Y.; Huang, B. Porphyrin-based covalent triazine framework and its carbonized derivative as catalyst scaffold of Au and Ag nanoparticles for 4-nitrophenol reduction. *Microporous Mesoporous Mater.* **2022**, *330*, 111611. https://doi.org/10.1016/j.micromeso.2021.111611.
209. Zhou, Y.; Wang, W. Polydopamine nanospheres-grafted-PDMAEMA brushes/Au composites as a thermally adjustable catalyst for the reduction of 4-nitrophenol. *Chem. Lett.* **2022**, *51*, 811–814. https://doi.org/10.1246/cl.220220.
210. Sun, L.; Jiang, L.; Zhang, J.; Murayama, T.; Zhang, M.; Zheng, Y.; Su, H.; Qi, C. Preparation of polyaniline microtubes as the gold catalyst support with improved catalytic performances for the reduction of nitrophenols. *Top. Catal.* **2020**, *64*, 215–223. https://doi.org/10.1007/s11244-020-01385-x.
211. Yu, J.; Niedenthal, W.; Smarsly, B.M.; Natile, M.M.; Huang, Y.; Carraro, M. Au nanoparticles supported on piranha etched halloysite nanotubes for highly efficient heterogeneous catalysis. *Appl. Surf. Sci.* **2021**, *546*, 149100. https://doi.org/10.1016/j.apsusc.2021.149100.
212. Yuan, G.; Sun, J.; Sun, X.; Hu, J.; Han, J.; Guo, R. Rational design of dumbbell-like Au-Fe_3O_4@Carbon yolk@shell nanospheres with superior catalytic activity. *Colloids Surf., A* **2021**, *623*, 126665. https://doi.org/10.1016/j.colsurfa.2021.126665.
213. Zhao, Y.; Bi, S.; Gao, F.; Wang, L. Preparation of Cu_2O/Au composite nanomaterials for effective reduction of 4-nitrophenol. *ChemistrySelect* **2023**, *8*. https://doi.org/10.1002/slct.202204665.
214. Zhang, Q.; Xu, H.; Pan, L.; Kuchmizhak, A.; Wang, L. A highly loaded, excellent recyclable nanosheet catalyst Au@mesoporous SiO_2. *Appl. Organomet. Chem.* **2022**, *37*. https://doi.org/10.1002/aoc.6962.
215. Devi, A.P.; Padhi, D.K.; Mishra, P.M.; Behera, A.K. Bio-surfactant mediated synthesis of Au/g-C_3N_4 plasmonic hybrid nanocomposite for enhanced photocatalytic reduction of mono-nitrophenols. *J. Ind. Eng. Chem.* **2022**, *108*, 118–129. https://doi.org/10.1016/j.jiec.2021.12.030.
216. Manivannan, S.; An, S.; Jeong, J.; Viji, M.; Kim, K. Hematite/M (M = Au, Pd) catalysts derived from a double-hollow prussian blue microstructure: simultaneous catalytic reduction of o- and p-nitrophenols. *ACS Appl. Mater. Interfaces* **2020**, *12*, 17557–17570. https://doi.org/10.1021/acsami.0c01704.
217. Jana, D.; Dandapat, A.; De, G. Anisotropic gold nanoparticle doped mesoporous boehmite films and their use as reusable catalysts in electron transfer reactions. *Langmuir* **2010**, *26*, 12177–12184. https://doi.org/10.1021/la100040m.
218. Nguyen, V.P.; Trung, H.L.; Nguyen, T.H.; Hoang, D.; Tran, T.H. Advancement of microwave-assisted biosynthesis for preparing au nanoparticles using ganoderma lucidum extract and evaluation of their catalytic reduction of 4-nitrophenol. *ACS Omega* **2021**, *6*, 32198–32207. https://doi.org/10.1021/acsomega.1c05033.
219. Cyganowski, P. Fully recyclable gold-based nanocomposite catalysts with enhanced reusability for catalytic hydrogenation of p-nitrophenol. *Colloids Surf., A* **2021**, *612*, 125995. https://doi.org/10.1016/j.colsurfa.2020.125995.
220. Al-Ghamdi, Y.O.; Khan, S.A. Stabilization of zero-valent Au nanoparticles on carboxymethyl cellulose layer coated on chitosan-CBV 780 zeolite Y sheets: assessment in the reduction of 4-nitrophenol and dyes. *Cellulose* **2020**, *27*, 8827–8841. https://doi.org/10.1007/s10570-020-03379-0.
221. Kalyan, I.; Pal, T.; Pal, A. Immobilization of size variable Au nanoparticles on surfactant-modified silica and their catalytic application toward 4-nitrophenol reduction: A comparative account of catalysis. *Surfaces and Interfaces* **2021**, *26*, 101423. https://doi.org/10.1016/j.surfin.2021.101423.
222. Evangelista, V.; Acosta, B.; Miridonov, S.; Smolentseva, E.; Fuentes, S.; Simakov, A. Highly active Au-CeO_2@ZrO_2 yolk–shell nanoreactors for the reduction of 4-nitrophenol to 4-aminophenol. *Appl. Catal., B* **2015**, *166-167*, 518–528. https://doi.org/10.1016/j.apcatb.2014.12.006.
